# Supplementary material for: An Nuclear Magnetic Resonance Fingerprint Matching Approach for the Identification and Structural Re-Evaluation of Pseudomonas Lipopeptides
Source: Microbiol Spectr. 2022 Jul 25;10(4):e01261-22. doi: 10.1128/spectrum.01261-22 (PMC9431178; doi:10.1128/spectrum.01261-22)
Supplement: Supplemental file 1 — Supplemental material. Download spectrum.01261-22-s0001.pdf, PDF file, 3.8 MB [file spectrum.01261-22-s0001.pdf]

Supplementary Information for

An NMR fingerprint matching approach for the identification and structural re-evaluation of Pseudomonas lipopeptides.

Vic De Roo, Yentl Verleysen, Benjámin Kovács, De Vleeschouwer Matthias, Penthip Muangkaew, Léa Girard, Monica Höfte, René De Mot, Annemieke Madder, Niels Geudens, and José C. Martins
Corresponding authors: Niels Geudens and José C. Martins
Email: niels.geudens@ugent.be and jose.martins@ugent.be

This PDF file includes:

Supplementary text
Materials & Methods
Figures S1 to S62
Tables S1 to S29

Table of Contents

ANALYSES METHODS - SCHEMATIC **4**

CLIP GROUP NOMENCLATURE **6**

GENERAL METHODS **6**

SYNTHESIS OF BUILDING BLOCKS 7
SOLID PHASE PEPTIDE SYNTHESIS 7
PRODUCTION AND EXTRACTION OF NATURAL CYCLIC LIPODEPSIPEPTIDES 9

ANALYSIS OF THE NATURAL CLIPS FROM *PSEUDOMONAS AZADIAE* SWRI103 .... **9**

EXTRACTION AND PURIFICATION OF NATURAL CLIP 9
MASS ANALYSIS OF THE NATURAL BANANAMIDE ANALOGUE 11
NMR CHARACTERIZATION OF THE NATURAL BANANAMIDE ANALOGUE 11
STEREOCHEMICAL INVESTIGATION OF BANANAMIDE ANALOGUE 17

SYNTHESIS OF DIFFERENT STEREOCHEMICAL POSSIBILITIES OF THE SWRI103 CLIP **20**

SYNTHESIS OF (8:6) 1L (1) 22
SYNTHESIS OF (8:6) 4L (2) 26
SYNTHESIS OF (8:6) 5L (3) 26

NMR CHARACTERIZATION OF CYCLIC LIPODEPSIPEPTIDES 1-3. .... **27**

NMR CHARACTERIZATION OF CYCLIC LIPODEPSIPEPTIDE (1) 27
NMR CHARACTERIZATION OF CYCLIC LIPODEPSIPEPTIDE (2) 29
NMR CHARACTERIZATION OF CYCLIC LIPODEPSIPEPTIDE (3): 31
NMR CHARACTERIZATION OF CYCLIC LIPODEPSIPEPTIDE 3S-OH C10:0 MDN0066 (COMPOUND 2B): 33

ANALYSIS OF THE NATURAL CLIPS FROM *PSEUDOMONAS AESTUS* CMR5C **36**

EXTRACTION AND PURIFICATION OF ORFAMIDE B 36
NMR CHARACTERIZATION OF THE NATURAL ORFAMIDE B 36

STEREOCHEMICAL ANALYSIS OF THE NATURAL CLIPS FROM *PSEUDOMONAS AESTUS* CMR5C **39**

SYNTHESIS OF DIFFERENT STEREOCHEMICAL POSSIBILITIES OF CMR5C-ORFAMIDE. **40**

SYNTHESIS OF (10:8) 1L (4) 42
SYNTHESIS OF (10:8) 5L (5) 47
SYNTHESIS OF (10:8) L1L5 (6) 49

NMR CHARACTERIZATION OF CYCLIC LIPODEPSIPEPTIDES 4-6 .... **52**

ANALYSIS OF PEPTIDE (10:8)-1L (4) 52
ANALYSIS OF PEPTIDE (10:8)-5L (5) 55
ANALYSIS OF PEPTIDE (10:8)-1L5L (6) 58

ANALYSIS OF THE NATURAL ORFAMIDE A FROM *PSEUDOMONAS PROTEGENS* PF-5 .... **60**

EXTRACTION AND PURIFICATION OF NATURAL CLIP 60

NMR CHARACTERIZATION OF ORFAMIDES EXTRACTED FROM *P. PROTEGENS* PF-5 62

NMR FINGERPRINT MATCHING WITH ORFAMIDE B FROM *PSEUDOMONAS* SP. F6 73

ANALYSIS OF THE NATURAL ORFAMIDE B FROM *PSEUDOMONAS SESSILINIGENES* CMR12A 73

EXTRACTION AND PURIFICATION OF NATURAL CLIP 73
NMR CHARACTERIZATION OF ORFAMIDES EXTRACTED FROM *P. SESSILINIGENES* CMR12A 74

ANALYSIS OF THE NATURAL ORFAMIDE A FROM *PSEUDOMONAS* SP. PH1B 77

EXTRACTION AND PURIFICATION OF NATURAL CLIP 77
NMR CHARACTERIZATION OF ORFAMIDES EXTRACTED FROM *PSEUDOMONAS* SP. PH1B 77

STEREOCHEMICAL ANALYSIS OF ORFAMIDE A, EXCRETED BY *P. PROTEGENS* PF-5 82

ANALYSIS OF XANTHOLYSIN A FROM *PSEUDOMONAS MOSSELII* BW11M1 83

EXTRACTION AND PURIFICATION OF NATURAL CLIP 83
STEREOCHEMICAL ANALYSIS OF XANTHOLYSIN A, EXCRETED BY *P. MOSSELII* BW11M1 83
MULTIDISCIPLINARY DATA OF XANTHOLYSIN A FROM *P. MOSSELII* BW11M1 84

SYNTHESIS OF XANTHOLYSIN A 86

NMR CHARACTERIZATION OF SYNTHETIC XANTHOLYSIN (15) 90

QUANTIFYING THE (DIS)SIMILARITY IN NMR FINGERPRINTS 96

REFERENCES 98

Analyses methods - schematic

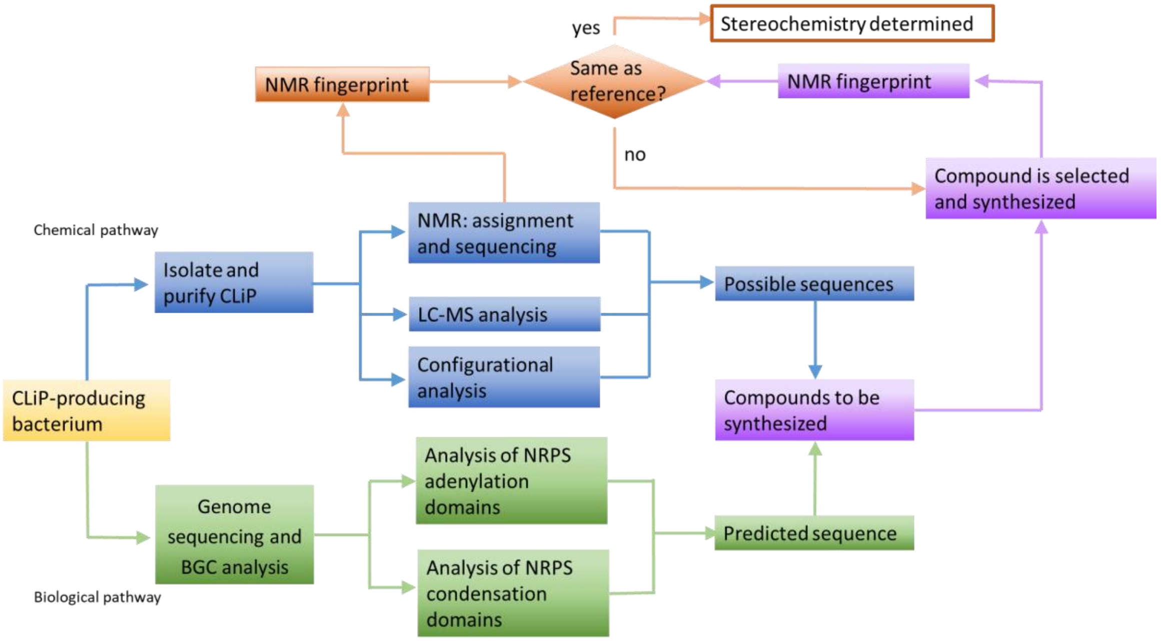

Figure S1: Overview of the analysis steps for the stereochemical validation of CLiPs, as used in this report.

A	Bacteria	references	Analyses performed			
			Amino acid sequence	Fatty acid	Ester bond	Stereochemistry
MDN-0066	*Pseudomonas granadensis* F-278,770^T	Cautain, 2015	MS/MS and NMR	MS and NMR	NMR	Marfey's, but stereochemical dead-end
Orfamide A	*Pseudomonas protegens* Pf-5	Gross, 2007	Genomics, NMR and MS/MS	MS, GC an NMR, but error in stereochemistry	NMR	Chiral GC, but error
Orfamide A	*Pseudomonas sp.* PH1b	Chan, 2016	n.d.	n.d.	n.d.	n.d.
Orfamide A	*Pseudomonas* sp. F6	Jang, 2013	NMR	MS, no stereochemistry	n.d.	n.d.
Orfamide B	*Pseudomonas sp.* PH1b	Gross, 2007	MS/MS and NMR	MS	Homology with orfamide A	Homology with orfamide A
Orfamide B	*Pseudomonas aestus* CMR5c	Ma, 2016	NMR and MS	NMR and MS, no stereochemistry	NMR	n.d.
Orfamide B	*Pseudomonas sessilinigenes* CMR12a	D'Aes, 2014	Genomics	n.d.	n.d.	n.d.
Orfamide B	*Pseudomonas sp.* PH1b	Chan, 2016	n.d.	n.d.	n.d.	n.d.
Xantholysin	*Pseudomonas mosselii* BW11M1	Li, 2012	Genomics and NMR	MS and NMR	NMR	n.d.
Xantholysin	*Pseudomonas* sp. 250J	Molina-Santiago, 2015	NMR and MS	NMR and MS	NMR and MS	Marfey's, but stereochemical dead-end
Xantholysin	*Pseudomonas* sp. DJ15	Lim, 2017	^{1}H NMR	^{1}H NMR and MS	Homology with xantholysin A	n.d.
Xantholysin	*Pseudomonas* sp. COR51	Oni, 2019	NMR and MS	NMR and MS	NMR	n.d.
Xantholysin	*Pseudomonas xantholysinigenes*	Girard, 2021	NMR and MS	NMR and MS	NMR	n.d.

B	Bacteria	references	Analyses performed			
			Amino acid sequence	Fatty acid	Ester bond	Stereochemistry
MDN-0066	*Pseudomonas azadiae* SWRI103^T	This study	MS/MS and NMR	MS and NMR	NMR	Synthesis and NMR fingerprint matching
Orfamide A	*Pseudomonas protegens* Pf-5	This study	Genomics, NMR and MS	MS and NMR	NMR	NMR fingerprint matching
Orfamide A	*Pseudomonas sp.* PH1b	This study	Genomics, NMR and MS	MS and NMR	NMR	NMR fingerprint matching
Orfamide A	*Pseudomonas* sp. F6	This study	NMR	NMR	NMR	NMR fingerprint matching
Orfamide B	*Pseudomonas sp.* PH1b	This study	MS/MS and NMR	MS	NMR	NMR fingerprint matching
Orfamide B	*Pseudomonas aestus* CMR5c	This study	NMR and MS	NMR and MS	NMR	NMR fingerprint matching
Orfamide B	*Pseudomonas sessilinigenes* CMR12a	This study	Genomics	NMR and MS	NMR	NMR fingerprint matching
Orfamide B	*Pseudomonas sp.* PH1b	This study	NMR and MS	NMR and MS	NMR	NMR fingerprint matching
Xantholysin	*Pseudomonas mosselii* BW11M1	This study	Genomics and NMR	MS and NMR	NMR	NMR fingerprint matching
Xantholysin	*Pseudomonas* sp. 250J	This study	NMR and MS	NMR and MS	NMR	NMR fingerprint matching
Xantholysin	*Pseudomonas* sp. DJ15	This study	NMR	NMR and MS	NMR	NMR fingerprint matching
Xantholysin	*Pseudomonas* sp. COR51	This study	NMR and MS	NMR and MS	NMR	NMR fingerprint matching
Xantholysin	*Pseudomonas xantholysinigenes*	This study	NMR and MS	NMR and MS	NMR	NMR fingerprint matching

Figure S2: Structural and stereochemical knowledge of CLiPs produced by various bacterial species before (A) and after (B) the application of the NMR fingerprint methodology. (1-11)

CLiP group nomenclature

With well over 100 distinct *Pseudomonas* CLiPs reported to date each with their own trivial name, we find it convenient to formally propose a simple and convenient (ℓ: m) notation that will be used throughout to classify structurally homologous CLiPs in particular groups named for a member that represents the group prototype. It is based on 1) the total number of amino acid residues or 'length' ℓ of the peptide sequence and 2) the number of amino acids m contained within the macrocycle, to assist in their classification. Using this, a total of 17 sequences featuring a nonapeptide (ℓ=9) chain with seven residues forming the macrocycle (m=7) and amphipathic profile matching that of viscosin together define the (9:7) Viscosin group. In this way, individual CLiPs or groups of CLiPs introduced over time by different authors such as the PPZPMs and poaeamides can structurally be accommodated in the Orfamide group, all being (10:8) CLiPs with identical amphipathic profile. Similarly, MDN-0066, a CLiP issued from screening *Pseudomonas* sp. against a cancer cell platform, is a (8:6) CLiP that can be considered as a structural homologue of the Bananamide group.

General Methods

General. The 2-chlorotrityl chloride linked polystyrene resin (2-CTC resin) (1.60 mmol/g), Rink Amide resin (0.69 mmol/g), all L-amino acids, Fmoc-D-Val-OH, Fmoc-D-Gln(Trt)-OH, HBTU, TFA, DIC and Allyloxycarbonyl succinimidyl ester (Alloc-OSu) were purchased from Iris Biotech GmbH. HOBt, dry DIPEA, piperidine, phenylsilane, triisopropylsilane, tetrakistriphenylphosphine palladium (0), (cod)Ru(2-methylallyl)$_2$, (R)-BINAP, Nα-(2,4-Dinitro-5-Fluorophenyl)-L-alaninamide, L-amino standards and allylbromide were obtained from Sigma Aldrich (Saint Louis, MO, U.S.A.) and HATU and the D-amino acids including Fmoc-D-Leu-OH, Fmoc-D-Glu(tBu)-OH, Fmoc-D-allo-Thr(OH)-OH and Fmoc-D-Ser(tBu)-OH were ordered from Chem-Impex International, Inc (Wood Dale, IL, U.S.A.). Octanoyl chloride, DMAP and 1-methylimidazole were purchased from Acros Organics (Geel, Belgium) and hydrobromic acid was obtained from Janssen Chimica (Beerse, Belgium). Peptide grade NMP and DMF obtained from Biosolve (Valkenswaard, The Nederlands) were used throughout the on-resin synthesis and washing of the resin. Dry THF, DMF, MeOH, pyridine and all other solvents were purchased from Acros Organics and used without further purification or drying, except for dichloromethane, which was distilled from CaH_2.

LC-MS analysis was performed on an Agilent (Santa Clara, CA, U.S.A) 1100 Series HPLC with an ESI detector type VL, equipped with a Kinetex column (C18, 150 × 4.60 mm, 5 µm particle size) with a flow rate of 1.5 ml/min. Two different solvent systems were used, either 5 mM NH4OAc in H_2O (A) and CH_3CN (B) or 01% HCOOH in H_2O (A) and CH_3CN (B). Optimized different gradients were used for each CLiP.

High-resolution mass spectra for all natural CLiPs were recorded on a 6220 A TOF-MS (Agilient Technologies) equipped with an ESI/ASPCI multimode source (Agilient Technologies) set to APCI mode only.

Semi-preparative purification was performed on an Agilent 218 solvent delivery system with a UV-VIS dual wavelength detector using a Phenomenex column (AXIA packed Luna C18(2), 250 × 21.2 mm, 5 µm particle size) with a flow rate of 17.5 ml/min and following solvent systems: H2O containing 0.1% TFA (A) and CH_3CN (B).

Preparative purification of the crude peptides via RP-preparative-HPLC was performed on a Gilson PLC 2250 instrument with a Waters Millipore Corporation DeltaPak C18 PrepPak cartridge (pore

size 100 Å and spherical silica of 15 µM) connected to a multiwavelength detector. The solvents used for the measure of samples are H_2O (A) and MeCN (B), both containing 0.1%TFA, at a flow rate of 65 mL min^{-1}.

NMR characterization of intermediate products: ^{1}H NMR and ^{13}C NMR were recorded in $CDCl_3$ on a Bruker Avance spectrometer equipped with a 5 mm BBO probe and operating at 300 MHz and 75.77 MHz respectively. High-resolution mass spectra were recorded on an Agilent 6220A time-of-flight mass spectrometer, equipped with an Agilent ESI/APCI multimode source. The ionization mode was set to APCI (atmospheric pressure chemical ionization), while the mass spectra were acquired in 4 GHz high-resolution mode with a mass range set to 3200 Da.

NMR characterization of the final compounds were performed either on a Bruker (Billerica, MA, U.S.A) Avance III spectrometer operating at a frequency of 500.13 MHz and 125.76 MHz for ^{1}H and ^{13}C respectively and equipped with a 5 mm BBI-Z probe or on a Bruker Avance II spectrometer operating at a frequency of 700.13 MHz and 176.05 MHz (^{1}H and ^{13}C respectively.) equipped either a ^{1}H, ^{13}C, ^{15}N TXI-Z probe or 5 mm Prodigy TCI probe. All NMR measurements on the final compounds were performed on lipopeptide solutions with 600 µl of CD_3CN, DMF-$_{d7}$, methanol-$_{d3}$ or DMSO-$_{d7}$(Eurisotop, Saint-Aubin, France). ^{1}H and ^{13}C chemical shift scales were calibrated by using the residual solvent signal. High quality HP-7 (New Era Ent. Inc) NMR tubes were used. Sample temperature was set to 25°C unless stated otherwise. All spectra were processed and analysed using TOPSPIN 4.0.8 and were referenced based on the used solvent.

Full assignment of the peptides was achieved through liquid state NMR spectroscopy using 1D ^{1}H, 2D ^{1}H-^{1}H COSY, ^{1}H-^{1}H TOCSY with 90 ms spinlock, ^{1}H-^{1}H off-resonance ROESY with a mixing time of 200 ms and gradient-enhanced ^{1}H-^{13}C HSQC and ^{1}H-^{13}C HMBC optimised for a $^{n}J_{CH}$ coupling of 6.5 Hz. The spectral width was set to 14 ppm in the ^{1}H dimension and 90 ppm (gHSQC) or 200 ppm (gHMBC) along the ^{13}C dimension and, generally, 2048 data points were sampled in the direct dimension and 512 data points in the indirect dimension. Standard pulse sequences available in the Bruker library were used throughout with excitation sculpting when applicable. For 2D processing, the spectra were zero filled to a 2048 × 2048 real data matrix and all spectra were multiplied with a squared cosine bell function in both dimensions or a sine bell in the direct dimension for gHMBC before Fourier transformation.

Synthesis of building blocks

The building blocks necessary for the synthesis of the bananamide analogues, orfamide B and xantholysin A, including Fmoc D-Gln(NH_2)-OAll, Fmoc-D-Ser(OH)-OAll, Alloc-L-Ile, Alloc-L-Leu and the lipid tail ((*R*)-3-(tert-butyldimethylsilyloxy)decanoic acid) were synthesized according to the methods described in literature (12). The enantiomeric excess of the lipid tail was determined using chiral HPLC analysis run on a Daicel Chiracel ODH kolom (250x4.6 mm) with an isocratic elution of hexane/EtOH (97/3) over 30 minutes and a flow rate of 1 ml/min. An enantiomeric excess of 99.0% of the (*R*)-enantiomer was established after the asymmetric hydrogenation step. The obtained NMR spectral data were in accordance with earlier reported data (13).

Solid Phase Peptide Synthesis

Automated peptide synthesis was performed in plastic reaction vessels equipped with Teflon frits (MultiSyn Tech GmbH (Witten, Germany)). The automated peptide synthesis was executed with a SYRO II Multiple Peptide Synthesizer Robot, equipped with a vortex unit, at room temperature. The solid phase peptide synthesis made use of the ubiquitous Fmoc/tBu protecting strategy with

HBTU/DIPEA as coupling reagents. The Fmoc deprotection step was performed by treatment with a solution of 40% piperidine in NMP(v/v) for 4 minutes and repeated for another 12 minutes. The coupling step was carried out on the resin using Fmoc protected amino acids (5 equiv.) dissolved in DMF in the presence of HBTU (5 equiv) and DIPEA (10 equiv.) for 40 minutes at room temperature. The coupling step was performed twice to ensure complete coupling of the D-amino acids.

For a manual removal of the Fmoc group, the resin was typically treated with 20% piperidine in DMF (v/v) (10 ml/g resin) for 2 after which the reaction mixture was filtered and the resin was again treated subsequently 10 and 20 minutes with 20% piperidine in DMF. Subsequently, the resin was washed with DMF, MeOH and DCM (6×10s, 10 ml/g resin)

For manual coupling of a building block, 5 equiv. of Fmoc protected amino acid in DMF (0.5 M), 5 equiv. HBTU in DMF (0.5 M) and 10 equiv. DIPEA (2 M in DMF) were added to the resin. After 2 hours of shaking, the reagent was filtered and the resin was washed. The outcome of the reaction can be monitored with a color test, but mostly a small scale cleavage and subsequent LC-MS analysis on the obtained peptide was preferred.

For manual coupling of the lipid tail, 7 equiv. of the fatty acid, 5 equiv. of HBTU and 10 equiv. of DIPEA were added together and dissolved in 2 mL DMF. The mixture was sonicated and added to the resin. After 2 hours of shaking, the reagent was filtered and the resin was washed. The outcome was monitored with a small scale cleavage and subsequent LC-MS analysis on the obtained peptide.

Intermediate compounds were analyzed by subjecting a small fraction of the peptidyl resin to an acidic cleavage. The released peptide was then analyzed by LC-MS. The following small-scale cleavage conditions with TFA were typically applied: 1 mg of peptidyl resin was brought in a plastic reactor vessel equipped with a Teflon frit. A mixture of TFA/triisopropylsilane/water (95/2.5/2.5 %v/v/v) was added and the vessel was shaken for 30 minutes. The resin was washed with DCM (1 ml) and the combined filtrates were dried using an Argon stream to obtain the crude peptide. Subsequently, the peptides are precipitated in cold MTBE while the protecting group debris remains dissolved. The peptides that dissolved in cold MTBE were precipitated in hexane.

Two different procedures were used for the final cleavage and deprotection step. In the first procedure a solution of TFA/TIS/H_2O (95/2.5/2.5 (v/v/v); 10 ml/g resin) was added to the resin and shaken for 15 minutes. The mixture is filtered and the resin is washed 3 times with 1 ml TFA. The filtrate is collected in a Falcon® tube. The cleavage reaction is repeated twice and the combined filtrates were dried (using Ar or N_2) to obtain the crude peptide. Thereafter, cold MTBE was added to precipitate the peptidic compounds present in the crude mixture. Precipitation of all peptidic compounds was ensured by 8 minutes of centrifugation (5000 r.p.m). The supernatant, only containing the protecting group debris, was decanted afterwards. This precipitation step was repeated at least three times. The remaining MTBE is evaporated with a gentle Ar-stream.

In the second procedure a solution of 0.1 M HCl in HFIP (equivalent to 1 ml of ca. 37% aq. HCl per 99 ml HFIP) was used to which 1% v/v TIS was added as scavenger. This solution was added to the resin (10 ml/g resin) and reacted for one hour. Then the reaction mixture was filtered and the resin was washed two times with 0.1 M HCl in HFIP. All filtrates were collected in a Falcon® tube. The cleavage reaction and washing steps were repeated three times. After a total time of 4h, the combined filtrates were dried using Ar to obtain the crude peptide. An identical workup procedure with MTBE was followed to remove protecting groups. The removal of all protecting groups was

verified using LC-MS analysis. In case of incomplete removal, the crude peptide was treated with the cleavage solution for another 3 to 4 hour and again dried using an Argon stream, followed by precipitation in MTBE.

Production and extraction of natural cyclic lipodepsipeptides

A streak of bacterial cells of *Pseudomonas* sp. were transferred into 4 × 5 mL King's Broth medium (Difco Laboratories, Sparks, MD, USA) and grown for 1 day under equilibrated conditions (29 °C, 140 rpm shaking frequency). Afterwards, each of the 5 mL cultures were transferred into a 2L Erlenmeyer flask containing 400 mL of KB or M9 minimal medium. Following an additional 24 h cultivation under minimal conditions (28 °C, 150 rpm shaking frequency), the culture was centrifuged to separate the supernatant from the cells.

The supernatant was acidified towards a pH of 2 with use of a 2M HCl solution and was kept at 5°C overnight. Subsequent, the supernatant was once again subjected to centrifugation to separate the collect the precipitated peptide products. With use of a 2M NaOH solution, the pH was set to 8 and the solution was freeze-dried overnight. The resulting crude mixture was dissolved in a minimal amount of methanol prior to purification.

Extraction of the cells was carried out by adding 5ml of an EtOAc/MeOH mixture (50:50) to the cell pellets. The resulting cell solution was subjected to three cycles of freeze-thawing. After subsequent centrifugation, the supernatant was collected and evaporated under high vacuum.

The combined crude fractions obtained from both supernatant and cells were dissolved in MeOH and subjected to RP-HPLC purification.

Analysis of the natural CLiPs from *Pseudomonas azadiae* SWRI103

Extraction and purification of natural CLiP

After growth of *P. azadiae* SWRI103, the cyclic lipopeptide produced by this bacterial strain was extracted from the supernatant and cell content. In total 5 fractions were caught and examined by mass analysis, with only one showing peptide properties. This peptide fraction was purified via semi-preparative RP- HPLC analysis, using a two solvent system: H_2O + 0.1% TFA (A) and CH_3CN (B), via application of a linear gradient over 15 minutes going from 25:75 to 0:100 (A:B). The main compound eluted at a retention time of 12.3 min .A total of 6.5 mg of pure peptide was obtained. .(Figure S3)

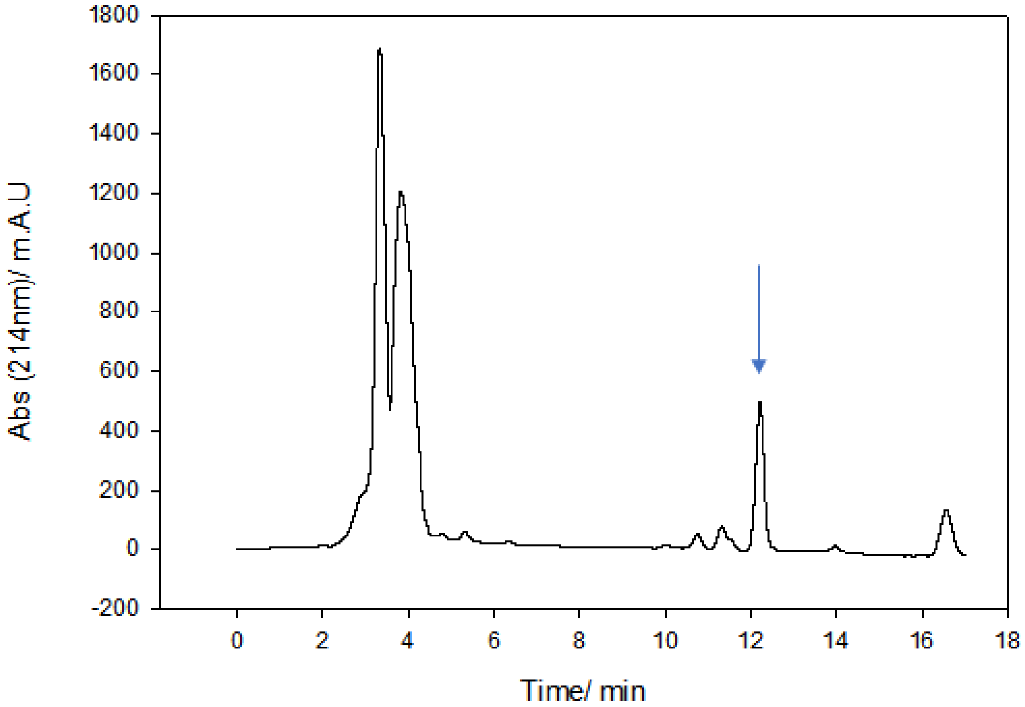

Figure S3: Semi-preparative reversed-phase HPLC chromatogram of *P. azadiae* SWRI103, showing the clear presence of MDN-0066 at 12.3 minutes.

Mass analysis of the natural bananamide analogue

LC-MS analysis confirmed that the main compound found in the crude mixture of the cells was identical to the compound found in the medium extract. HR-MS measurements of the main compound revealed the exact mass of the molecular ion to be 1053.6774 ±0.05Da which agrees to a formula of $C_{52}H_{92}N_8O_{14}$.

NMR characterization of the natural bananamide analogue

The molecular structure of the isolated peptide was revealed by solution state NMR. In acetonitrile-$_{d3}$, full assignment of the ^{1}H and ^{13}C resonances was achieved (see Table I.). The characteristic 2D ^{1}H-^{1}H TOCSY patterns revealed the identity of the amino acids (Figure S5) and the 3-hydroxy decanoic acid fatty acid spin system whereas the 2D ^{1}H-^{1}H ROESY spectrum revealed the connectivity of the residues through observation of either H^{N} (i)→ H^{N} (i+1) or H^{N} (i) to H^{α} (i-1) characteristic cross peaks. The carbonyl ^{13}C resonances and the lactone bond between the hydroxyl functionality of Thr3 and Ile8 could be established by analysing the ^{1}H-{^{13}C} gHMBC.

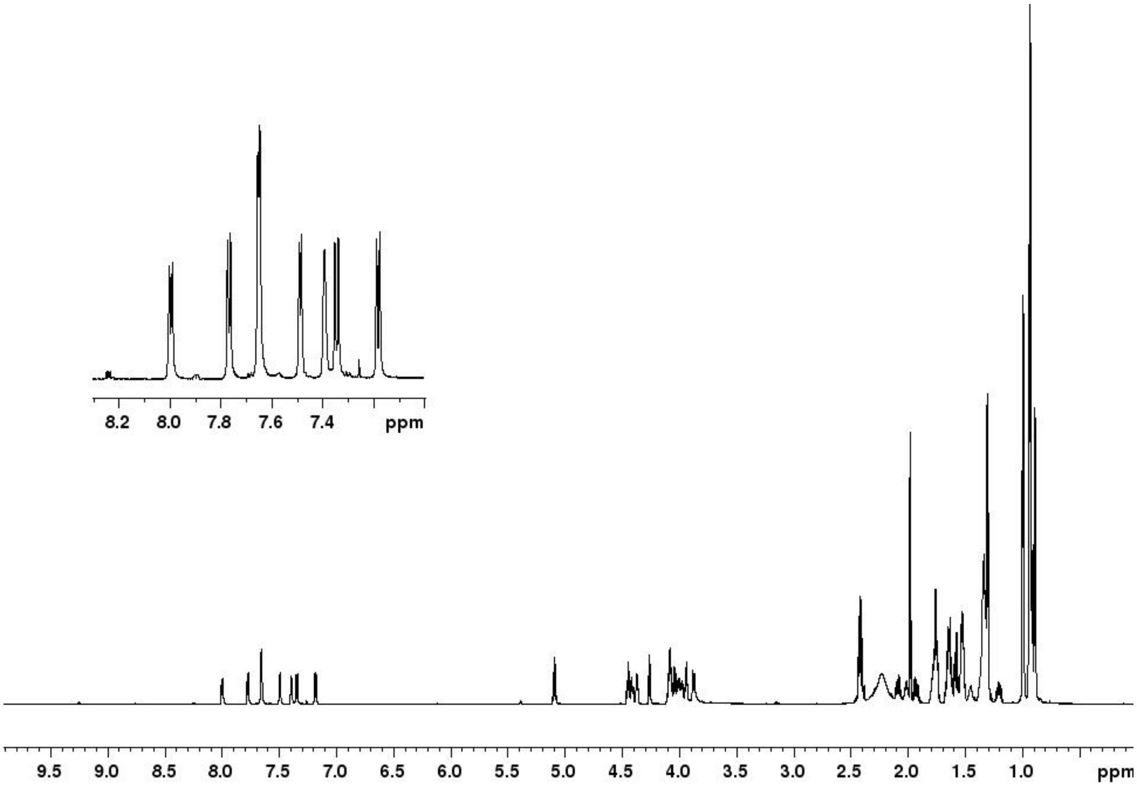

Figure S4: 1D ^{1}H spectrum of the purified natural bananamide analogue MDN-0066 (acetonitrile-d3, 303K , 700 MHz)

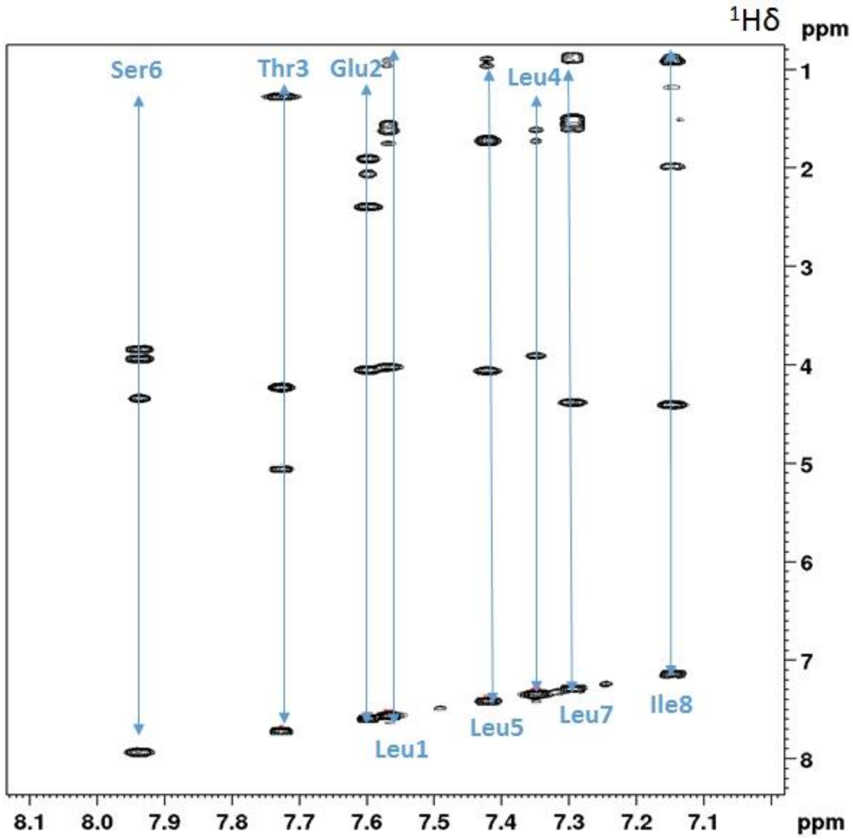

Figure S5: ^{1}H-^{1}H TOCSY traces of the individual amino acids in MDN-0066 as isolated from *P. azadiae* SWRI103.(700MHz, acetonitrile-*d3*, 303K)

Table S1: NMR assignment (^{1}H and ^{13}C) of MDN-0066, extracted as a major compound produced by *P. azadiae* SWRI103. (500MHz, acetonitrile-d3, 303K)

		1H δ [ppm]	13C δ [ppm]			1H δ [ppm]	13C δ [ppm]
**D-Leu1**				**D-Leu5**			
	NH	7.57			NH	7.42	
	CHα	4.02	55.42		CHα	4.05	54.35
	CH2β1	1.54	40.30		CH2β1	1.72	40.07
	CH2β2	1.61	40.30		CH2β2	1.72	40.07
	CHγ	1.74	25.43		CHγ	1.72	25.26
	CH3δ	0.90	22.99		CH3δ	0.89	20.72
	CH3δ	0.96	22.98		CH3δ	0.96	?
	CO	/	174.50		CO	/	173.20
**D-Glu2**				**D-Ser6**			
	NH	7.59			NH	7.92	/
	CHα	4.05	55.41		CHα	4.34	57.84
	CH2β1	2.06	26.21		CH2β1	3.94	62.30
	CH2β2	1.91	26.24		CH2β2	3.84	62.30
	CH2γ1	2.39	31.06		OH		
	CH2γ2	2.39	31.06		CO		170.10
	OH						
	CO		172.70				
**D-aThr3**				**L-Leu7**	NH	7.29	
	NH	7.72			CHα	4.38	53.85
	CHα	4.22	59.83		CH2β1	1.55	43.85
	CHβ	5.06	71.46		CH2β2	1.49	43.74
	CH3γ1	1.27	18.49		CHγ	1.61	25.10
	CH3γ2	1.27	18.84		CH3δ	0.90	21.67
	CO		171.40		CH3δ	0.85	21.59
**L-Leu4**	NH	7.34			CO		170.11
	CHα	3.91	55.05	**L-Ile8**	NH	7.15	
	CH2β1	1.72	39.99		CHα	4.40	57.95
	CH2β2	1.62	40.39		CH β	1.98	37.51
	CHγ	1.74	25.48		CH3γ	0.90	15.30
	CH3δ	0.90	22.99		CH2 γ	1.48	25.30
	CH3δ	0.96	22.98		CH2 γ	1.51	25.30
	CO		174.35		CH3 δ	0.80	11.51
**FA**					CO		172.12
	CO		174.90				
	C-2'	2.37	44.01				
	C-3'	3.98	70.27				
	OH	/					
	C-5'	1.49	38.22				
	C-6'	1.42	26.30				
	C-7'	1.33	26.30				
	C-8'	1.28	32.50				
	C-9'	1.27	32.60				

Table S2: NMR assignment (^{1}H and ^{13}C) of MDN-0066, extracted as a major compound produced by *P. azadiae* SWRI103. (500MHz, DMSO-d6, 298K)

		^{1}H δ [ppm]	^{13}C δ [ppm]			^{1}H δ [ppm]	^{13}C δ [ppm]
**D-Leu1**				**D-Leu5**			
	NH	8.17			NH	**9.02**	
	CHα	**4.22**	51.84		CHα	4.01	53.06
	CH2β1	1.44	40.2		CH2β1	1.61	39.2
	CH2β2	1.44	40.2		CH2β2	1.68	39.2
	CHγ	1.65	24.2		CHγ	0.91	24.2
	CH3δ	0.89	21.5		CH3δ	0.79	23.1
	CH3δ	0.83	21.5		CH3δ	0.79	20.4
	CO				CO		n.d.
**D-Glu2**				**D-Ser6**			
	NH	8.02			NH	7.94	
	CHα	**4.13**	53.2		CHα	4.23	57.42
	CH2β1	1.77	26.4		CH2β1	3.75	60.41
	CH2β2	1.92	26.4		CH2β2	3.72	60.42
	CH2γ1	2.24	30.2		OH	n.d	
	CH2γ2	2.24	30.2		CO		n.d
	OH		n.d				
	CO		n.d				
**D-aThr3**				**L-Leu7**	NH	7.57	
	NH	8.12			CHα	4.29	52.59
	CHα	4.21	58.7		CH2β1	1.40	42.71
	CHβ	5.03	60.4		CH2β2	1.40	42.71
	CH3γ1	1.14	17.7		CHγ	1.58	23.8
	CH3γ2	1.14	17.7		CH3δ	0.83	24.1
	CO				CH3δ	0.79	21.8
					CO		
**L-Leu4**	NH	7.58		**L-Ile8**	NH	7.16	
	CHα	4.13	53.06		CHα	4.31	56.77
	CH2β1	1.49	40.6		CH β	1.93	36.70
	CH2β2	1.49	40.6		CH3γ	24.71	1.12
	CHγ	1.51	24.2		CH2 γ	10.92	0.82
	CH3δ	0.83	22.1		CH2 γ	14.8	0.84
	CH3δ	0.87	22.1		CH3 δ	0.84	11.39
	CO		n.d.		CO		
**FA**							
	CO		n.d				
	C-2'	2.25	43.7				
	C-3'	3.80	68.1				
	OH						
	C-5'	1.24	25.1				
	C-6'	1.22	28.7				
	C-7'	1.22	28.6				
	C-8'	1.23	31.3				
	C-9'	1.26	21.8				

Table S3: NMR assignment (^{1}H and ^{13}C) of MDN-0066, extracted as a major compound produced by *P. azadiae* SWRI103. (500MHz, DMF-d7, 298K)

		**^{1}H δ [ppm]**	**^{13}C δ [ppm]**			**^{1}H δ [ppm]**	**^{13}C δ [ppm]**
**D-Leu1**				**D-Leu5**			
	NH	9.04			NH	7.58	
	CHα	4.11	53.58		CHα	4.19	54.42
	CH2β1	1.87	40.81		CH2β1	1.75	43.58
	CH2β2	1.74	40.81		CH2β2	1.61	43.58
	CHγ	0.96	n.d		CHγ	0.96	23.16
	CH3δ	0.88	20..38		CH3δ	0.86	21.38
	CH3δ	0.88	20.38		CH3δ	0.86	21.38
	CO		n.d		CO		n.d
**D-Glu2**				**D-Ser6**			
	NH	8.16			NH	8.17	
	CHα	4.23	54.21		CHα	4.43	58.39
	CH2β1	1.29	29.54		CH2β1	4.01	61.53
	CH2β2	1.29	29.54		CH2β2	3.94	61.53
	CH2γ1	2.12	26.53		OH	n.d	
	CH2γ2	1.93	26.53		CO		n.d
	OH						
	CO						
**D-aThr3**				**L-Leu7**	NH	7.42	
	NH	8.17			CHα	4.51	53.11
	CHα	4.34	59.37		CH2β1	1.62	40.41
	CHβ	5.17	71.39		CH2β2	1.51	40.42
	CH3γ1	1.26	18.24		CHγ	0.91	n.d
	CH3γ2	1.26	18.24		CH3δ	0.88	21.29
	CO		n.d		CH3δ	0.88	21.29
					CO		
**L-Leu4**	NH	8.58		**L-Ile8**	NH	7.24	
	CHα	4.25	53.81		CHα	4.47	57.47
	CH2β1	1.81	40.72		CH β	2.05	37.21
	CH2β2	1.63	40.72		CH3γ	0.97	23.20
	CHγ	1.56	24.12		CH2 γ	1.21	25.28
	CH3δ	0.84	20.92		CH2 γ	1.21	25.28
	CH3δ	0.84	20.92		CH3 δ	0.87	20.47
	CO		n.d		CO		
**FA**							
	CO						
	C-2'		n.d				
	C-3'	2.43	43.99				
	OH	4.02	69.12				
	C-5'	n.d					
	C-6'	n.d	n.d				
	C-7'	n.d	n.d				
	C-8'	n.d	n.d				
	C-9'						

Stereochemical investigation of bananamide analogue

To 1.3 mg of peptide, 1.5 ml of a 6M HCl solution was added. The solution was stirred for 22h at a temperature of 90°C, fully hydrolyzing the peptide. Subsequently, the HCl was evaporated with a gentle stream of Ar. The derivatization step was performed by adding 300 µL 1M $NaHCO_3$ and 200µL of a 1% (w/w) solution of 1-fluoro-2-4-dinitrophenyl-5-L-alanine amide (L-FDAA) in acetone to the crude hydrolysis residue. The resulting mixture was let to react for an hour at 40°C. The reaction was quenched by 10µL of a 2M HCl solution. The solution was evaporated with use of a SpeedVac (ThermoFischer). Afterwards, the dried compounds were dissolved in 200µL DMSO of which 20µL was taken out and was diluted to 200 µL with use of a 60:40 ammonium acetate (5mM): acetonitrile buffer prior to injection.

Reversed-phase HPLC analysis of the derivatized hydrolysate was performed on an Agilent Technologies 1260 Infinity II instrument with a Kinetex RP-18 (C18, 150 × 4.60 mm, 5 µm particle size) column and connected to a multiwavelength detector. The solvents used for the measure of samples are 0.1% TFA in H_2O (A) and MeCN (B) at a flow rate of 3 mL min^{-1}. After sample injection, the column was flushed with 100% A for 3 min, followed by an isothermal (50°C) gradient from 0 to 100 % B over 120 min and subsequent flushing with 100 % B for 3 min. The detector was set to 340 nm to selectively monitor the derivatives.

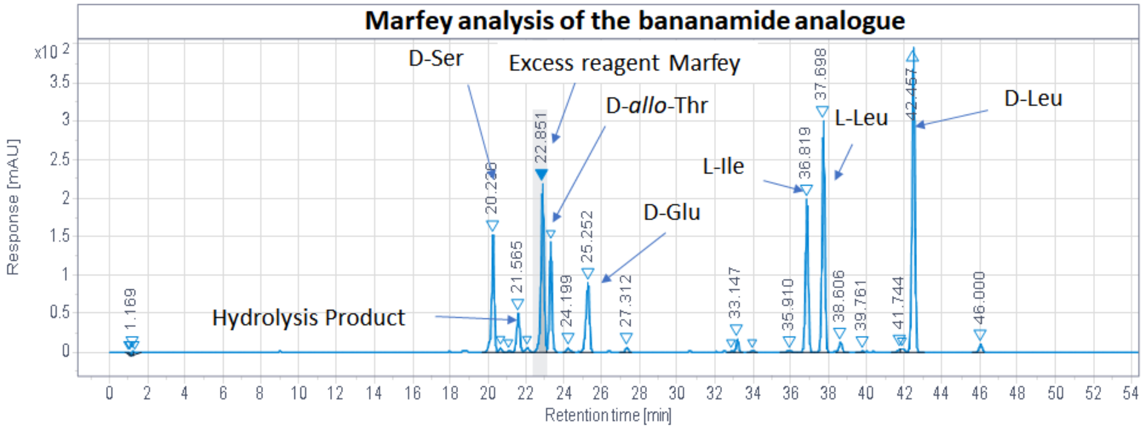

Figure S6: Chromatogram obtained by injection of the derivatized hydrolysate of the bananamide analogue. Identity of the amino-acids was established by co-injection of standards. Conditions are mentioned above.

Table S4: Summary of the Marfey analysis concerning the lipopeptide produced by *P. azadiae* SWRI103.

Calculated at 340nm	Ratios Marfey of bananamide analogue									
	Ser		Glx		Leu		Thr		Ile	
**Configuration**	L	D	L	D	L	D	L	D-allo	L	D-allo
**Retention Time (min)**	19.989	20.236	21.795	25.252	37.698	42.457	22.454	23.283	36.819	41.903
**Height**	/	153.123	1.71	89.959	302.426	396.134	7.34	144.547	199.765	4.169
**Ratio**	0	1	0	1	2	2	0	1	1	0

The Marfey analysis yielded essential information regarding the topology of each individual amino acids, however the presence of D-Leu and L-Leu in a (1:1) ratio impeded to fully assign the absolute configuration of the peptide backbone. It was observed that the discovered peptide consisted out of: D-Ser, D-*allo*-Thr or D-Thr, L-Ile and D-Glu.

Synthesis of different stereochemical possibilities of the SWRI103 CLiP

	**Fatty acid**	**1**	**2**	**3**	**4**	**5**	**6**	**7**	**8**
	3-OH C10	Leu	Glu	Thr	Leu	Leu	Ser	Leu	Ile
**(8:6) 1L (1)**	(R)	L	D	D-allo	D	D	D	L	L
**(8:6) 4L (2)**	(R)	D	D	D-allo	L	D	D	L	L
**(8:6) 5L (3)**	(R)	D	D	D-allo	D	L	D	L	L

The general approach for the synthesis of the three analogues of SWRI103 CLiP (**1**-**3**) with different stereochemistry is based on the previously reported synthesis of pseudodesmin A and almost similar procedures were used throughout. (13-15) A more in-depth discussion about the used design and applicability of the synthetic strategy can be found there. As a similar strategy is used for the synthesis of these three MDN-0066 analogues, its key features are discussed below.

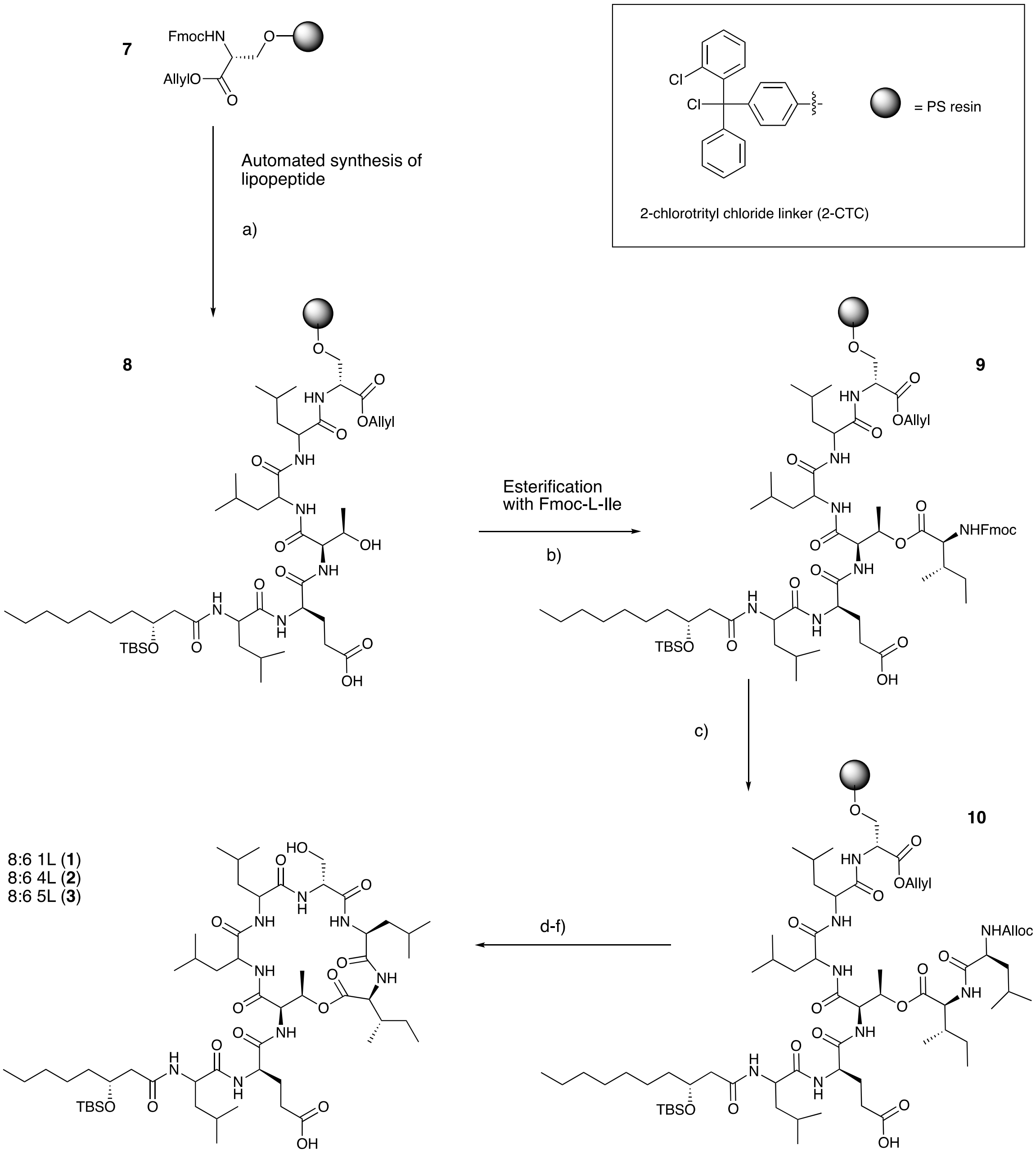

Figure S7: Synthetic route towards synthetic bananamide analogues (1-3). Reagents and conditions: a) (i) 20% piperidine, DMF; (ii) Fmoc-AA-OH, HBTU, DIPEA, DMF; b) Fmoc-L-Ile, DIC, DMAP, DMF; c) (i) 20% piperidine; DMF (ii) Alloc-L-Leu-OH, HBTU, DIPEA, DMF; d) [Pd(PPh_3)$_4$], $PhSiH_3$, CH_2Cl_2; e) HATU, HOBt, DIPEA, DMF; f) 0.1 M HCl in HFIP + 1% TIS.

Synthesis of (8:6) 1L (1)

Anchoring of Fmoc d-Ser(OH)-OAllyl to a 2-Chlorotrityl chloride resin (7)

Fmoc-D-Ser(OH)-OAllyl (1.189 g, 3.24 mmol, 1.5 equiv.) was dissolved in dry THF (13.5ml) in a dry flask under argon atmosphere and pyridine was added (520 µl, 6.48 mmol, 3 equiv.). The 2-CTC resin (1.60 mmol/g; 1.35 g, 2.16 mmol, 1 equiv.) was brought in a dry reaction vessel (100 ml) and then the first reaction mixture was added to the reactor, which was subsequently flushed with argon and sealed. The reactor was placed in a Selecta Vibromatic shaker and connected to a thermostat with a temperature set at 60°C and the reactor was shaken overnight (24h). Next, the excess of reagents was filtered off, the resin was washed with dry DCM and unreacted functionalities were capped by adding a DCM/MeOH/DIPEA (15 ml; 17/2/1; 2×10 min.) solution. After washings with DCM (3x), DMF (3x) and DCM (3x), the beads were dried on the oil pump overnight prior to loading determination. The loading was determined by monitoring the dibenzofulvene-piperidine adduct formed after Fmoc-deprotection and a loading of 0.715 mmol/g was obtained.

Automated solid peptide synthesis towards (8)

Automated solid phase peptide synthesis was done on a Syro apparatus. In a C→N fashion, Fmoc-D-Leu-OH, Fmoc-D-Leu-OH, Fmoc-D-allo-Thr-OH, Fmoc-D-Glu-OH and Fmoc-L-Leu-OH were coupled onto the modified 2-CTC resin (100 mg, 0.715mmole). The automated synthesis included the TBS-protected 3-(R)-hydroxydecanoic acid to cap the N-terminus. After the automated synthesis was completed (12h55min), the resin was washed thoroughly with use of THF (3x), DMF (6x) and DCM (3x). Afterwards, the completion of the reaction was checked by a small cleavage test and the obtained peptide was subjected to LC-MS analysis.

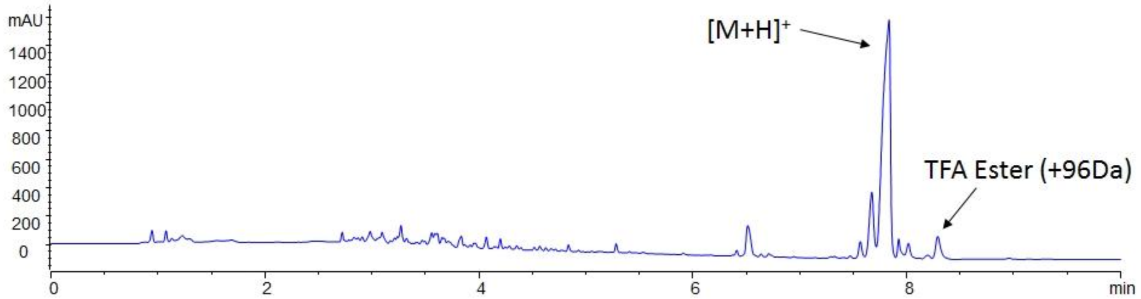

Figure S8: LC-MS analysis of (**8**), gradient used: 0-100% acetonitrile in 6 minutes, 298K; C18-Column. [M+H]+= 884.55 Da in agreement of proposed chemical formula:$C_{42}H_{76}N_6O_{13}$.

Steglich esterification towards (9)

The peptidyl resin was first dried for 2 hours on the oil pump before it was transported to an eppendorf tube (1.5 ml) and stored under argon atmosphere. In a dry flask, Fmoc-L-Ile-OH (227.41 mg; 0.6435 mmol, 10 equiv.) was dissolved in dry THF at 0°C and DIC (90.23 mg, 0.715 mmol, 10 equiv.) was added and, next, this mixture was stirred for 20 minutes at the same temperature. Thereafter, the reaction mixture was added, together with DMAP (1 equiv.) dissolved in dry THF, to the eppendorf tube containing the peptidyl resin. The eppendorf was placed in the thermoshaker and was shaken for 24h at 37°C. Then, the beads were transferred again to the plastic reaction vessel and, subsequently, the resin was washed with THF (3x), DMF (3x) and DCM (3x). A small scale cleavage and LC-MS analysis confirmed ester bond formation.

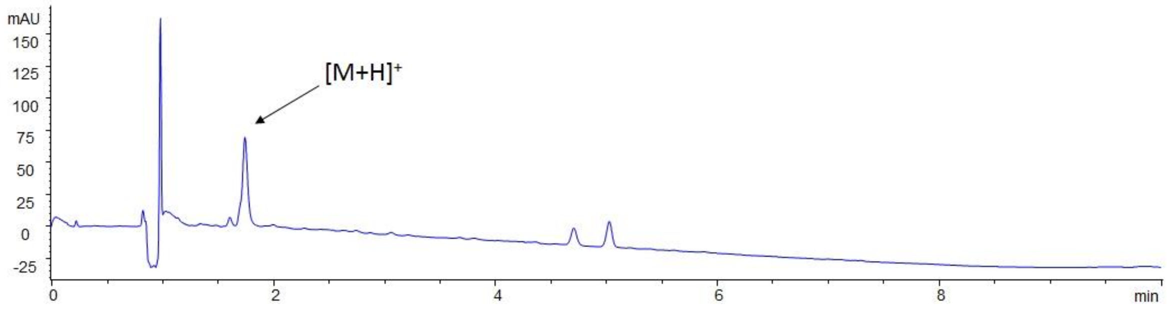

Figure S9: LC-MS analysis of (**9**), gradient used: 75-100% acetonitrile in 6 minutes, 298K; C18-Column. [M+H]+= 997.63 Da in agreement of proposed chemical formula:$C_{49}H_{87}N_7O_{14}$.

Coupling of Alloc-L-Leu-OH towards (10)

A stepwise Fmoc deprotection of (**9**) was performed according to the standard protocol described above. Subsequent, the presence of free amine groups was checked via a standard color test with use of TNBS (2,4,6-trinitrobenzenesulfonic acid). Alloc-L-Leu-OH (77mg; 0.36mmol, 5 equiv.), HBTU (135.59 mg, 0.36mmol, 5 equiv.), DIPEA (100mg, 0.72mmol, 10equiv.) were brought together in a flask and 2ml of DMF was added to dissolve the different compounds. The coupling cocktail was brought to the beats and the mixture was let to shake for 45' at room temperature subsequently, the resin was washed with THF (3x), DMF (3x) and DCM (3x). A small scale cleavage and LC-MS analysis ensured that the coupling was complete.

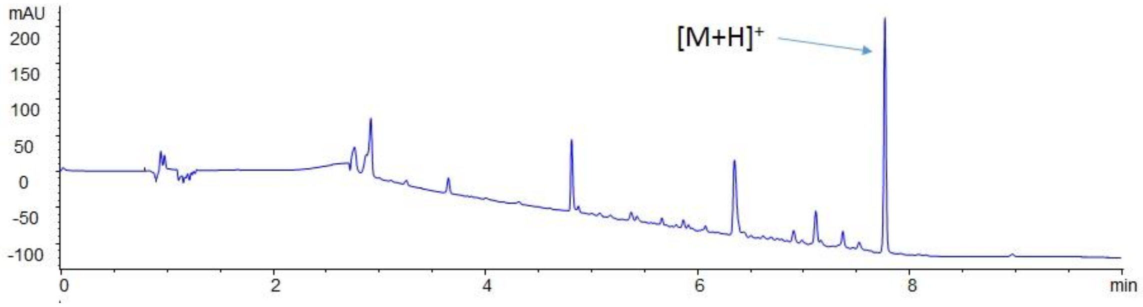

Figure S10: LC-MS analysis of (**10**), gradient used: 0-100% acetonitrile in 6 minutes, 298K; C18-Column. $[M+H]^+$= 1195.74 Da in agreement of proposed chemical formula:$C_{59}H_{102}N_8O_{17}$.

Allyl & alloc deprotection step and on-resin cyclization towards (1)

Under inert conditions (Ar) while shielded off light, tetrakiss ($Pd(PPh_3)_4$) (0.5 equiv., 0,04mmol, 0.042g) was dissolved in a mixture of 1,056ml (60 equiv., 4.29 mmol) $PhSiH_3$ and 4ml dry DMF. The solution was added stepwise to the beads. Firstly, 2ml of the solution was added, letting it react for 1h. Afterwards, the beads were filtered and washed (3x 1ml dry DCM) and another 2ml of the solution was added, repeating the first step. Next, the beads were filtered and the resin was washed thoroughly with 6x DCM, 6x MeOH, 6x DCM and 2x diethyl ether.

In order to perform the on-resin cyclization, HATU (135.92mg, 0.36mmol, 5equiv.) and $HOBt.2H_2O$ (54.74mg, 0.36mg, 5 equiv.) were dissolved in 1,3ml dry DMF. Afterwards, DIPEA (62.25 µL, 0.36 mmol, 5equiv.) was added and the solution was sonicated until fully dissolved. Under inert atmosphere, the solution was transferred to the beads and subsequently the solution, while shielded from incident light, shaken for 4h. The beads were washed 6x DMF, 6x MeOH, 6x DCM

and 2x Et_2O. To ensure full completion, a small test cleavage was performed and the caught peptides were subjected to LC-MS analysis.

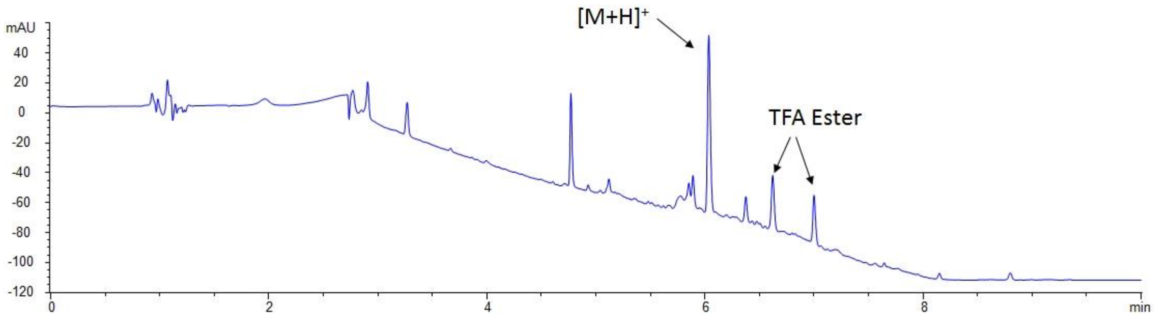

Figure S11: LC-MS analysis of (**1**), gradient used: 0-100% acetonitrile in 6 minutes, 298K; C18-Column. $[M+H]^+$= 1052.68 Da in agreement of proposed chemical formula:$C_{52}H_{92}N_8O_{14}$.

Total cleavage and purification of (1)

Total cleavage from the acid-labile 2-CTC resin was done by adding a 0.1M HCl and a 1%TIS in HFIP solution to the peptide reactor. The solution was shaken for 3h and was afterwards filtered in Falcon Tubes® and was blown dry using a gentle argon flow. Precipitation in cold hexane (x2) was done to remove non-peptidic impurities & protecting groups prior to final purification efforts.

Purification was performed using RP-HPLC (Figure S12). An isothermal gradient of 75%-100% acetonitrile in 15 min was used. All fractions were caught and analyzed. The product was dried overnight at high vacuum. A total of 8mg (11.1% yield, based on resin loading) pure main product was obtained.

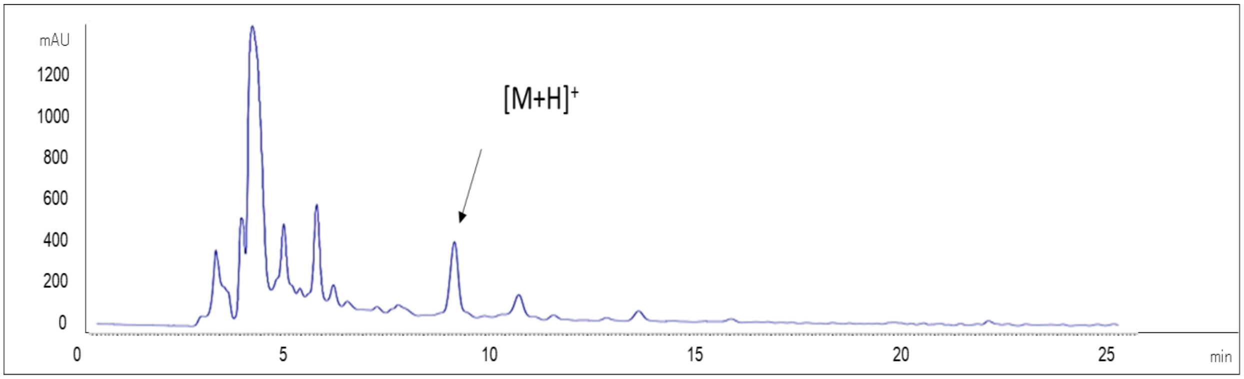

Figure S12: RP-HPLC chromatogram of crude (1). Kinetex C18 column with elution by a linear gradient over 15 min of H_2O (0.1% TFA) and CH_3CN from 25:75 to 0:100. Detection at a wavelength of λ=214 nm.

Synthesis of (8:6) 4L (2)

The synthesis of the (8:6) 4L proceeded quasi identical as the beforementioned method with the only difference occurring at position 1 & 4 where the configurations of the respective amino acids are altered. To avoid repetition, the previous steps are not discussed and only the final chromatogram of the purification efforts are displayed. A total amount of 5.3 mg was obtained (7.04% yield, based on the initial resin loading).

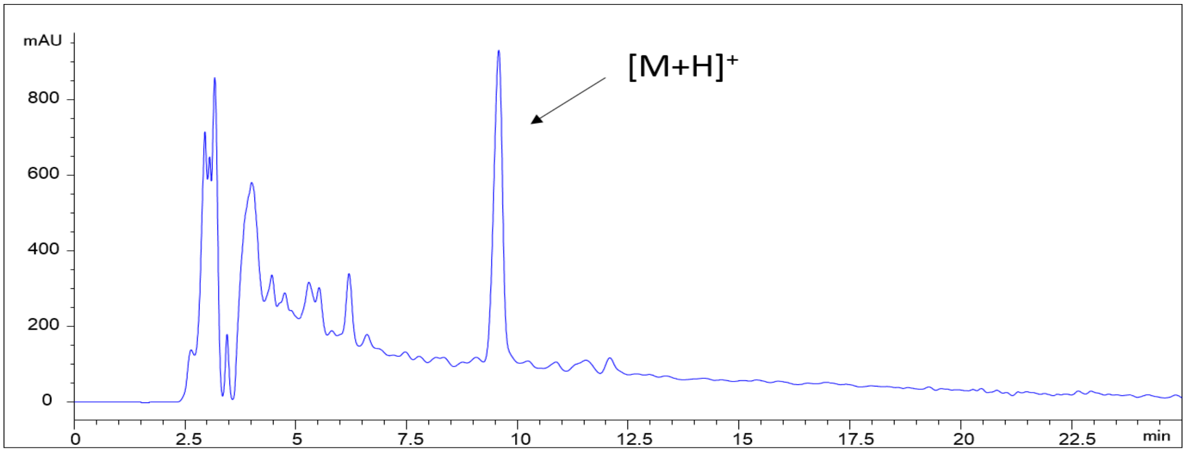

Figure S13: RP-HPLC chromatogram of crude (2). Kinetex C18 column with elution by a linear gradient over 15 min of H_2O (0.1% TFA) and CH_3CN from 25:75 to 0:100. Detection at a wavelength of λ=214 nm

Synthesis of (8:6) 5L (3)

The synthesis of (8:6)-5L variant proceeded very similar as previous analogues. The same peptidyl resin & quantity was used throughout. To avoid repetition, only the conclusive LC-MS analysis of the final purification HPLC chromatogram (Figure S14) is depicted. A quantity of 6 mg of final peptide was achieved, (7,97% yield, based on initial resin loading).

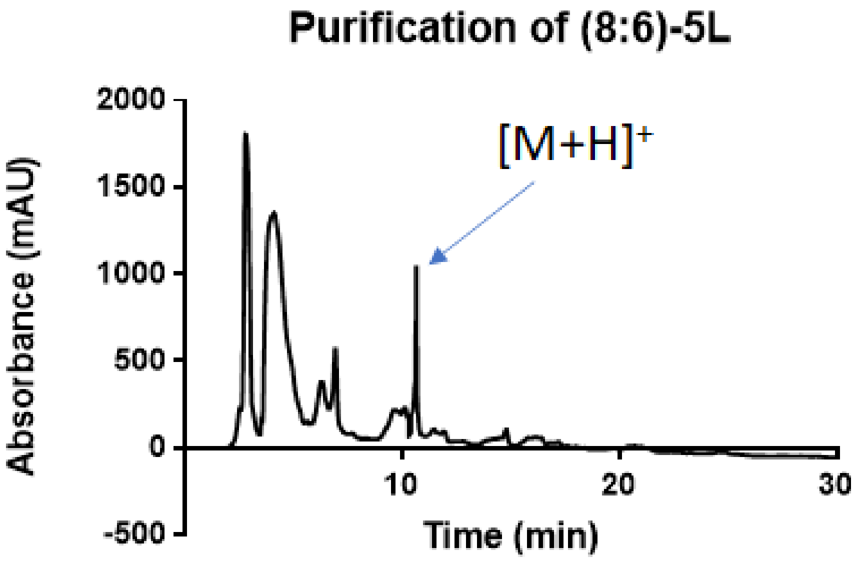

Figure S14: RP-HPLC of (8:6)-5L (**3**): Kinetex C18 column with elution by a linear gradient over 15 min of H_2O (0.1% TFA) and CH_3CN from 25:75 to 0:100. Detection at a wavelength of λ=214 nm.

NMR characterization of cyclic lipodepsipeptides 1-3.

NMR characterization of cyclic lipodepsipeptide (1)

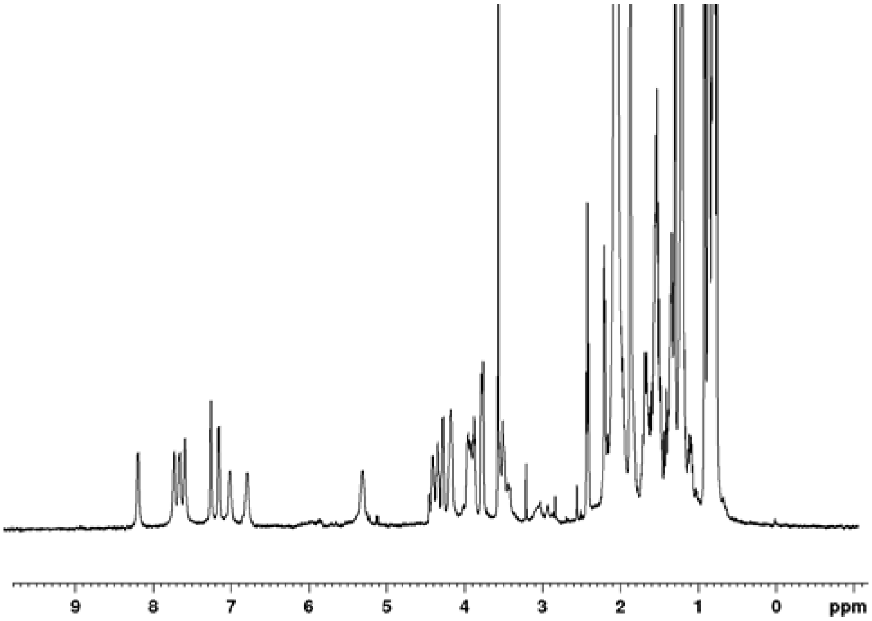

Figure S15: 1D ^1H spectrum of the synthesis product of (8:6) 1L (1). (CD_3CN, 303K, 700 MHz).

Table S5: NMR assignment (^{1}H and ^{13}C) of the synthetic bananamide-analogue (8:6)-L1 **(1)** (500MHz, acetonitrile-d3, 303K)

**(8:6)-L1**		**1H δ [ppm]**	**13C δ [ppm]**			**1H δ [ppm]**	**13C δ [ppm]**
**L-Leu1**				**D-Leu5**			
^{3}JHNHα	NH	6.87		^{3}JHNHα	NH	7.33	
= n.d	CHα	4.26	53.70	= n.d	CHα	3.99	54.51
	CH2β1	1.75	39.92		CH2β1	1.59	41.00
	CH2β2	1.62	39.92		CH2β2	1.59	41.00
	CHγ	1.72	23.32		CHγ	1.73	25.75
	CH3δ	0.87	15.32		CH3δ	/	
	CH3δ	0.87	15.32		CH3δ	/	
	CO	/	n.d		CO	/	n.d
**D-Glu2**				**D-Ser6**			
^{3}JHNHα	NH	7.80		^{3}JHNHα	NH	7.23	
=n.d	CHα	4.03	56.27	=n.d	CHα	4.34	56.93
	CH2β1	2.06	26.25		CH2β1	3.84	63.59
	CH2β2	2.06	26.25		CH2β2	3.57	63.74
	CH2γ1	2.50	30.94		OH	/	
	CH2γ2	2.50	30.94		CO		n.d
	OH						
	CO		n.d				
**D-aThr3**				**L-Leu7**	NH	7.09	
^{3}JHNHα	NH	8.27		^{3}JHNHα	CHα	4.40	53.95
=n.d	CHα	4.24	59.71	=n.d	CH2β1	1.62	40.7
	CHβ	5.38	69.37		CH2β2	1.62	40.7
	CH3γ1	1.35	18.85		CHγ	1.72	25.22
	CH3γ2	1.35	18.85		CH3δ	0.91	21.34
	CO		n.d		CH3δ	0.91	21.34
**D-Leu4**	NH	7.65			CO		n.d
^{3}JHNHα	CHα	3.85	56.02	**L-Ile8**	NH		
= n.d	CH2β1	1.51	41.80	^{3}JHNHα	CHα	4.47	57.03
	CH2β2	1.51	41.80	=n.d	CH β	1.91	37.45
	CHγ	1.73	25.32		CH3γ	0.84	21.44
	CH3δ	0.85	15.19		CH2 γ	1.16	25.71
	CH3δ	0.85	15.19		CH2 γ	1.16	25.71
	CO		n.d		CH3 δ	0.87	11.52
					CO		n.d
**FA**							
	CO		n.d				
	C-2'	2.27	44.01				
	C-3'	3.98	69.30				
	OH	/					
	C-5'	1.49	38.22				
	C-6'	1.42	26.30				
	C-7'	1.33	26.30				
	C-8'	1.28	32.50				
	C-9'	1.27	32.60				
	C-10'	0.88	14.36				

NMR characterization of cyclic lipodepsipeptide (2)

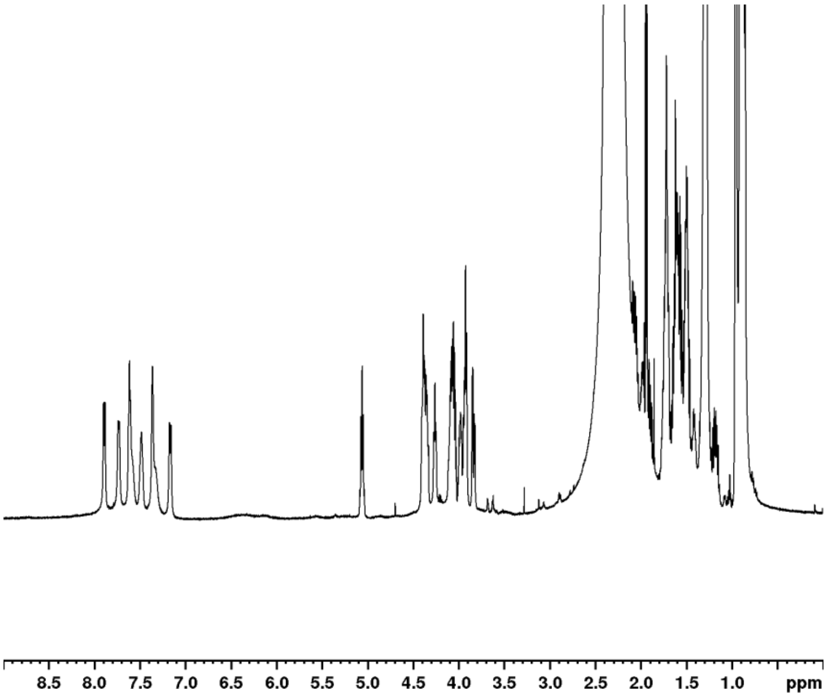

Figure S16: 1D ^{1}H spectrum of the synthesis product of (8:6) 4L (2). (CD_3CN, 303K , 700 MHz).

Table S6: NMR assignment (^{1}H and ^{13}C) of the synthetic bananamide-analogue (8:6)-L4 **(2)** (500MHz, acetonitrile-d3, 303K)

		**1H δ [ppm]**	**13C δ [ppm]**			**1H δ [ppm]**	**13C δ [ppm]**
**D-Leu1**				**D-Leu5**			
^{3}JHNHα	NH	7.57		**^{3}JHNHα**	NH	7.42	
= 6.56Hz	CHα	4.02	55.42	=6.16Hz	CHα	4.05	54.35
	CH2β1	1.54	40.30		CH2β1	1.72	40.07
	CH2β2	1.61	40.30		CH2β2	1.72	40.07
	CHγ	1.74	25.43		CHγ	1.72	25.26
	CH3δ	0.90	22.99		CH3δ	0.89	20.72
	CH3δ	0.96	22.98		CH3δ	0.96	?
	CO	/	174.50		CO	/	173.20
**D-Glu2**				**D-Ser6**			
^{3}JHNHα	NH	7.59		**^{3}JHNHα**	NH	7.92	/
=6.26Hz	CHα	4.05	55.41	=8.24Hz	CHα	4.34	57.84
	CH2β1	2.06	26.21		CH2β1	3.94	62.30
	CH2β2	1.91	26.24		CH2β2	3.84	62.30
	CH2γ1	2.39	31.06		OH		
	CH2γ2	2.39	31.06		CO		170.10
	OH						
	CO		172.70				
**D-aThr3**				**L-Leu7**	NH	7.29	
^{3}JHNHα	NH	7.72		**^{3}JHNHα**	CHα	4.38	53.85
=7.40Hz	CHα	4.22	59.83	=8.98Hz	CH2β1	1.55	43.85
	CHβ	5.06	71.46		CH2β2	1.49	43.74
	CH3γ1	1.27	18.49		CHγ	1.61	25.10
	CH3γ2	1.27	18.84		CH3δ	0.90	21.67
	CO		171.40		CH3δ	0.85	21.59
**L-Leu4**	NH	7.34			CO		170.11
^{3}JHNHα	CHα	3.91	55.05	**L-Ile8**	NH	7.15	
= n.d	CH2β1	1.72	39.99	**^{3}JHNHα**	CHα	4.40	57.95
	CH2β2	1.62	40.39	=8.91Hz	CH β	1.98	37.51
	CHγ	1.74	25.48		CH3γ	0.90	15.30
	CH3δ	0.90	22.99		CH2 γ	1.48	25.30
	CH3δ	0.96	22.98		CH2 γ	1.51	25.30
	CO		174.35		CH3 δ	0.80	11.51
					CO		172.12
**FA**							
	CO		174.90				
	C-2'	2.37	44.01				
	C-3'	3.98	70.27				
	OH	/					
	C-5'	1.49	38.22				
	C-6'	1.42	26.30				
	C-7'	1.33	26.30				
	C-8'	1.28	32.50				
	C-9'	1.27	32.60				

NMR characterization of cyclic lipodepsipeptide (3):

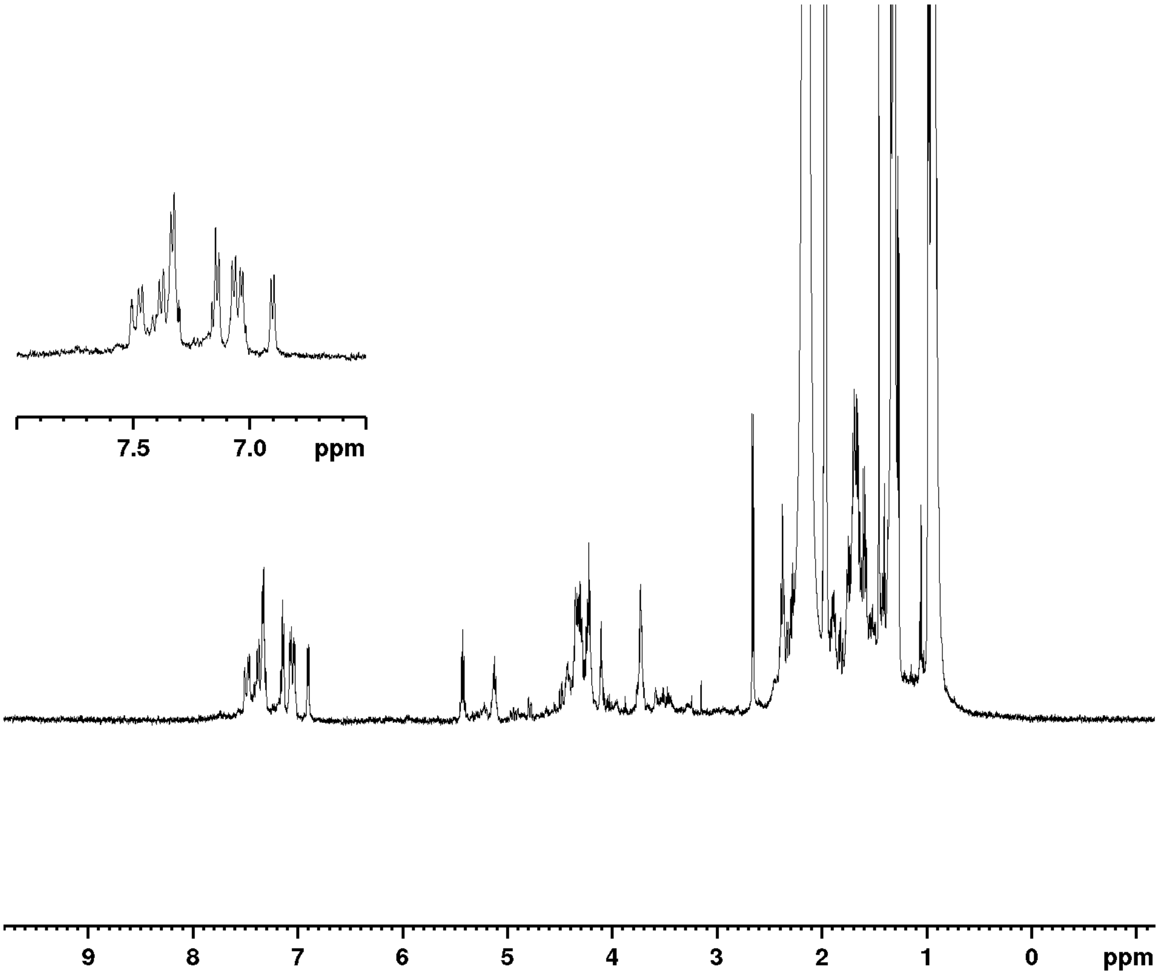

Figure S17: 1D ^{1}H spectrum of the synthesis product of (8:6) 5L (3). (CD_3CN, 303K , 700 MHz). The product suffered from limited solubility in acetonitrile.

Table S7: NMR assignment (^{1}H and ^{13}C) of the synthetic bananamide-analogue (8:6)-L5 **(3)** (500MHz, acetonitrile-d3, 303K)

**(8:6)-L5**		**1H δ [ppm]**	**13C δ [ppm]**			**1H δ [ppm]**	**13C δ [ppm]**
**L-Leu1**				**D-Leu5**			
	NH	6.87			NH	7.31	
	CHα	4.21	53.51		CHα	4.32	52.48
	CH2β1	1.57	41.11		CH2β1	1.67	39.41
	CH2β2	1.59	41.11		CH2β2	1.67	39.41
	CHγ	1.67	25.50		CHγ	1.34	30.60
	CH3δ	0.91	21.79		CH3δ	0.92	21.80
	CH3δ	0.91	21.79		CH3δ	0.92	21.80
	CO		/		CO		
**D-Glu2**				**D-Ser6**			
	NH	7.11			NH	6.87	
	CHα	4.27	53.72		CHα	4.20	60.10
	CH2β1	1.86	27.08		CH2β1	3.71	62.45
	CH2β2	2.08	27.08		CH2β2	3.72	62.45
	CH2γ1	2.35	30.74		OH	n.a	
	CH2γ2	2.35	30.74		CO		/
	OH	/					
	CO	n.a					
**D-aThr3**				**L-Leu7**	NH	7.45	
	NH	7.30			CHα	4.39	52.36
	CHα	4.32	56.00		CH2β1	1.58	41.73
	CHβ	5.10			CH2β2	1.61	41.73
	CH3γ1	0.91	15.67		CHγ	n.a	
	CH3γ2	0.91	15.67		CH3δ	0.93	23.25
	CO	n.a			CH3δ	0.93	23.25
**D-Leu4**	NH	n.a			CO	n.a	
	CHα	7.05		**L-Ile8**	NH	7.02	
	CH2β1	4.29	52.81		CHα	4.07	58.80
	CH2β2	1.64	39.54		CH β	1.67	25.45
	CHγ	1.62	39.54		CH3γ	1.27	17.80
	CH3δ	n.a			CH2 γ	1.31	25.53
	CH3δ	0.94	24.01		CH2 γ	1.31	25.53
	CO	0.94	24.01		CH3 δ	0.87	12.10
					CO	n.a	
**FA**							
	CO						
	C-2'	n.a					
	C-3'	2.65	40.58				
	OH	5.40	70.78				
	C-5'	/					
	C-6'	n.a					
	C-7'	n.a					
	C-8'	n.a					
	C-9'	n.a					
	C-10'	n.a					

NMR characterization of cyclic lipodepsipeptide 3S-OH C10:0 MDN0066 (compound 2b):

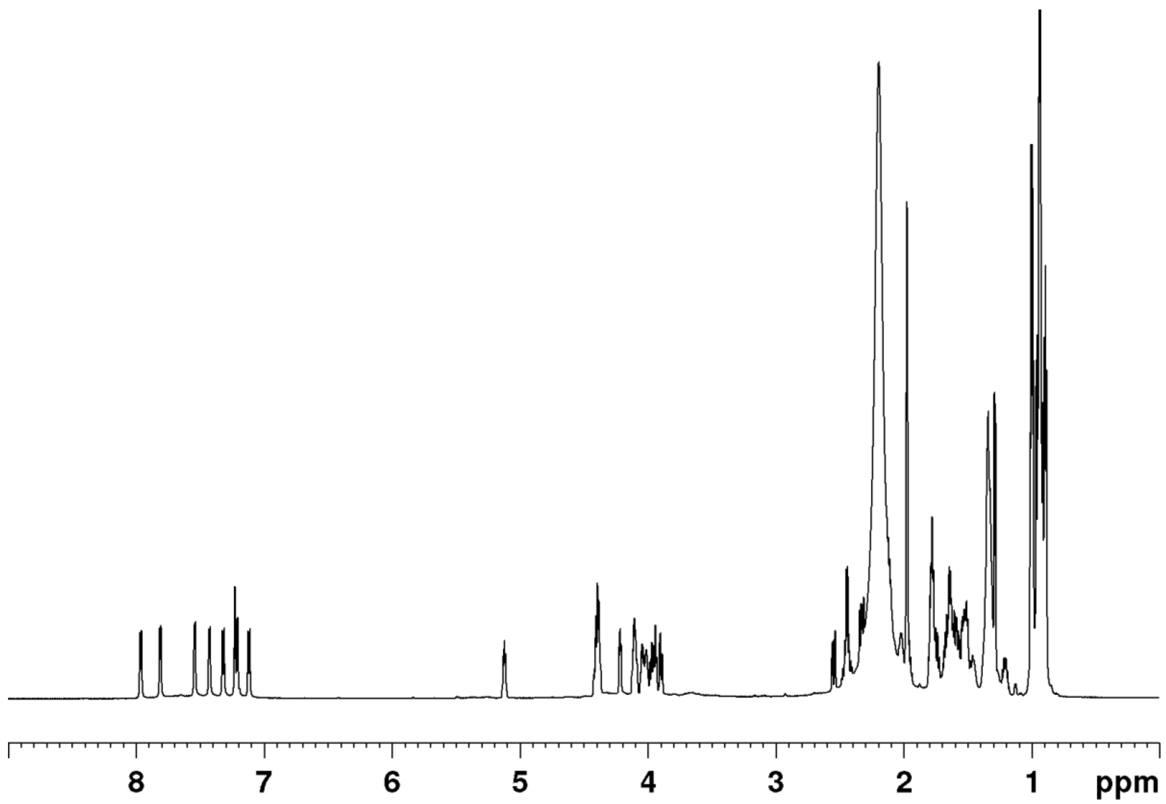

Figure S18: 1D ^{1}H spectrum of the synthesis product of (8:6) 4L with a 3S-OH C10:0 fatty acid tail (**2b**). (CD_3CN, 303K , 500 MHz).

Table S8: NMR assignment (^{1}H and ^{13}C) of the synthetic bananamide-analogue (8:6)-L4 with a 3S-OH C10:0 fatty acid tail **(2b)** (500MHz, acetonitrile-d3, 303K)

		1H δ [ppm]	13C δ [ppm]			1H δ [ppm]	13C δ [ppm]
**D-Leu1**				**D -Leu5**			
	NH	7.17	-	**^{3}JHNHα**	NH	7.39	-
	CHα	4.01	55.2	=6.12Hz	CHα	4.06	54.5
	CH2β	1.75	40.0		CH2β	1.70	40.0
	CHγ	1.64	25.2		CHγ	1.75	25.5
	CH3δ	0.96	23.0		CH3δ	0.97	23.4
	CH3δ	0.90	20.8		CH3δ	0.90	22.9
	CO	-	175.3		CO	-	173.9
**D -Glu2**				**D -Ser6**			
**^{3}JHNHα**	NH	7.51	-	**^{3}JHNHα**	NH	7.93	-
**=5.62Hz**	CHα	4.07	55.2	=8.26Hz	CHα	4.34	57.9
	CH2β1	2.08	26.1		CH2β1	3.94	62.2
	CH2β2	1.92	26.1		CH2β2	3.86	62.2
	CH2γ	2.41	30.8		OH	n.d.	-
	OH	n.d.	-		CO	-	171.1
	CO	-	173.3				
**D -aThr3**							
**^{3}JHNHα**	NH	7.78	-	**L -Leu7**	NH	7.29	-
**=6.78Hz**	CHα	4.18	60.0	**^{3}JHNHα**	CHα	4.37	53.9
	CHβ	5.09	71.6	=9.42Hz	CH2β	1.58	40.8
	CH3γ	1.25	18.9		CHγ	1.73	25.4
	CO	-	172.2		CH3δ	0.90	23.3
**L -Leu4**	NH	7.19	-		CH3δ	0.85	21.6
	CHα	3.91	55.2		CO	-	173.1
	CH2β1	1.75	40.7	**L-Ile8**	NH	7.09	-
	CH2β2	1.59	40.7	**^{3}JHNHα**	CHα	4.37	58.2
	CHγ	1.60	25.0	=9.26Hz	CH β	1.98	37.5
	CH3δ	0.96	22.7		CH3γ	0.93	16.0
	CH3δ	0.91	21.8		CH2 γ	1.51	25.8
	CO	-	175.7		CH2 γ	1.17	25.8
					CH3 δ	0.87	11.4
**3S-OH FA**					CO	-	171.0
	CO	-	175.9				
	C-2a'	3.98	69.4				
	C-2b'	2.52	43.7				
	C-3'	2.28	43.7				
	OH	n.d.	-				
	C-5'	1.48	38.0				
	C-6'	1.42	26.3				
	C-6'	1.32	26.3				
	C-7'	1.28	32.6				
	C-8'	1.31	30.1				
	C-9'	1.30	23.3				
	C-10'	0.89	14.3				

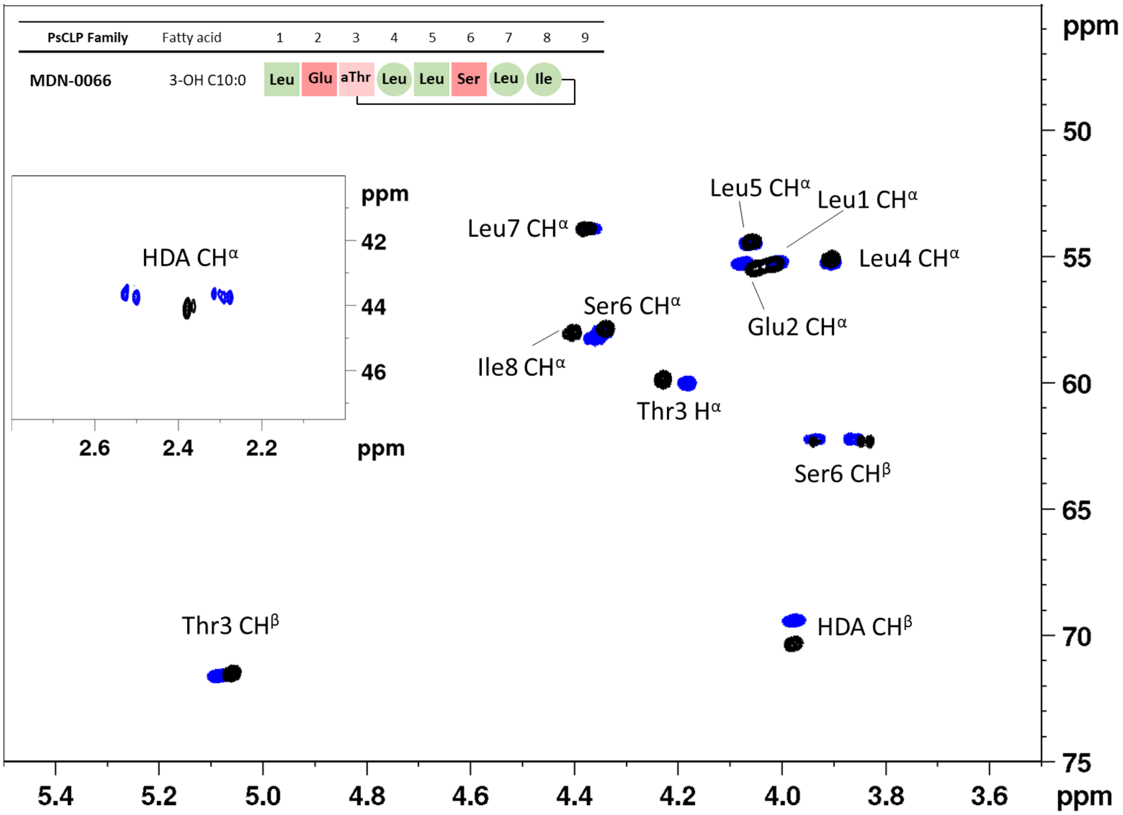

Figure S19: HSQC fingerprint of MDN0066 3-R-OH C10:0 (black, native form), and MDN0066 3-S-OH C10:0 (blue); the inset shows the CH_2^{α} resonances of the fatty acid tail. Differences are notably situated at the N-terminus of the peptide and in the fatty acid tail. (acetonitrile-d3, 298K, 500MHz.)

Analysis of the natural CLiPs from *Pseudomonas aestus* CMR5c

Extraction and purification of orfamide B

Orfamide B was extracted from a batch culture of *P. aestus* CMR5c incubated in King's Broth medium. (Difco Laboratories, Sparks, MD, USA) as before (5).The main compound eluted at a retention time of 13.5 min as shown. This peptide fraction was purified via semi-preparative RP-HPLC analysis, using a two solvent system: H_2O + 01% TFA (A) and CH_3CN (B), via application of a linear gradient over 15 minutes going from 10:90 to 0:100 (A:B)(Figure S20).

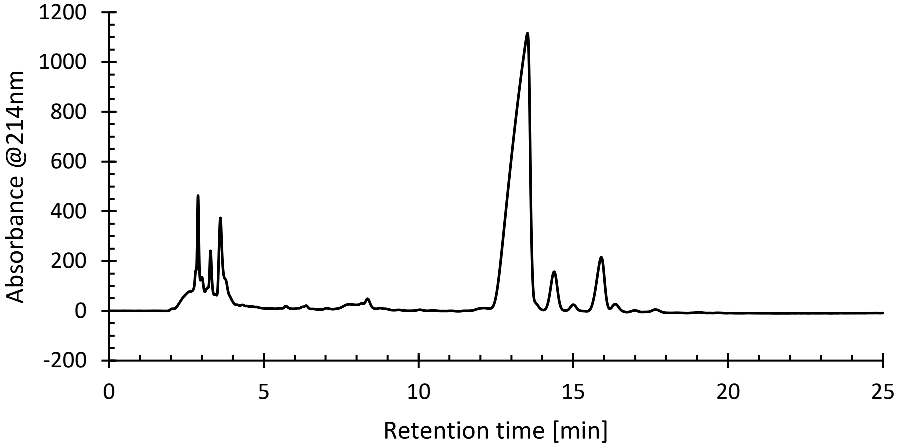

Figure S20: Semi-preparative reversed-phase HPLC chromatogram of *P. aestus* CMR5c, showing the clear presence of orfamide B at 13.5 minutes.

NMR characterization of the natural orfamide B

The molecular structure of the isolated peptide was revealed by solution state NMR. In DMF-d7, full assignment of the ^{1}H and ^{13}C resonances was achieved. The characteristic 2D ^{1}H-^{1}H TOCSY patterns revealed the identity of the amino acids and the 3-hydroxy decanoic acid fatty acid spin system whereas the 2D ^{1}H-^{1}H ROESY spectrum revealed the connectivity of the residues through observation of either H^{N} (i)→ H^{N} (i+1) or H^{N} (i) to H^{α} (i-1) characteristic cross peaks. The carbonyl ^{13}C resonances and the lactone bond between the hydroxyl functionality of Thr3 and Ile8 could be established by analyzing the ^{1}H-{^{13}C} gHMBC.

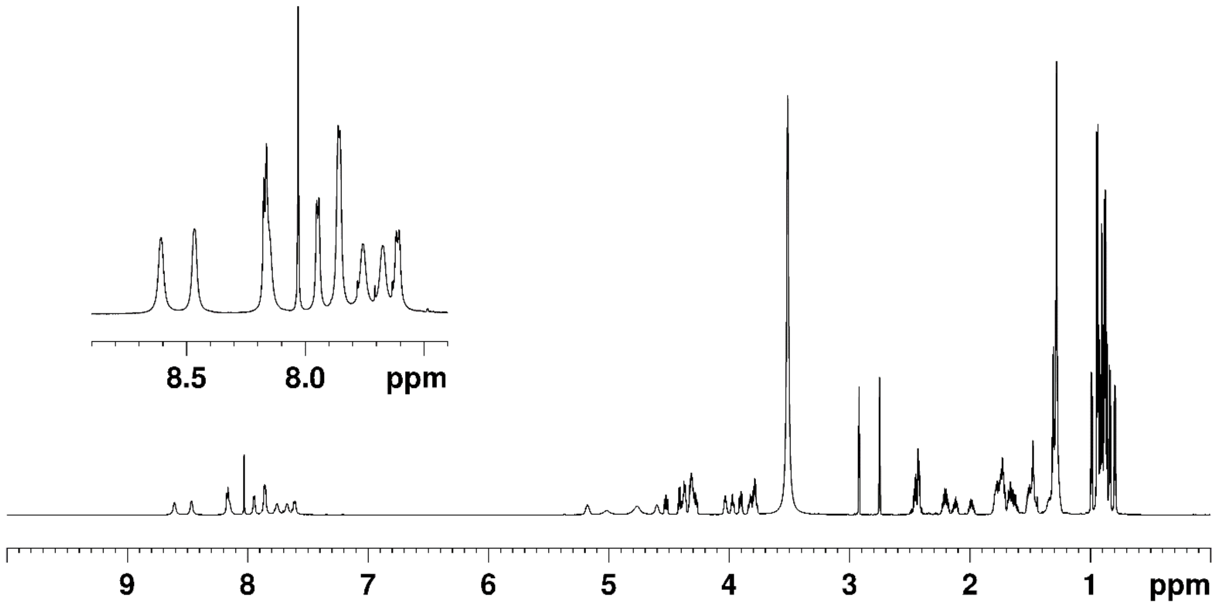

Figure S21: 1D ^{1}H spectrum of the purified orfamide B as extracted from *P. aestus* CMR5c. (DMF-d7, 298K , 500 MHz)

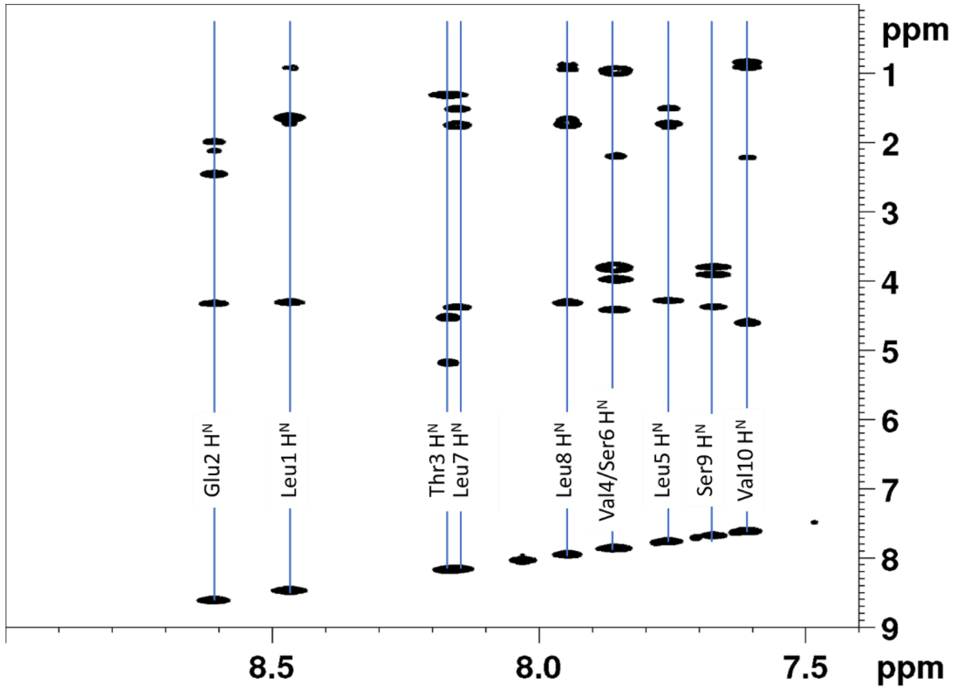

Figure S22: ^{1}H-^{1}H TOCSY traces of the individual amino acids in orfamide B as isolated from *P. aestus* CMR5c.(500MHz, DMF-*d7*, 298K)

Table S9: NMR assignment (^1H and ^{13}C) of orfamide B, extracted as a major compound produced by *P. aestus* CMR5c. (500MHz, DMF-d7, 298K)

		**^1H δ [ppm]**	**^{13}C δ [ppm]**			**^1H δ [ppm]**	**^{13}C δ [ppm]**
**FA**				D-**Leu5**			
	CO	-	172.96		NH	7.76	-
	CH_2α	2.43	43.73		CHα	4.275	53.13
	CHβ	4.03	68.38		CO	-	n.d.
	CH_2γ	1.48	37.63		CH_2β1	1.73	40.05
	CH_2δ1	1.48	25.67		CH_2β2	1.50	40.00
	CH_2δ2	1.39	25.67		CHγ	1.79	24.44
	CH_2ε	1.28	29.55		CH_3δ1	0.88	23.01
	CH_2ζ	1.28	29.55		CH_3δ2	0.80	20.67
	CH_2η	1.28	29.55	D-**Ser6**			
	CH_2θ	1.28	29.55		NH	7.86	-
	CH_2ι	1.28	29.55		CHα	4.41	56.96
	CH_2κ	1.28	29.55		CO	-	n.d.
	CH_2λ	1.27	31.90		CH_2β1	3.83	62.09
	CH2μ	1.29	22.53		CH_2β2	3.78	62.09
	CH_3ν	0.88	13.71		OHγ	4.76	-
	OH	n.d.	-	L-**Leu7**			
L-**Leu1**					NH	8.15	-
	NH	8.47	-		CHα	4.38	52.76
	CHα	4.30	52.44		CO	-	173.23
	CO	-	n.d.		CH_2β1	1.73	40.77
	CH_2β1	1.66	40.20		CH_2β2	1.51	40.77
	CH_2β2	1.63	40.20		CHγ	1.78	24.49
	CHγ	1.73	24.59		CH_3δ1	0.93	22.89
	CH_3δ1	0.94	22.68		CH_3δ2	0.87	21.05
	CH_3δ2	0.90	21.49	L-**Leu8**			
D-**Glu2**					NH	7.95	-
	NH	8.61	-		CHα	4.31	52.77
	CHα	4.32	54.08		CO	-	n.d.
	CO	-	173.40		CH_2β1	1.73	40.34
	CH_2β1	2.12	26.60		CH_2β2	1.68	40.33
	CH_2β2	1.98	26.60		CHγ	1.75	24.59
	CH_2γ	2.45	30.12		CH_3δ1	0.94	22.68
	COδ	-	173.97		CH_3δ2	0.88	21.14
	OH	n.d.	-	D-**Ser9**			
D-**aThr3**					NH	7.67	-
	NH	8.17	-		CHα	4.37	56.27
	CHα	4.53	58.40		CO	-	170.71
	CO	-	n.d.		CH_2β1	3.91	62.23
	CHβ	5.17	70.12		CH_2β2	3.79	62.23
	CH_3γ	1.31	17.47		OHγ	5.01	-
D-**Val4**				L-**Val10**			
	NH	7.86	-		NH	7.61	-
	CHα	3.97	60.82		CHα	4.60	57.70
	CO	-	n.d.		CO	-	n.d.
	CHβ	2.19	29.69		CHβ	2.21	30.38
	CH_3γ1	0.99	19.00		CH_2γ1	0.91	18.92
	CH_3γ2	0.94	19.01		CH_2γ2	0.84	17.61

Stereochemical analysis of the natural CLiPs from *Pseudomonas aestus* CMR5c

In a 10ml round bottom flask, 0.63 mg orfamide B was dissolved in 1ml 6N aq.HCl and was let to stir for 24h at 100°C.The hydrolysate was dried using a gentle argon flow while simultaneously heating to 60°C. 300 µL of a 1M $NaHCO_3$ solution was added to the residue. White precipitation could be noticed.. Subsequently, 100 µL of a 1%-FDAA in acetone solution was added and it was let to react for 1h at 50°C. A color shift (bright yellow to copper-like orange) could be noticed. The reaction was quenched with 20µL 2M aq.HCl and was dried in vacuo. Afterwards, the dried compounds were dissolved in 200µL DMSO of which 20µL was taken out and was diluted to 200 µL with use of a 60:40 ammonium acetate (5mM): acetonitrile buffer prior to injection. Reversed-phase HPLC analysis of the derivatized hydrolysate was performed on an Agilent Technologies 1260 Infinity II instrument with a Kinetex RP-18 (C18, 150 × 4.60 mm, 5 µm particle size) column and connected to a multiwavelength detector. The solvents used for the measure of samples are 0.1% TFA in H_2O (A) and MeCN (B) at a flow rate of 3 mL min^{-1}. After sample injection, the column was flushed with 100% A for 3 min, followed by an isothermal (50°C) gradient from 0 to 100 % B over 120 min and subsequent rinsing with 100 % B for 3 min. The detector was set to 340 nm to selectively monitor the derivatives.

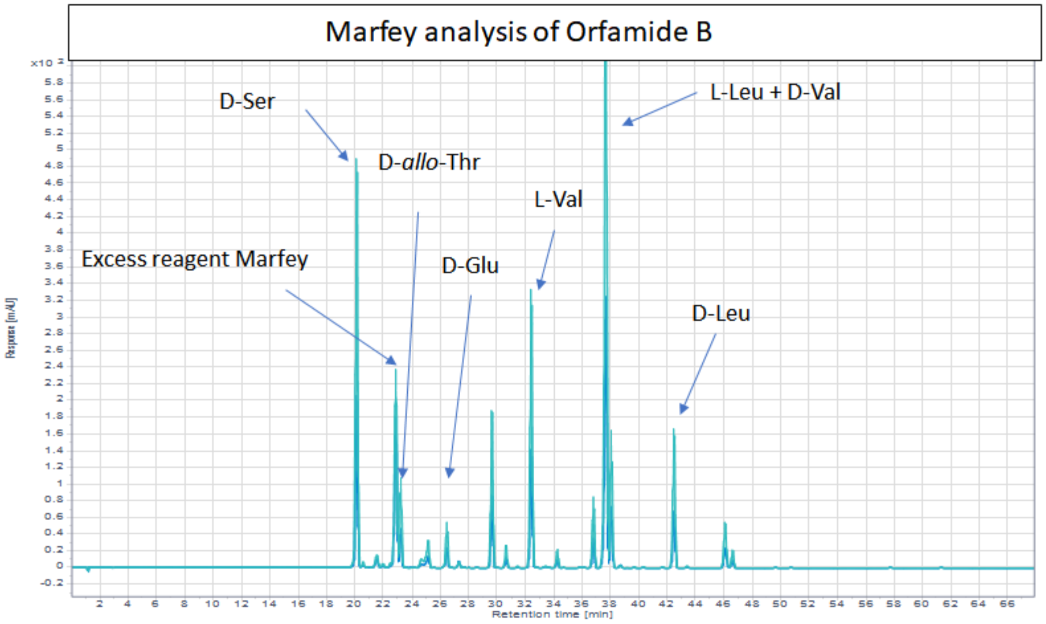

Figure S23: Chromatogram obtained by derivatization of the individual amino acid present in orfamide B. Conditions are highlighted above.

Table S10: Summary of the found A.A. enantiomeric ratios from orfamide B.

	Ratios Marfey of orfamide B analogue									
Calculated at 340 nm	Ser		Glu		Val		Leu		Thr	
Configuration	L	D	L	D	L	D	L	D	L	D-allo
Retention Time (min)	19.989	20.063	21.795	26.424	32.785	37.626	37.626	42.434	22.454	22.824
Height	/	490.35	1.84	54.95	334.58	792.124	792.124	167.25	7.34	234.79
Ratio	0	2	0	1	1	1	3	1	0	1

Synthesis of different stereochemical possibilities of CMR5c-orfamide.

The general approach for the synthesis of the synthetic analogues of orfamide (**4-6**) with different stereochemistry for the leucine residue at position 1 and 5 in the peptide sequence is based on the previously reported synthesis of pseudodesmin A and almost identical procedures were used throughout (13-15). A more detailed discussion about the design and development of the synthesis strategy can be found there. The key features of this similar strategy used for the synthesis of these three analogues, are discussed below.

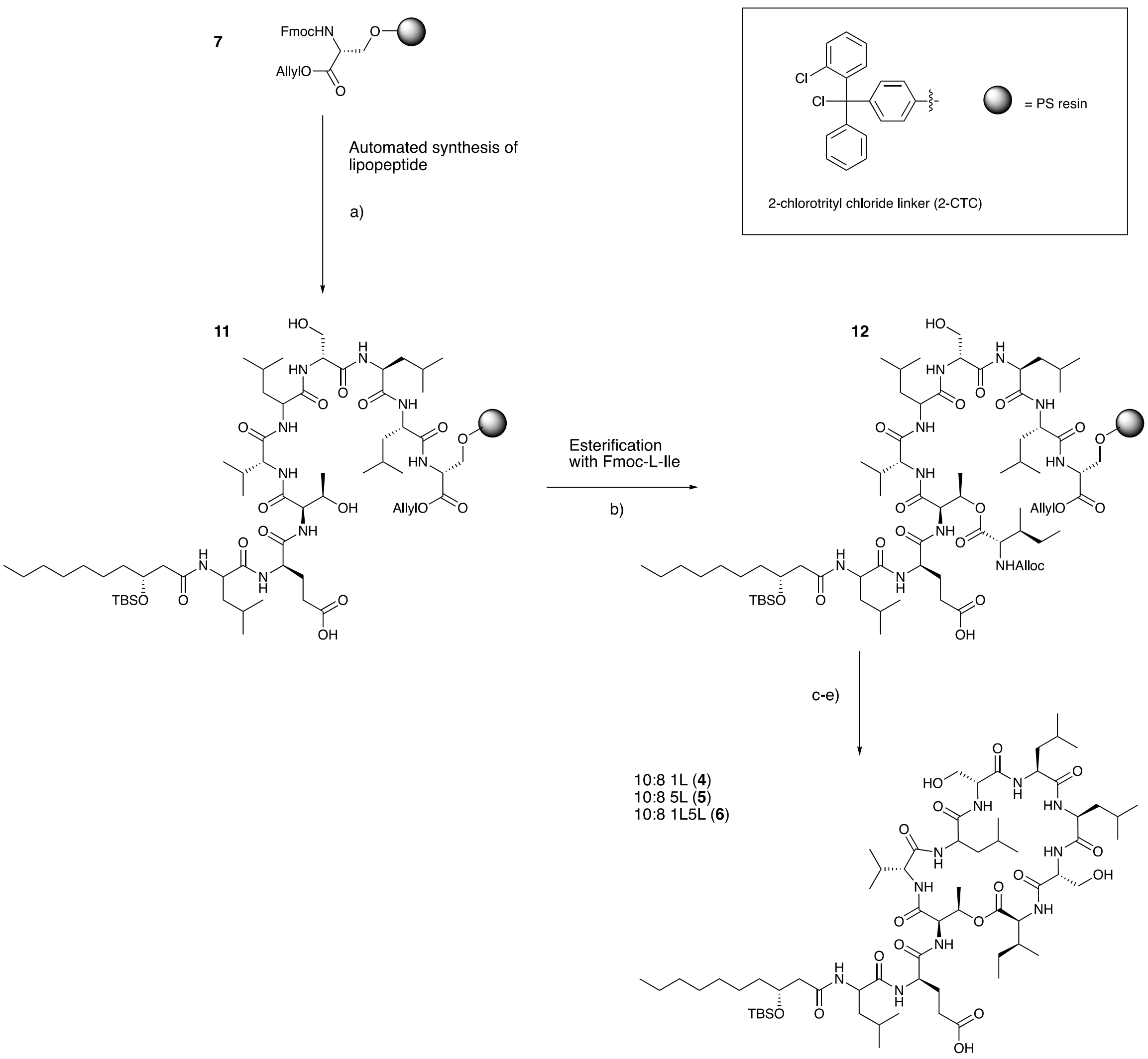

Figure S24: Synthetic route towards synthetic orfamide analogues (**4**-**6**). Reagents and conditions: a) (i) 20% piperidine, DMF; (ii) Fmoc-AA-OH, HBTU, DIPEA, DMF; b) Fmoc-L-Ile, DIC, DMAP, DMF; c) [Pd(PPh_3)$_4$], $PhSiH_3$, CH_2Cl_2; d) HATU, HOBt, DIPEA, DMF; e) 0.1 M HCl in HFIP + 1% TIS.

Synthesis of (10:8) 1L (4)

Automated solid peptide synthesis towards (11):

The synthesis of the (10:8) 1L which has the L-leu at position 1 and an D-Leu at position 5 was started from the same preloaded resin (**7**) as used for the synthesis of the bananamide analogues (0.358 mmol/g; 200 mg; 0.0716 mmol). The attachment of the 8 amino acid residues: Fmoc-L-Leu-OH, Fmoc-L-Leu-OH, Fmoc-D-Ser(tBu)-OH, Fmoc-L-Leu-OH, Fmoc-D-Val-OH, Fmoc-D-allo-Thr-OH, Fmoc-D-Glu(tBu)-OH, Fmoc-D-Leu-OH, was proceeded in an automated fashion consisting of repetitive cycles of Fmoc-deprotection and coupling the next amino acid. To lower the consumption of the lipid tail, (*R*) 3-hydroxy decanoic acid, manual coupling was preferred. Note that the main product of CMR5c has a (*R*) 3-hydroxy tetradecanoic acid as fatty acid moiety. Due to the exceptionally high price of the commercially available (*R*) 3-hydroxy tetradecanoic acid and the in house availability of (*R*) 3-hydroxy decanoic acid, we chose to couple this C10 variant. In addition, the usage of another lipid tail analogue does not interfere with our intended purpose as we previously demonstrated that alterations in the length of the lipid tail do not affect the proton-carbon correlation in the characteristic fingerprint CH^{α} region (5).

The success of this elongation of the peptide chain was assessed via a small scale cleavage and of the peptidyl resin (1 mg) and subsequent LC-MS analysis of the obtained peptide fraction.

LC-MS analysis

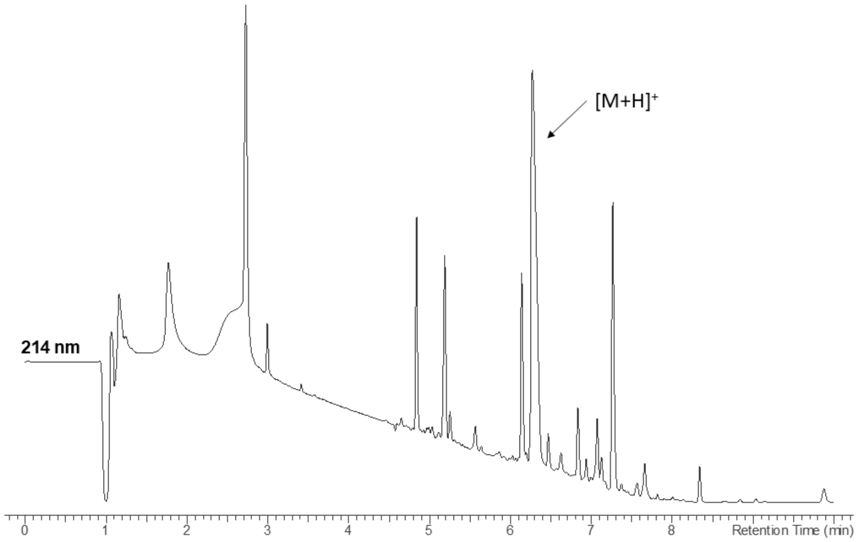

Figure S25: LC-MS analysis of **11** (Figure S21). The fatty acid tail was coupled manually. $[M+H]^+$ was 1183.73 Da which was in agreement with $C_{57}H_{101}N_9O_1$.

Steglich esterification towards (12)

The formation of the esterbond was performed using Alloc-L-Ile as a building block. First, the peptidyl resin was dried via several washing steps with Et_2O (3x) and further dried at the oil pump when necessary. Thereafter, the resin beads were transferred to an Eppendorf tube (1.5 ml) and stored under argon atmosphere. In a dry flask, Alloc-L-Ile-OH (0.154 g; 0.716 mmol, 10 equiv.) was dissolved in dry THF at 0°C and DIC (99.5 mg, 0.788 mmol, 11 equiv.) was added. The mixture was stirred for 20 minutes at the 0°C. DMAP (1 equiv.) dissolved in dry THF. Both solutions were added to the Eppendorf tube containing the peptidyl resin. The Eppendorf was sealed with parafilm, placed in a thermoshaker and shaken for 24h at 37°C. Then, the beads were transferred back to the plastic reaction vessel and, subsequently, the resin was washed with THF (3x), DMF (6x), DCM (6x) and again DMF (6x). A small scale cleavage and LC-MS analysis confirmed ester bond formation.

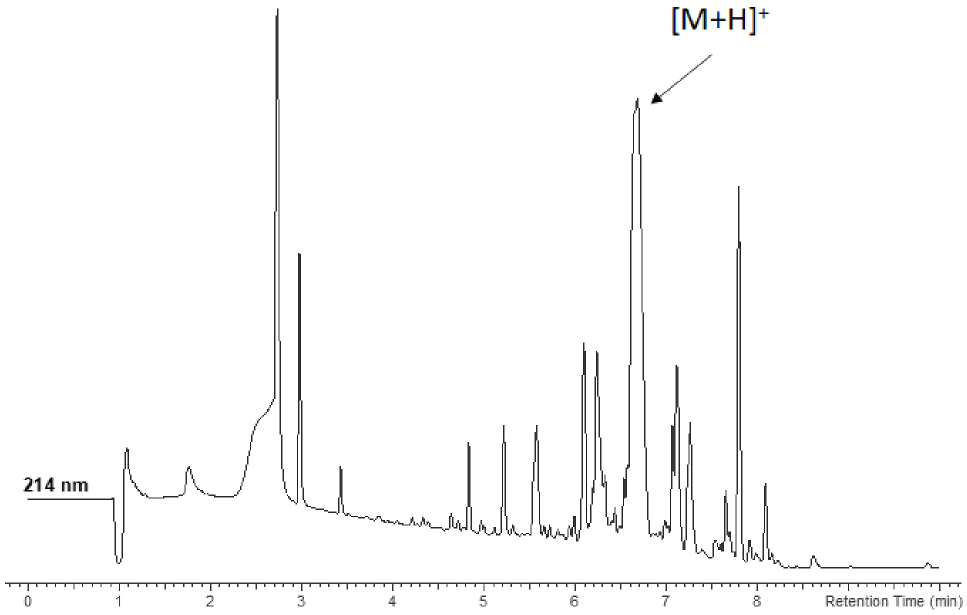

Figure S26: LC-MS analysis of **12** (Figure S21). $[M+H]^+$ was 1326.8 Da which was in agreement with $C_{63}H_{110}N_{10}O_{20}$.

Allyl/Alloc deprotection and on-resin cyclization (4)

The dry resin beads were first washed with dry DCM under inert conditions and after 2 mL of dry DCM was added to the reaction vessel. Phenylsilane (4.29 mmol, 60 equiv.) and a catalytic amount of $Pd(PPh_3)_4$ (0.018 mmol, 0.25 equiv.) were also added. Next, the mixture with the peptidyl resin was flushed with Argon and shielded from light. After the reaction vessel was shaken for 1 hour, the solution was filtered, the resin was washed with dry DCM (3x) and the deprotection step, described above, was repeated once more. Thereafter, the resin wash thoroughly washed with DMF (6x), DCM (6x), MeOH (3x) and DMF (6x) and dried by washing with Et_2O (3x).

For the on-resin cyclization, HATU (5 equiv.) and HOBt (5 equiv.) were added together and dissolved in 2 mL dry DMF. After DIPEA (5 equiv.) was added to the solution, the solution was sonicated and added to the resin under argon atmosphere. The reaction vessel was shaken for 4 hours followed by filtration and washing of the resin with DMF (6x), DCM (6x), DMF (6x). Successful deprotection and cyclization was confirmed via LC-MS analysis of the peptide fraction (**4**) obtained after a small scale cleavage.

Total deprotection and purification (4)

Total deprotection and cleavage from the acid-labile resin was performed using a solution of 0.1M HCl in HFIP (equivalent to 1 mL of ca. 37% HCl per 99 mL HFIP) to which 1% of scavenger, TIS, was added. After addition of 4 mL of this cleavage solution to the peptidyl resin (0.0716 mmol), the reaction vessel was shaken for 1 hour. The mixture was filtered and the resin was washed with DCM. This cleavage from the resin was repeated 2 more times. The combined filtrates were collected in a falcon tube and dried using an argon stream to obtain the crude peptide. To avoid incomplete deprotection, the dried fraction in the falcon tube was dissolved in the cleavage solution and reacted for another 4 hours. The volatile solution was evaporated using argon and the solid remainder with the crude peptide was precipitated in cold MTBE. After, the MTBE solution was centrifuged for 8 minutes at 5°C, the solution containing the protecting groups was decanted and the precipitation step was repeated 2 more times. The crude peptide **4** was obtained as a whitish-yellow precipitate.

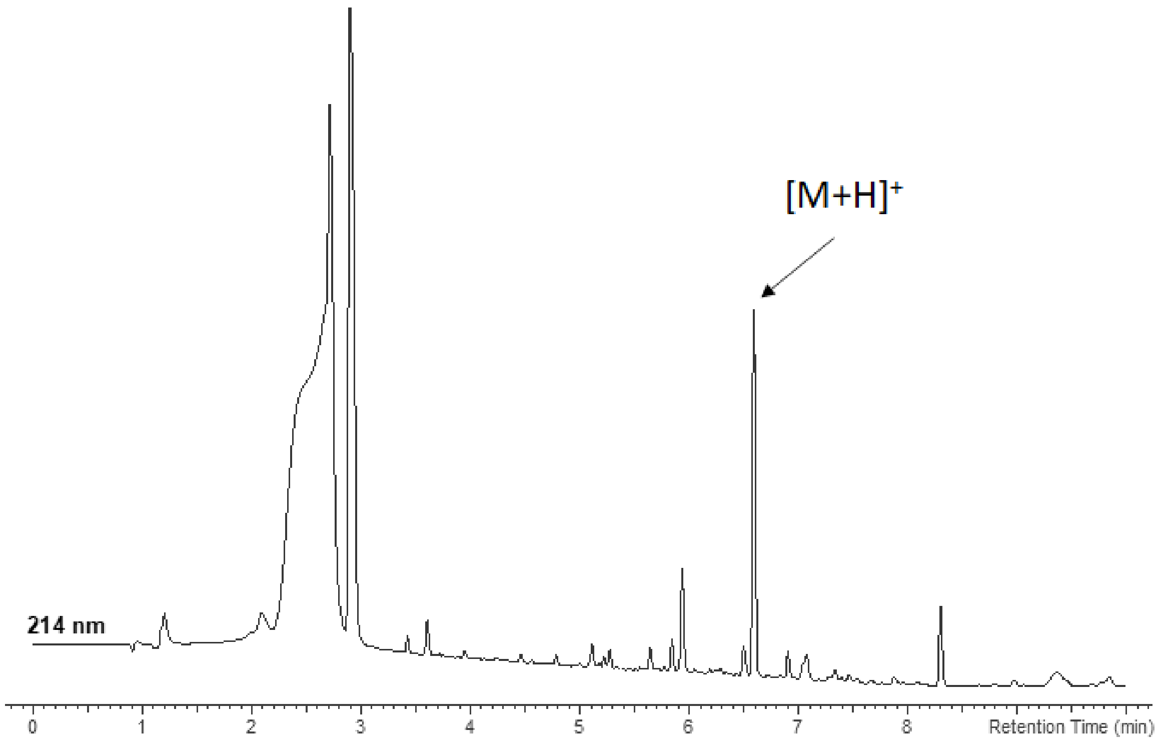

Figure S27: RP-HPLC chromatogram of crude (**4**). Kinetex C18 column with elution by a linear gradient over 6 min of 5 mM NH_4OAc in H_2O and CH_3CN from 100:0 to 0:100. Detection at a wavelength of λ=214 nm. $[M+H]^+$ was determined to be 1282.7 which is in agreement with the proposed chemical formula $C_{63}H_{112}N_{10}O_{17}$.

The crude peptide was purified by preparative reversed phase HPLC using a two solvent system consisting of H_2O (A) and CH_3CN (B), both contain 0.1% TFA. Separation of the main compound from the minor impurities present in the crude mixture, was achieved using a stepwise gradient going from 100:0 (A:B) to 50:50 in 10 minutes, from 50:50 to 40:60 in 5 minutes and from 40:60 to 0:100 in 40 minutes. The synthesis yielded 7.16 mg of pure (10:8) 1L. The overall yield starting from the initial resin loading is 5.11 %

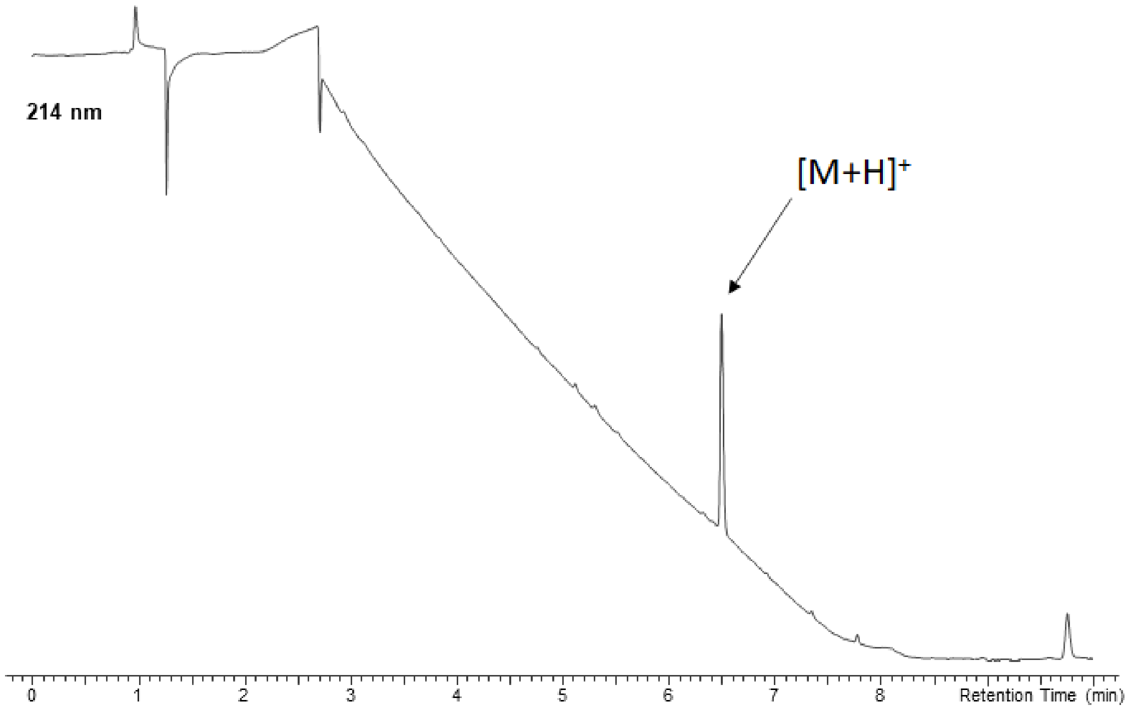

Figure S28: RP-HPLC chromatogram of purified **4**. Kinetex C18 column with elution by a linear gradient over 6 min of 5 mM NH_4OAc in H_2O and CH_3CN from 100:0 to 0:100. Detection at a wavelength of λ=214 nm.

Synthesis of (10:8) 5L (5)

The synthesis of the (10:8) analogue with a L-leucine at position 5 and an D-leucine at position 1, proceeded according to the same synthesis strategy described above for (10:8) 1D. The synthesis was started from the preloaded resin **7** (0.358 mmol/g; 200 mg; 0.0716 mmol) and all building blocks were available. The synthesis proceeded under identical conditions as mentioned above. Although the crude purity seems quite low, LC-MS analysis confirmed the successful synthesis of (10:8) 5L (**5**) after total deprotection and cleavage from the resin using the standard procedure with 0.1M HCl in HFIP and MTBE work-up.

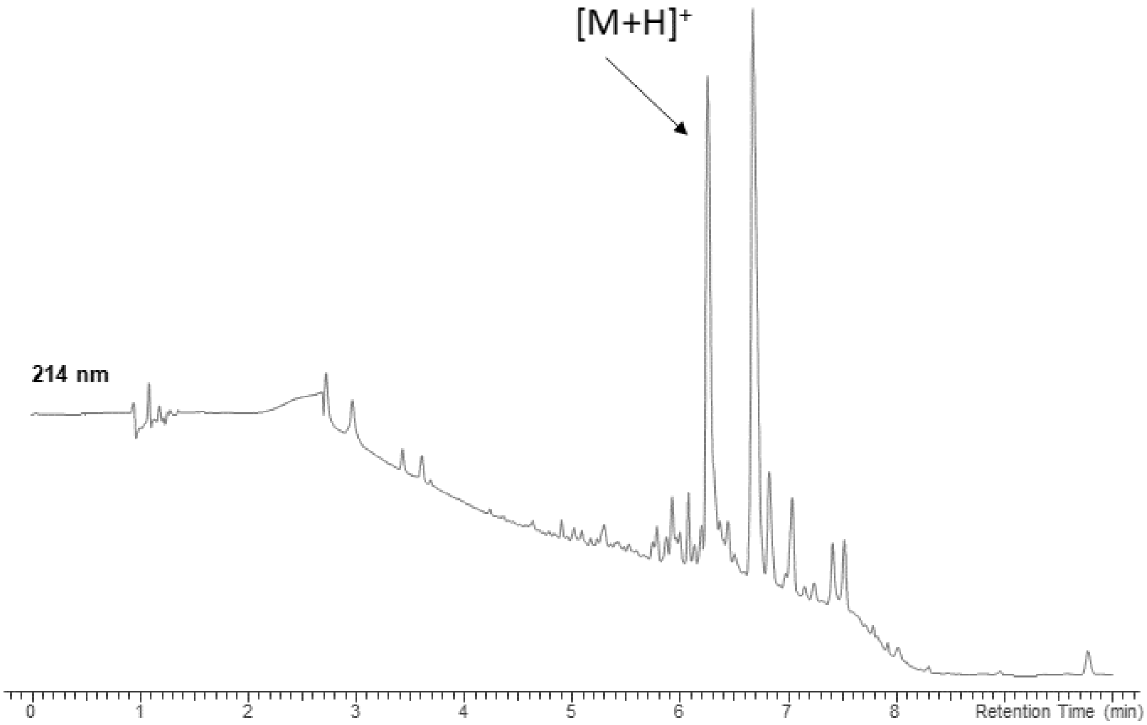

Figure S29: LC-MS chromatogram of crude (**5**). Kinetex C18 column with elution by a linear gradient over 6 min of 5 mM NH_4OAc in H_2O and CH_3CN from 100:0 to 0:100. Detection at a wavelength of λ=214 nm. $[M+H]^+$ was determined to be 1282.7 which is in agreement with $C_{63}H_{112}N_{10}O_{17}$.

The crude of peptide **5** was purified by preparative RP- HPLC consisting of the following solvent system: H_2O + 5 mM NH_4OAc (A) and CH_3CN (B). The elution proceeded via a stepwise gradient going from 100:0 to 60:40 in 8 minutes, to 50:50 in 5 minutes, to 40:60 in 10 minutes, to 10:90 in 90 minutes and finally to 0:100 in 7 minutes. The pure fractions were combined and lyophilized resulting in 7.16 mg of pure (10:8) 5L. The overall yield starting from the initial resin loading is 8.88%.

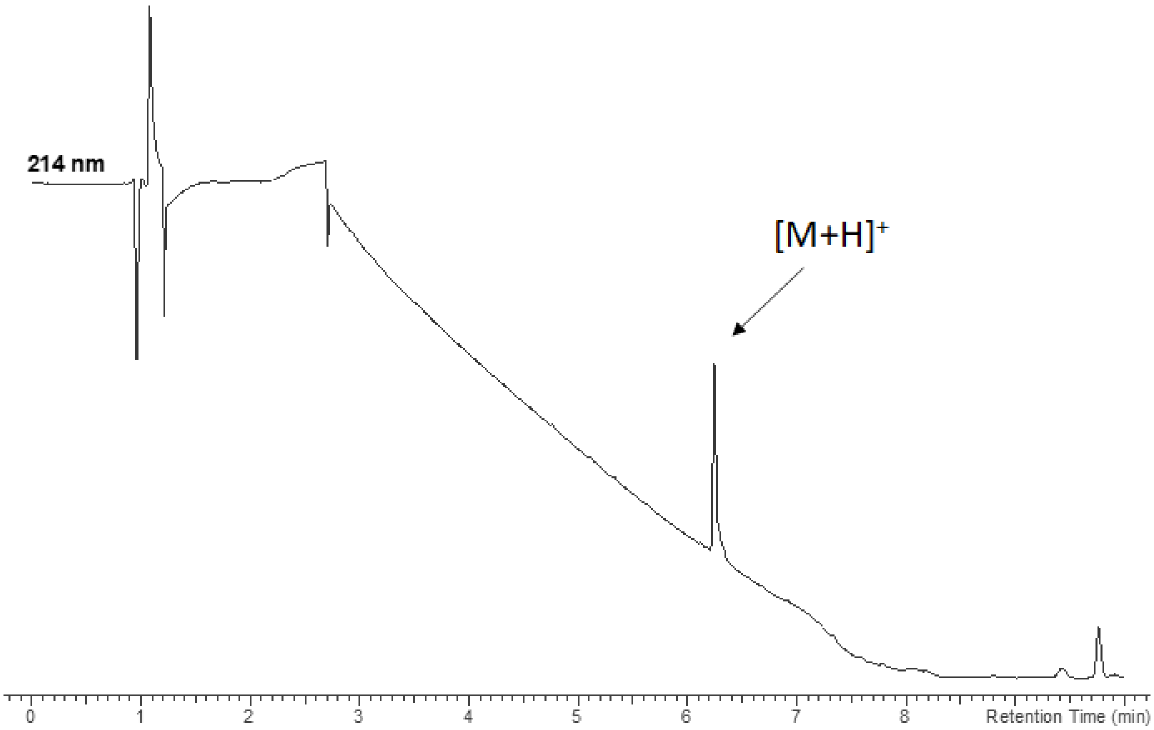

Figure S30: Chromatogram of purified (**5**). Kinetex C18 column with elution by a linear gradient over 6 min of 5 mM NH_4OAc in H_2O and CH_3CN from 100:0 to 0:100. Detection at a wavelength of λ=214 nm. $[M+H]^+$ was determined to be 1282.7 which is in agreement with the proposed chemical formula $C_{63}H_{112}N_{10}O_{17}$.

Synthesis of (10:8) L1L5 (6)

For the synthesis of the all L analogue containing L-leu at both position 1 and 5, identical procedures were used as for previous analogues. Having all building blocks in hand, the peptide synthesis started from the preloaded resin **7** (0.37 mmol/g; 200 mg; 0.074 mmol). LC-MS analysis of peptide **6** obtained after final cleavage, confirmed successful synthesis of the (10:8) 1L5L analogue.

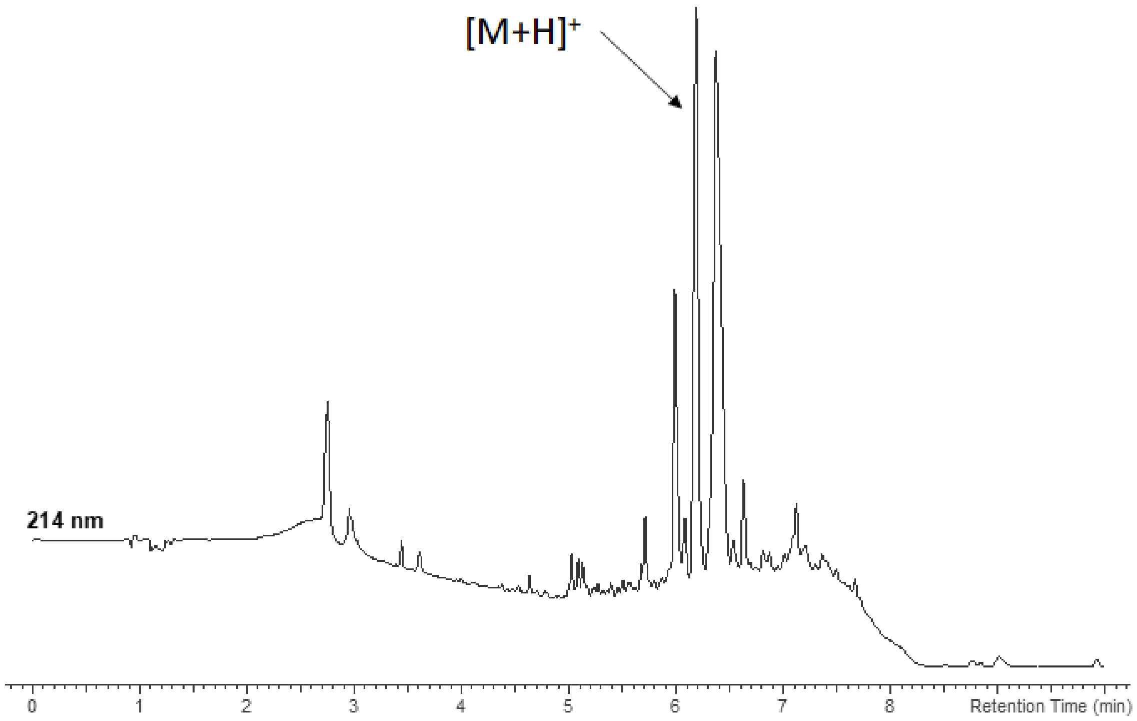

Figure S31: LC-MS chromatogram of crude (**6**). Kinetex C18 column with elution by a linear gradient over 6 min of 5 mM NH_4OAc in H_2O and CH_3CN from 100:0 to 0:100. Detection at a wavelength of λ=214 nm. $[M+H]^+$ was determined to be 1282.7 which is in agreement with $C_{63}H_{112}N_{10}O_{17}$.

The standard procedure for final cleavage and total deprotection using 0.1M HCl in HFIP was applied, followed by work-up with MTBE. The crude peptide **6** was obtained as a whitish powder and purified using the same gradient and solvent system as for (10:8) 5D. The overall yield starting from the initial resin loading is 5.11 %.

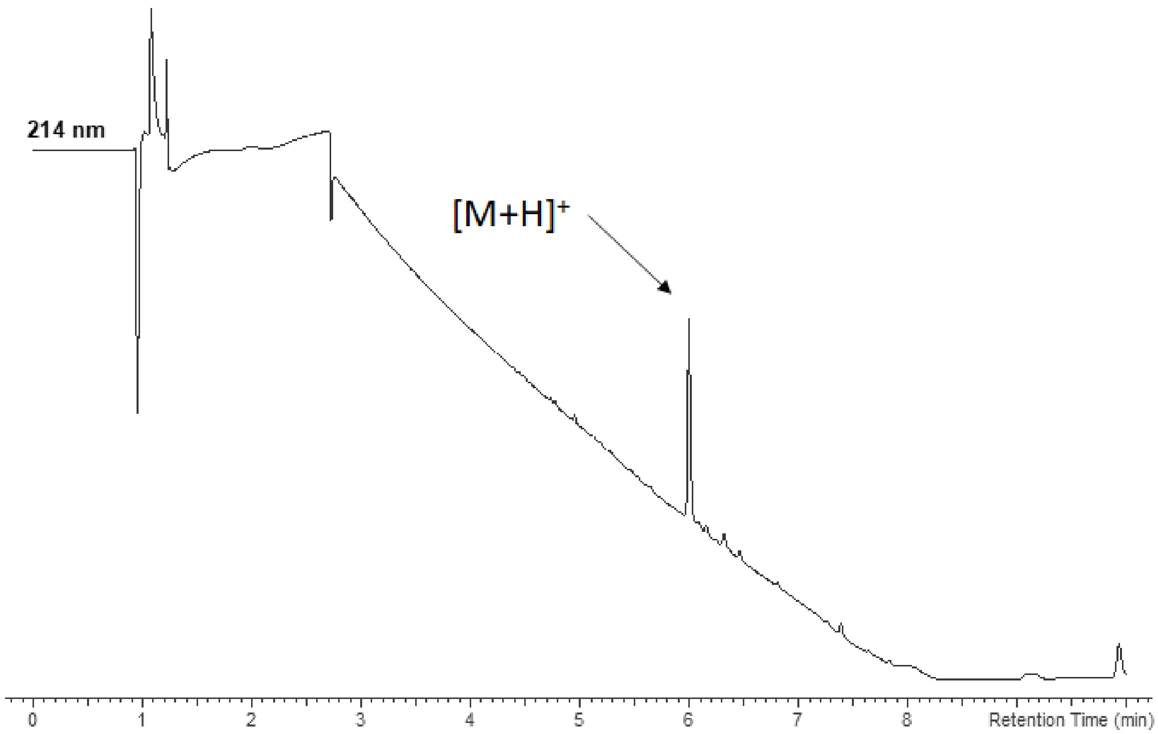

Figure S32: Chromatogram of purified (**6**). Kinetex C18 column with elution by a linear gradient over 6 min of 5 mM NH_4OAc in H_2O and CH_3CN from 100:0 to 0:100. Detection at a wavelength of λ=214 nm. $[M+H]^+$ was determined to be 1282.7 which is in agreement with the proposed chemical formula $C_{63}H_{112}N_{10}O_{17}$.

NMR characterization of cyclic lipodepsipeptides 4-6

Analysis of peptide (10:8)-1L (4)

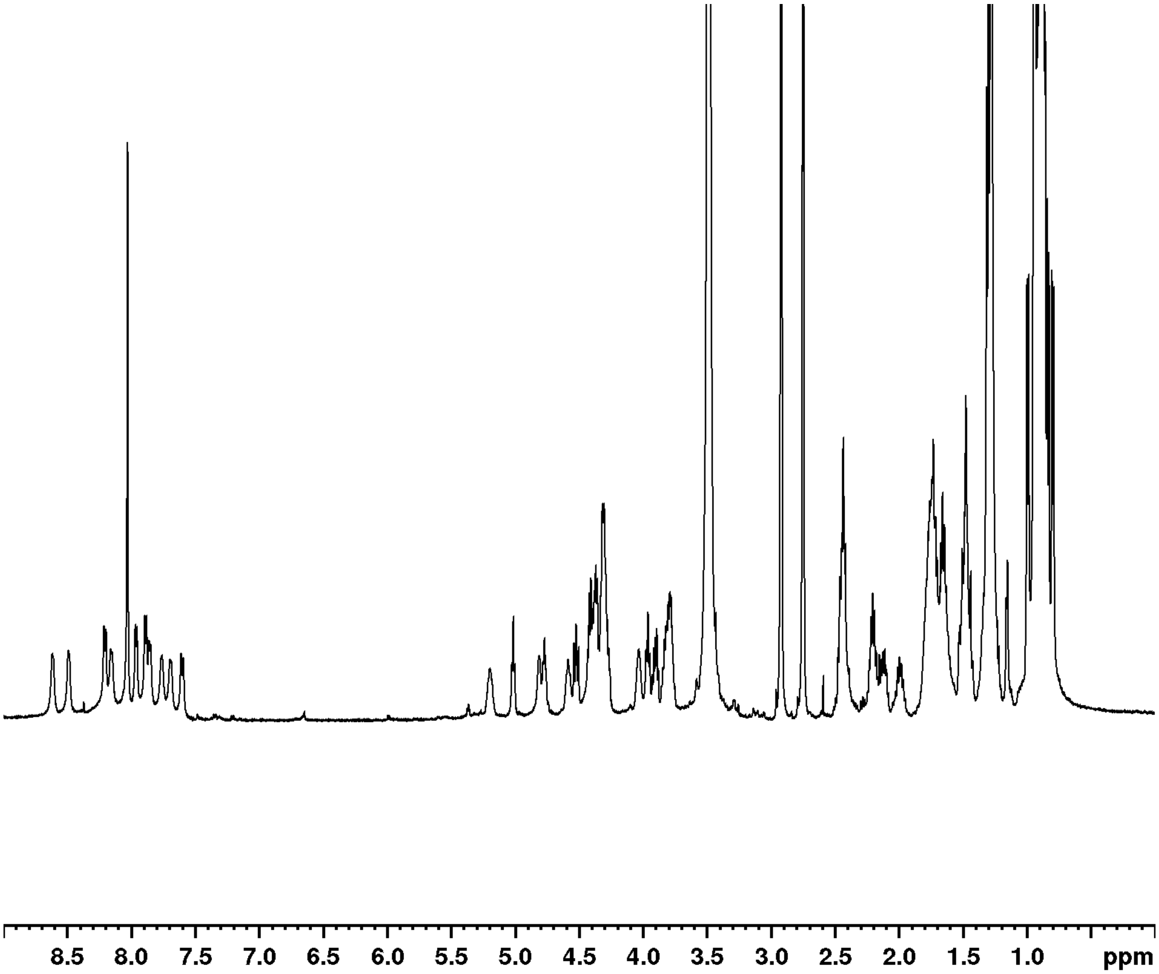

Figure S33: 1D ^{1}H spectrum of the synthetic orfamide-analogue (10:8)-1L **(4)** (DMF-d7, 298K, 500 MHz)

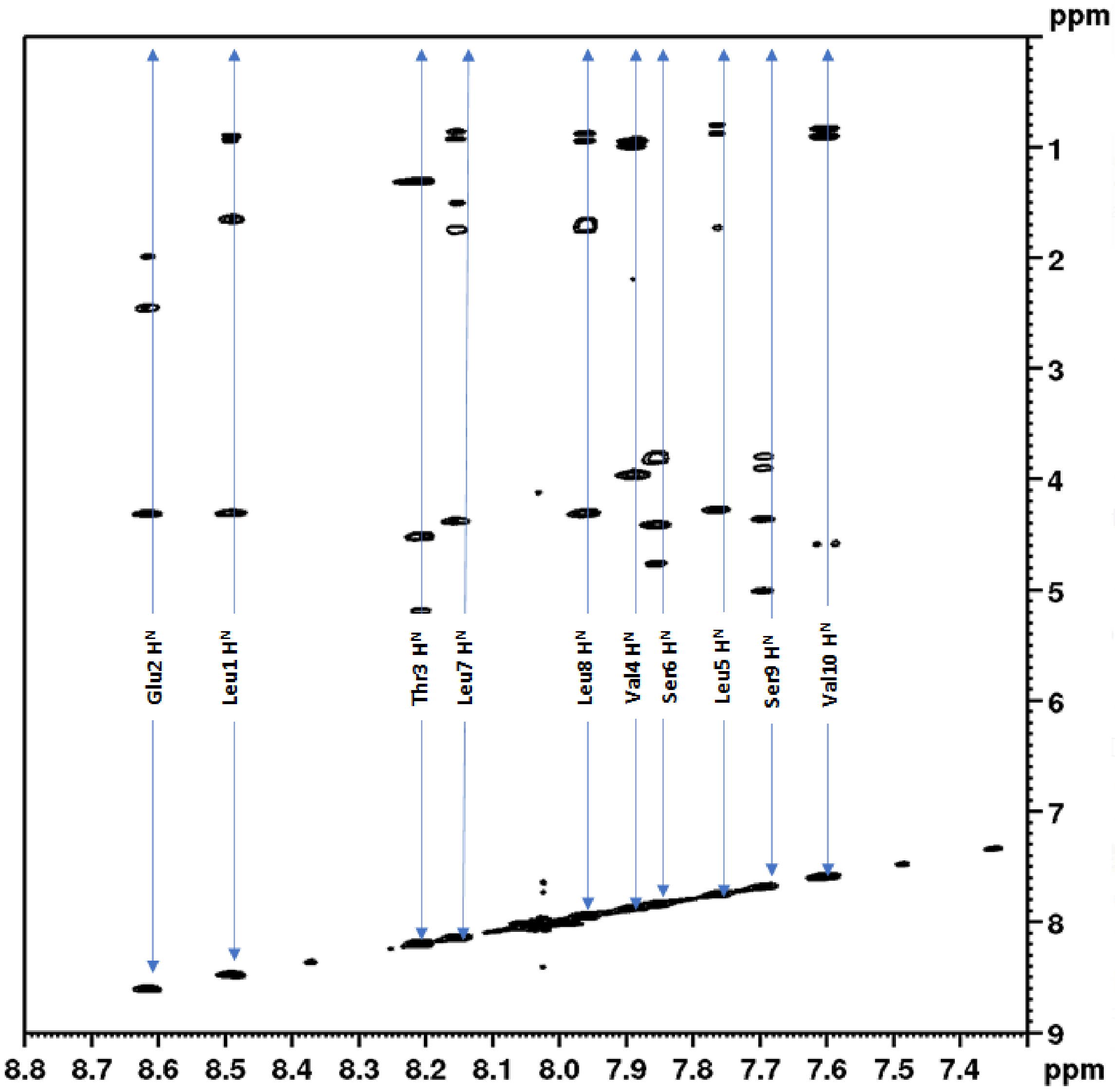

Figure S34: ^1H-^1H TOCSY traces of the individual amino acids in the synthetic orfamide-analogue (10:8)-1L **(4)**.(500MHz, DMF-*d7*, 298K)

Table S11: NMR assignment (^{1}H and ^{13}C) of the synthetic orfamide-analogue (10:8)-1L **(4)**. (500MHz, DMF-d7, 298K)

		**^{1}H δ [ppm]**	**^{13}C δ [ppm]**			**^{1}H δ [ppm]**	**^{13}C δ [ppm]**
**FA**				**D-Leu5**			
	CO	-	172.96		NH	7.77	-
	CH_2α	2.43	43.73		CHα	4.27	53.19
	CHβ	4.03	68.41		CO	-	n.d.
	CH_2γ	1.47	37.72		CH_2β1	1.73	40.00
	CH_2δ1	1.45	25.63		CH_2β2	1.51	40.00
	CH_2δ2	1.34	25.67		CHγ	1.78	24.49
	CH_2ε	1.28	29.47		CH_3δ1	0.88	23.01
	CH_2ζ	1.28	29.55		CH_3δ2	0.80	20.67
	CH_2η	1.28	29.55	**D-Ser6**			
	CH_2θ	1.28	29.55		NH	7.86	-
	CH_2ι	1.28	29.55		CHα	4.41	57.08
	CH_2κ	1.28	29.55		CO	-	172.03
	CH_2λ	1.27	31.85		CH_2β1	3.83	62.15
	CH2μ	1.29	22.53		CH_2β2	3.78	62.15
	CH_3ν	0.88	13.75		OHγ	4.76	-
	OH	n.d.	-	**L-Leu7**			
**L-Leu1**					NH	8.15	-
	NH	8.50	-		CHα	4.38	52.74
	CHα	4.30	52.41		CO	-	174.11
	CO	-	n.d.		CH_2β1	1.73	40.77
	CH_2β1	1.65	40.21		CH_2β2	1.51	40.77
	CH_2β2	1.65	40.21		CHγ	1.78	24.49
	CHγ	1.73	24.66		CH_3δ1	0.93	22.89
	CH_3δ1	0.90	21.51		CH_3δ2	0.87	21.05
	CH_3δ2	0.94	22.71	**L-Leu8**			
**D-Glu2**					NH	7.95	-
	NH	8.62	-		CHα	4.31	52.85
	CHα	4.31	54.22		CO	-	n.d.
	CO	-	173.40		CH_2β1	1.73	40.74
	CH_2β1	2.12	26.68		CH_2β2	1.51	40.74
	CH_2β2	1.98	26.68		CHγ	1.78	24.59
	CH_2γ	2.45	30.22		CH_3δ1	0.94	22.68
	COδ	-	173.97		CH_3δ2	0.88	21.14
	OH	n.d.	-	**D-Ser9**			
**D-aThr3**					NH	7.70	-
	NH	8.21	-		CHα	4.35	56.41
	CHα	4.52	58.44		CO	-	171.51
	CO	-	n.d.		CH_2β1	3.90	62.23
	CHβ	5.19	70.20		CH_2β2	3.79	62.23
	CH_3γ	1.31	17.58		OHγ	4.37	-
**D-Val4**				**L-Val10**			
	NH	7.89	-		NH	7.60	-
	CHα	3.95	60.92		CHα	4.58	57.80
	CO	-	n.d.		CO	-	n.d.
	CHβ	2.19	29.73		CHβ	2.21	30.51
	CH_3γ1	0.94	19.07		CH_2γ1	0.83	18.92
	CH_3γ2	0.99	19.07		CH_2γ2	0.91	18.96

Analysis of peptide (10:8)-5L (5)

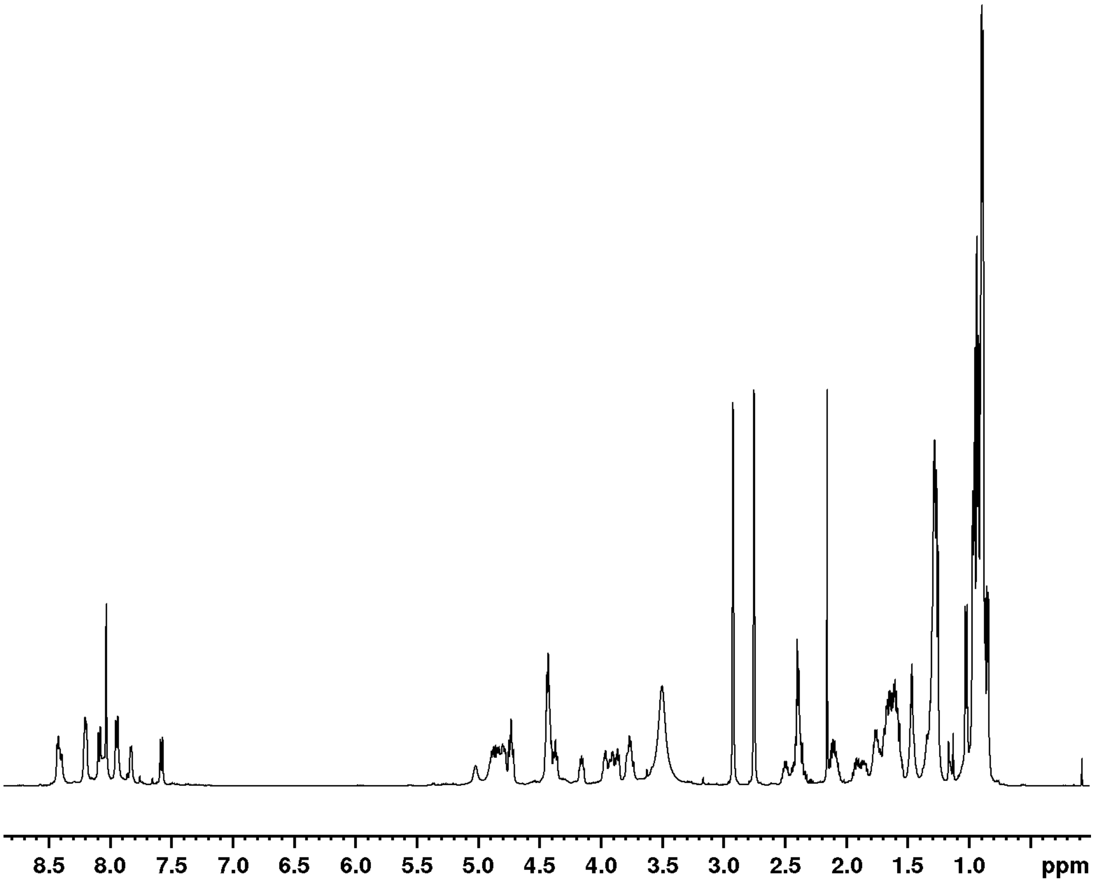

Figure S35: 1D ^{1}H spectrum of the synthetic orfamide-analogue (10:8)-5L **(5)** (DMF-d7, 298K, 500 MHz)

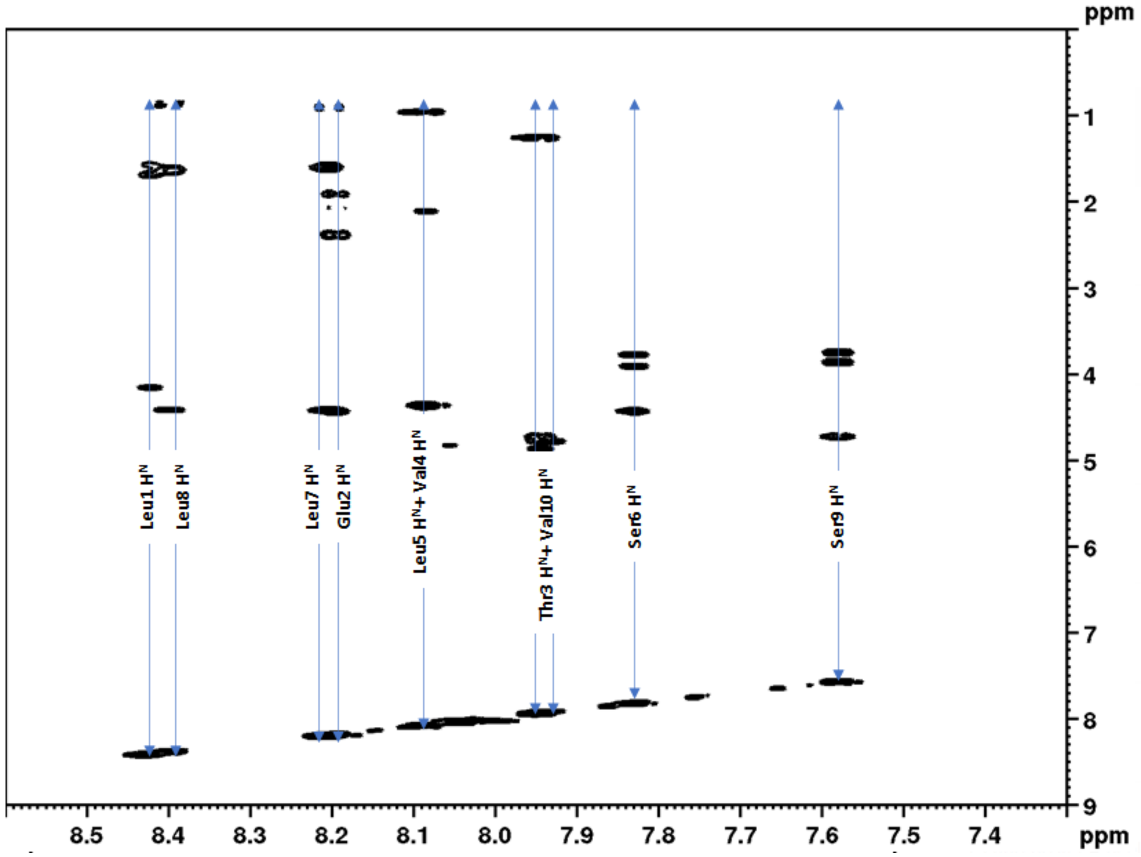

Figure S36: ^1H-^1H TOCSY traces of the individual amino acids in the synthetic orfamide-analogue (10:8)-5L **(5)**.(500MHz, DMF-*d7*, 298K)

Table S12: NMR assignment (^{1}H and ^{13}C) of the synthetic orfamide-analogue (10:8)-5L **(5)**. (500MHz, DMF-d7, 298K)

		**^{1}H δ [ppm]**	**^{13}C δ [ppm]**			**^{1}H δ [ppm]**	**^{13}C δ [ppm]**
**FA**				**L-Leu5**			
	CO	-	172.96		NH	8.06	-
	CH_2α	2.39	43.87		CHα	4.84	52.11
	CHβ	3.96	68.85		CO	-	172.70
	CH_2γ	1.47	37.52		CH_2β1	1.65	42.63
	CH_2δ1	1.47	37.50		CH_2β2	1.60	42.63
	CH_2δ2	1.34	25.67		CHγ	1.75	24.73
	CH_2ε	1.28	29.47		CH_3δ1	0.94	22.30
	CH_2ζ	1.28	29.63		CH_3δ2	0.90	22.47
	CH_2η	1.28	29.29	**D-Ser6**			
	CH_2θ	1.28	31.85		NH	7.83	-
	CH_2ι	1.28	29.55		CHα	4.43	56.36
	CH_2κ	1.28	29.55		CO	-	172.92
	CH_2λ	1.27	31.85		CH_2β1	3.92	62.23
	CH2μ	1.29	22.65		CH_2β2	3.78	62.23
	CH_3ν	0.88	13.75		OHγ	n.d.	-
	OH	n.d.	-	**L-Leu7**			
**D-Leu1**					NH	8.20	-
	NH	8.42	-		CHα	4.43	51.99
	CHα	4.16	54.21		CO	-	174.24
	CO	-	n.d.		CH_2β1	1.60	40.80
	CH_2β1	1.56	40.89		CH_2β2	1.60	40.80
	CH_2β2	1.69	40.89		CHγ	1.75	24.73
	CHγ	1.85	24.65		CH_3δ1	0.93	22.83
	CH_3δ1	0.93	22.81		CH_3δ2	0.87	21.12
	CH_3δ2	0.94	21.21	**L-Leu8**			
**D-Glu2**					NH	8.39	-
	NH	8.20	-		CHα	4.43	51.99
	CHα	4.43	52.94		CO	-	173.19
	CO	-	174.24		CH_2β1	1.64	40.02
	CH_2β1	2.09	27.12		CH_2β2	1.64	40.02
	CH_2β2	1.91	27.12		CHγ	1.67	24.77
	CH_2γ	2.39	30.31		CH_3δ1	0.94	22.83
	COδ	-	n.d.		CH_3δ2	0.88	21.12
	OH	n.d.	-	**D-Ser9**			
**D-aThr3**					NH	7.57	-
	NH	7.94	-		CHα	4.72	51.99
	CHα	4.73	56.80		CO	-	170.92
	CO	-	171.93		CH_2β1	3.87	62.76
	CHβ	4.88	71.34		CH_2β2	3.87	62.76
	CH_3γ	1.26	17.12		OHγ	n.d.	-
**D-Val4**				**L-Val10**			
	NH	8.08	-		NH	7.93	-
	CHα	4.36	58.64		CHα	4.78	56.55
	CO	-	172.92		CO	-	n.d.
	CHβ	2.11	30.97		CHβ	2.49	30.18
	CH_3γ1	0.96	18.05		CH_2γ1	1.02	18.81
	CH_3γ2	0.96	19.06		CH_2γ2	0.91	16.73

--

Analysis of peptide (10:8)-1L5L (6)

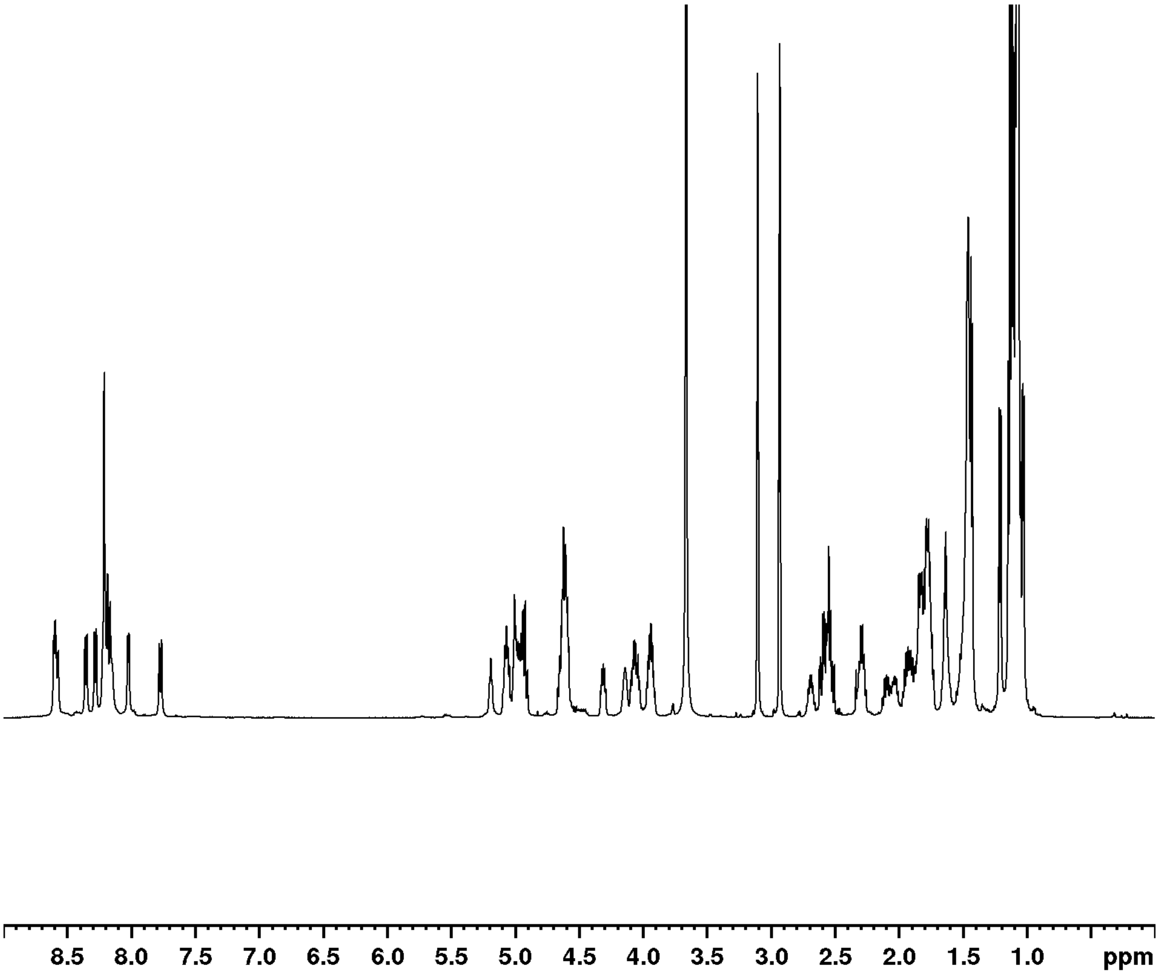

Figure S37: 1D ^{1}H spectrum of the synthetic orfamide-analogue (10:8)-1L5L **(6)** (DMF-d7, 298K, 500 MHz)

Table S13: NMR assignment (^{1}H and ^{13}C) of the synthetic orfamide-analogue (10:8)-L1L5 **(6)**. (500MHz, 90%acetonitrile-d3/10%H_2O, 298K)

		**^{1}H δ [ppm]**	**^{13}C δ [ppm]**			**^{1}H δ [ppm]**	**^{13}C δ [ppm]**
**<u>FA</u>**				**L-Leu5**			
	CO	-	173.37		NH	7.71	-
	CH$_2$α	2.39	43.06		CHα	4.42	52.25
	CHβ	3.93	68.58		CO	-	172.75
	CH$_2$γ	1.43	36.84		CH$_2$β1	1.65	42.63
	CH$_2$δ1	1.28	25.41		CH$_2$β2	1.60	42.63
	CH$_2$δ2	1.39	25.41		CHγ	1.75	24.73
	CH$_2$ε	1.28	29.20		CH$_3$δ1	0.88	22.30
	CH$_2$ζ	1.28	29.20		CH$_3$δ2	0.90	22.27
	CH$_2$η	1.28	31.61	**D-Ser6**			
	CH$_2$θ	1.28	22.36		NH	7.71	-
	CH$_2$ι	n.d.	n.d.		CHα	4.40	55.65
	CH$_2$κ	n.d.	n.d.		CO	-	171.76
	CH$_2$λ	n.d.	n.d.		CH$_2$β1	3.72	61.72
	CH2μ	n.d.	n.d.		CH$_2$β2	3.81	61.72
	CH$_3$ν	n.d.	n.d.		OHγ	n.d.	-
	OH	n.d.	-	**L-Leu7**			
**L-Leu1**					NH	7.87	-
	NH	7.12	-		CHα	4.24	53.36
	CHα	4.21	52.37		CO	-	173.77
	CO	-	173.88		CH$_2$β1	1.59	40.40
	CH$_2$β1	1.58	39.77		CH$_2$β2	1.52	40.40
	CH$_2$β2	1.58	39.77		CHγ	1.64	24.51
	CHγ	1.64	24.50		CH$_3$δ1	0.93	22.83
	CH$_3$δ1	0.88	21.14		CH$_3$δ2	0.87	21.12
	CH$_3$δ2	0.94	22.24	**L-Leu8**			
**D-Glu2**					NH	7.62	-
	NH	7.97	-		CHα	4.32	52.37
	CHα	4.29	53.36		CO	-	173.19
	CO	-	172.92		CH$_2$β1	1.64	39.62
	CH$_2$β1	2.05	26.26		CH$_2$β2	1.64	40.02
	CH$_2$β2	1.91	26.26		CHγ	1.67	24.77
	CH$_2$γ	2.39	30.31		CH$_3$δ1	0.94	22.83
	COδ	-	n.d.		CH$_3$δ2	0.88	21.12
	OH	n.d.	-	**D-Ser9**			
**D-aThr3**					NH	7.57	-
	NH	7.95	-		CHα	4.44	55.79
	CHα	4.51	57.13		CO	-	170.92
	CO	-	170.59		CH$_2$β1	3.78	62.76
	CHβ	5.08	70.56		CH$_2$β2	3.83	62.76
	CH$_3$γ	1.23	16.36		OHγ	n.d.	-
**D-Val4**				**L-Val10**			
	NH	7.14	-		NH	7.45	-
	CHα	3.99	60.02		CHα	4.40	56.55
	CO	-	172.02		CO	-	172.89
	CHβ	2.05	30.27		CHβ	2.23	30.18
	CH$_3$γ1	0.96	18.11		CH$_2$γ1	0.84	18.81
	CH$_3$γ2	0.96	18.51		CH$_2$γ2	0.91	16.73

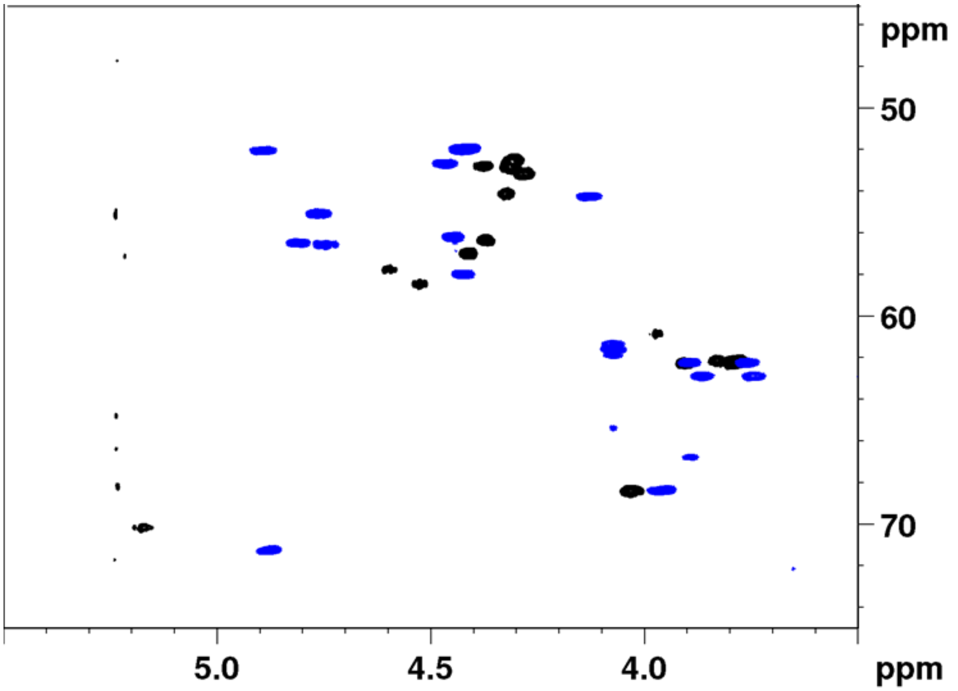

Figure S38: Comparison of the ^{1}H-{^{13}C} HSQC (CH)α fingerprint of synthetic (8:6)-L1L5 (blue) with that of the natural compound extracted from *P. aestus* CMR5c, recorded in DMF-d7 at 500 MHz and 298K

Analysis of the natural orfamide A from *Pseudomonas protegens* Pf-5

Extraction and purification of natural CLiP

After growth of *P. protegens* Pf-5, the cyclic lipopeptides produced by this bacterial strain was extracted from the supernatant and cell content. In total 8 fractions were collected and examined by mass analysis. The main compound eluted at a retention time of 20.6 min in Fig S1. This peptide fraction was purified via semi-preparative RP-HPLC analysis (Figure S39), using a two solvent system: H_2O + 01% TFA (A) and CH_3CN (B), via application of a linear gradient over 15 minutes going from 25:75 to 0:100 (A:B).

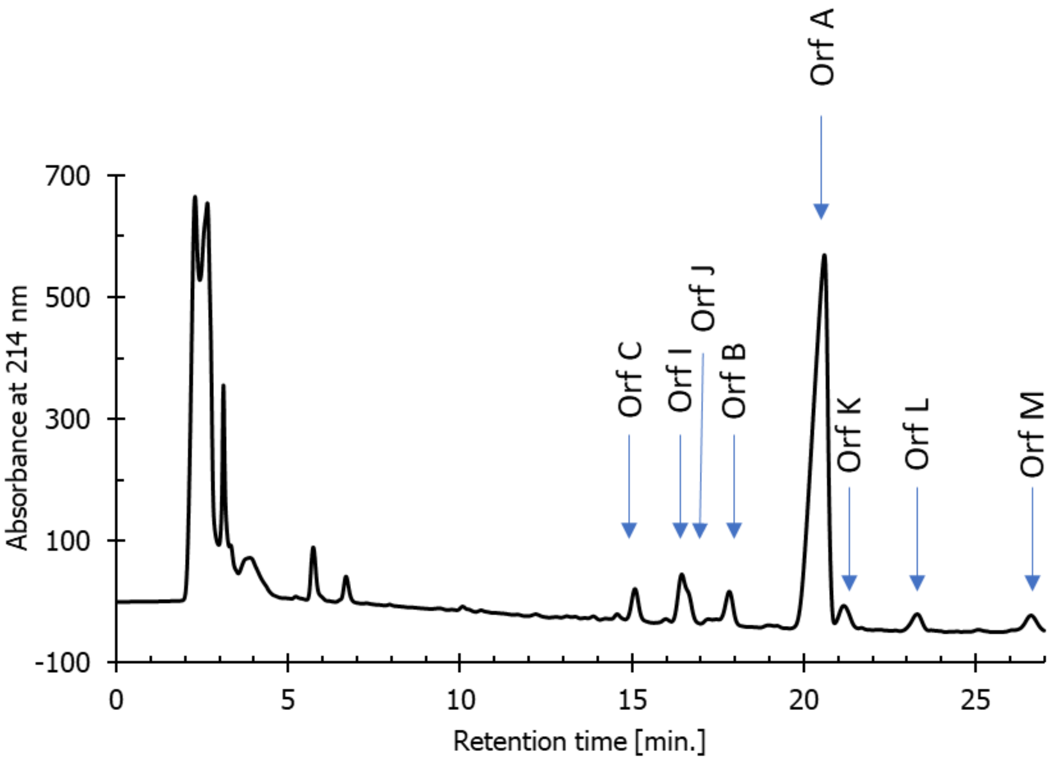

Figure S39: Semi-preparative reversed-phase HPLC chromatogram of *P. protegens* Pf-5, showing the clear presence of orfamide A (at 20.6 minutes) and its minor compounds. .

	Fatty acid	1	2	3	4	5	6	7	8	9	10
**Orfamide A**	3-OH C14:0	Leu	Glu	aThr	alle	Leu	Ser	Leu	Leu	Ser	Val
**Orfamide B**	3-OH C14:0	Leu	Glu	aThr	Val	Leu	Ser	Leu	Leu	Ser	Val
**Orfamide C**	3-OH C12:0	Leu	Glu	aThr	alle	Leu	Ser	Leu	Leu	Ser	Val
**Orfamide I**	3-OH C14:0	Leu	Glu^{Me}	aThr	alle	Leu	Ser	Leu	Leu	Ser	Val
**Orfamide J**	3-OH C14:0	Leu	Glu	aThr	Leu	Leu	Ser	Leu	Leu	Ser	Val
**Orfamide K**	3-OH C14:1	Leu	Glu	aThr	alle	Leu	Ser	Leu	Leu	Ser	Val
**Orfamide L**	3-OH C14:0	Leu	Glu	aThr	alle	Leu	Ser	Leu	Leu	Ser	Ile
**Orfamide M**	3-OH C16:0	Leu	Glu	aThr	alle	Leu	Ser	Leu	Leu	Ser	Val

Figure S40: Schematic overview of the orfamide homologues that were characterized from *Pseudomonas protegens* Pf-5.

NMR characterization of orfamides extracted from *P. protegens* Pf-5

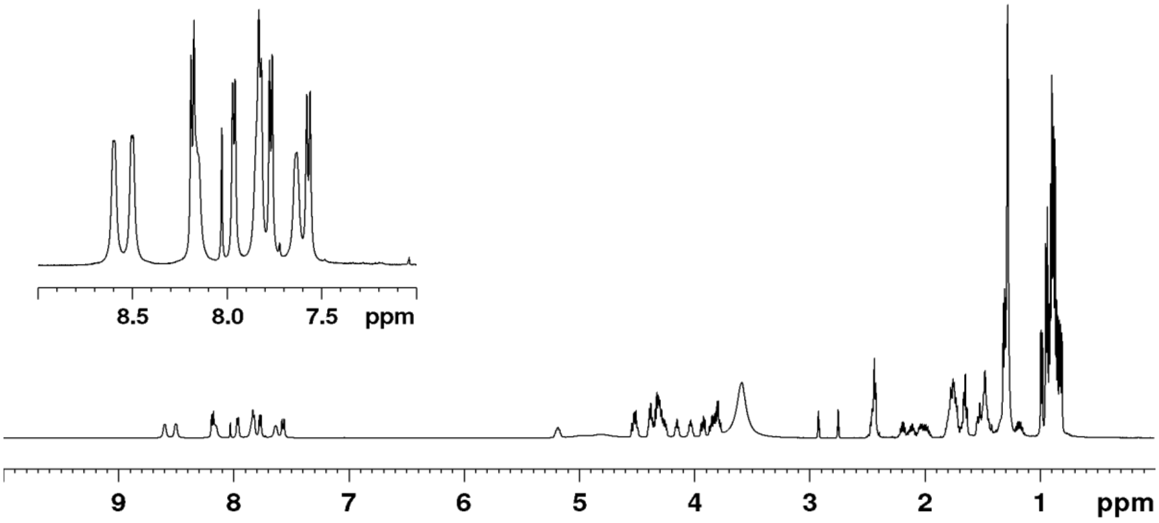

Figure S41: 1D ^{1}H spectrum of the orfamide A as extracted from *P. protegens* Pf-5. (DMF-d7, 298K, 500 MHz)

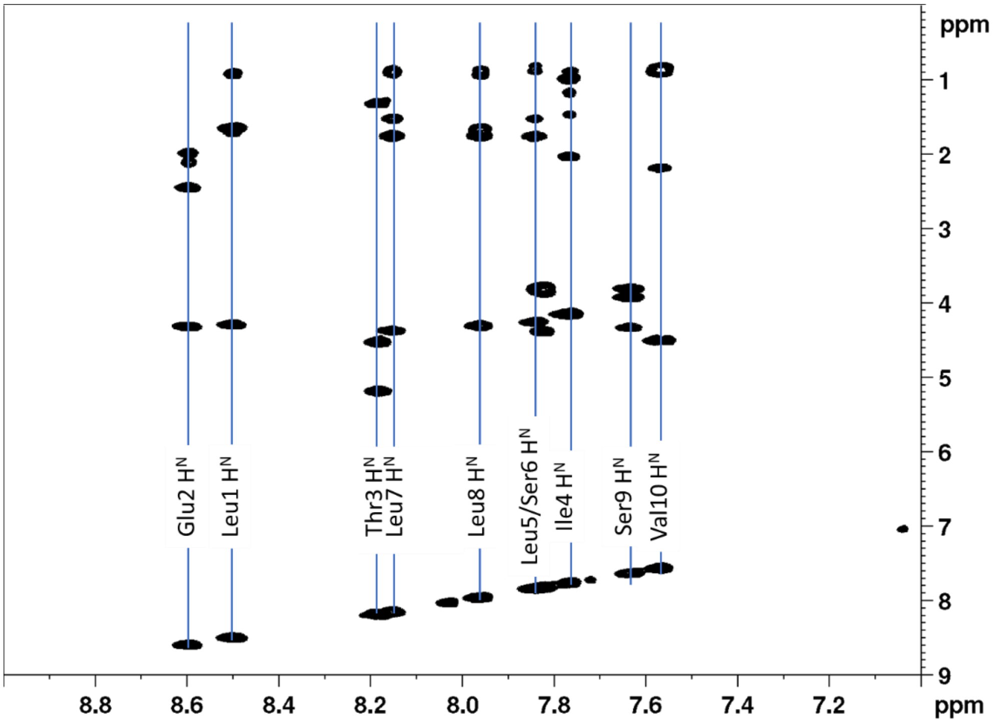

Figure S42: ^{1}H-^{1}H TOCSY traces of the individual amino acids in orfamide A as isolated from *P. protegens* Pf-5.(500MHz, DMF-*d7*, 298K)

Table S14: NMR assignment (^{1}H and ^{13}C) of orfamide A, extracted as the main compound produced by *P. protegens* Pf-5. (500MHz, DMF-d7, 298K)

	^{1}H δ [ppm]	^{13}C δ [ppm]		^{1}H δ [ppm]	^{13}C δ [ppm]
**FA**			D-**Leu5**		
CO	-	173.13	NH	7.84	-
$CH_2α$	2.43	43.69	CHα	4.26	53.33
CHβ	4.03	68.35	CO	-	173.47
$CH_2δ1$	1.48	25.60	$CH_2β1$	1.75	39.91
$CH_2δ2$	1.35	25.62	$CH_2β2$	1.52	39.94
CH_2	1.27	31.82	CHγ	1.76	24.60
CH_2	1.28	22.50	$CH_3δ1$	0.81	20.67
CH_2	1.28	29.61	$CH_3δ2$	0.89	22.93
CH_2	1.48	37.59	D-**Ser6**		
CH_3	0.88	13.72	NH	7.83	-
OH	n.d.	-	CHα	4.38	57.12
L-**Leu1**			CO	-	171.47
NH	8.50	-	$CH_2β1$	3.85	62.01
CHα	4.29	52.44	$CH_2β2$	3.79	62.01
CO	-	173.03	OH	n.d.	-
$CH_2β$	1.65	40.06	L-**Leu7**		
CHγ	1.76	24.60	NH	8.15	-
$CH_3δ1$	0.94	22.72	CHα	4.37	52.66
$CH_3δ2$	0.90	21.46	CO	-	173.50
D-**Glu2**			$CH_2β1$	1.75	40.65
NH	8.60	-	$CH_2β2$	1.52	40.68
CHα	4.32	54.10	CHγ	1.76	24.60
CO	-	172.90	$CH_3δ1$	0.92	22.91
$CH_2β1$	2.11	26.68	$CH_3δ2$	0.87	21.05
$CH_2β2$	1.98	26.68	L-**Leu8**		
$CH_2γ$	2.45	30.14	NH	7.96	-
CO	-	174.16	CHα	4.31	52.86
OH	n.d.	-	CO	-	174.16
D-**aThr3**			$CH_2β1$	1.76	40.37
NH	8.18	-	$CH_2β2$	1.66	40.37
CHα	4.52	58.41	CHγ	1.76	24.60
CO	-	171.18	$CH_3δ1$	0.94	22.72
CHβ	5.19	70.02	$CH_3δ2$	0.87	21.05
$CH_3γ$	1.31	17.44	D-**Ser9**		
D-**aIle4**			NH	7.63	-
NH	7.76	-	CHα	4.33	56.61
CHα	4.15	59.16	CO	-	170.84
CO	-	172.89	$CH_2β1$	3.92	62.09
CHβ	2.04	36.14	$CH_2β2$	3.80	62.09
$CH_3γ$	0.98	15.11	OH	n.d.	-
$CH_2γ$	1.17	25.96	L-**Val10**		
$CH_3δ$	0.88	11.11	NH	7.57	-
			CHα	4.50	57.86
			CO	-	171.37
			CHβ	2.19	30.26
			$CH_3γ1$	0.84	17.74
			$CH_3γ2$	0.90	18.97

Table S15: NMR assignment (^{1}H and ^{13}C) of orfamide A, extracted as the main compound produced by *P. protegens* Pf-5. (500MHz, methanol-d3, 298K)

	^{1}H δ [ppm]	^{13}C δ [ppm]
**FA**		
CO	-	173.13
CH_2α	2.41	44.35
CHβ	4.08	69.74
CH_2γ	1.50	38.43
CH_2δ1	1.49	26.49
CH_2δ2	1.33	26.51
CH_2	1.29	32.97
CH_2	1.30	30.62
CH_2	1.31	23.58
CH_3	0.90	14.32
OH	n.d.	-
**L-Leu1**		
NH	8.50	-
CHα	4.13	54.20
CO	-	173.03
CH_2β	1.64	40.71
CHγ	1.74	25.76
CH_3δ1	0.95	22.41
CH_3δ2	1.00	23.00
**D-Glu2**		
NH	8.60	-
CHα	4.17	55.90
CO	-	172.90
CH_2β1	2.14	26.99
CH_2β2	2.01	26.99
CH_2γ	2.47	30.92
CO	-	174.16
OH	n.d.	-
**D-aThr3**		
NH	8.18	-
CHα	4.34	60.62
CO	-	171.18
CHβ	5.38	70.50
CH_3γ	1.38	18.48
**D-aIle4**		
NH	7.76	-
CHα	3.98	61.59
CO	-	172.89
CHβ	2.01	37.01
CH_3γ	0.98	16.13
CH_2γ1	1.47	26.90
CH_2γ2	1.20	26.93
CH_3δ	0.92	11.68
**D-Leu5**		
NH	7.84	-
CHα	4.22	54.79
CO	-	173.47
CH_2β1	1.80	40.77
CH_2β2	1.56	40.78
CHγ	1.74	25.76
CH_3δ1	0.87	21.00
CH_3δ2	0.93	23.55
**D-Ser6**		
NH	7.83	-
CHα	4.35	58.19
CO	-	171.47
CH_2β1	3.94	62.65
CH_2β2	3.86	62.65
OH	n.d.	-
**L-Leu7**		
NH	8.15	-
CHα	4.47	53.61
CO	-	173.50
CH_2β1	1.79	41.71
CH_2β2	1.49	41.76
CHγ	1.74	25.76
CH_3δ1	0.92	21.47
CH_3δ2	0.96	23.64
**L-Leu8**		
NH	7.96	-
CHα	4.33	54.20
CO	-	174.16
CH_2β	1.71	41.36
CHγ	1.74	25.76
CH_3δ1	0.92	21.88
CH_3δ2	0.98	23.23
**D-Ser9**		
NH	7.63	-
CHα	4.30	57.78
CO	-	170.84
CH_2β1	3.90	62.48
CH_2β2	3.82	62.47
OH	n.d.	-
**L-Val10**		
NH	7.57	-
CHα	4.36	59.51
CO	-	171.37
CHβ	2.15	31.33
CH_3γ1	0.83	18.80
CH_3γ2	0.88	19.71

Table S16: NMR assignment (^{1}H and ^{13}C) of orfamide C, extracted as the minor compound produced by *P. protegens* Pf-5. (500MHz, DMF-d7, 298K)

	^{1}H δ [ppm]	^{13}C δ[ppm]		^{1}H δ[ppm]	^{13}C δ[ppm]
**FA**			D-**Leu5**		
CO	-	n.d.	NH	7.87	-
CH_2α	2.44	43.80	CHα	4.26	53.41
CHβ	4.03	68.45	CO	-	n.d.
CH_2δ1	1.48	25.73	CH_2β1	1.76	39.95
CH_2δ2	1.34	25.72	CH_2β2	1.53	40.00
CH_2	1.48	37.65	CHγ	1.77	24.60
CH_2	1.28	22.58	CH_3δ1	0.89	22.98
CH_2	1.28	29.67	CH_3δ2	0.82	20.70
CH_2	1.28	31.90			
CH_3	0.88	13.78	D-**Ser6**		
OH	4.84	-	NH	7.83	-
			CHα	4.39	57.30
L-**Leu1**			CO	-	n.d.
NH	8.54	-	CH_2β1	3.85	62.08
CHα	4.31	52.44	CH_2β2	3.78	62.07
CO	-	n.d.	OH	4.84	-
CH_2β	1.66	40.10			
CHγ	1.77	24.60	L-**Leu7**		
CH_3δ1	0.90	21.48	NH	8.16	-
CH_3δ2	0.94	22.81	CHα	4.38	52.61
			CO	-	n.d.
D-**Glu2**			CH_2β1	1.75	40.72
NH	8.62	-	CH_2β2	1.52	40.76
CHα	4.31	54.29	CHγ	1.77	24.60
CO	-	n.d.	CH_3δ1	0.92	22.99
CH_2β1	2.11	26.75	CH_3δ2	0.87	21.11
CH_2β2	2.00	26.79			
CH_2γ	2.45	30.26	L-**Leu8**		
CO	-	n.d.	NH	7.97	-
OH	n.d.	-	CHα	4.30	52.93
			CO	-	n.d.
D-**aThr3**			CH_2β1	1.76	40.43
NH	8.24	-	CH_2β2	1.65	40.45
CHα	4.52	58.60	CHγ	1.77	24.60
CO	-	n.d.	CH_3δ1	0.94	22.81
CHβ	5.22	70.06	CH_3δ2	0.87	21.11
CH_3γ	1.31	17.53			
			D-**Ser9**		
D-**aIle4**			NH	7.65	-
NH	7.81	-	CHα	4.32	56.78
CHα	4.14	59.36	CO	-	n.d.
CO	-	n.d.	CH_2β1	3.93	62.16
CHβ	2.05	36.17	CH_2β2	3.80	62.16
CH_3γ	0.99	15.25	OH	4.99	-
CH_2γ	1.17	26.02			
CH_3δ	0.89	11.14	L-**Val10**		
			NH	7.55	-
			CHα	4.49	57.95
			CO	-	n.d.
			CHβ	2.19	30.33
			CH_3γ1	0.84	17.83
			CH_3γ2	0.90	19.03

Table S17: NMR assignment (^{1}H and ^{13}C) of orfamide J, extracted as the minor compound produced by *P. protegens* Pf-5. (500MHz, DMF-d7, 298K)

		^{1}H δ [ppm]	^{13}C δ [ppm]			^{1}H δ [ppm]	^{13}C δ [ppm]
**FA**				**D-Leu5**			
	CO	-	n.d.		NH	7.58	-
	CH$_2$α	2.42	43.67		CHα	4.29	52.91
	CHβ	4.05	68.42		CO	-	n.d.
	CH$_2$δ1	1.49	25.61		CH$_2$β1	1.78	40.14
	CH$_2$δ2	1.35	25.64		CH$_2$β2	1.54	40.11
	CH$_2$	1.49	37.75		CHγ	1.76	24.66
	CH$_2$	1.28	22.56		CH$_3$δ1	0.82	20.74
	CH$_2$	1.29	29.65		CH$_3$δ2	0.90	22.97
	CH$_2$	1.28	31.88				
	CH$_3$	0.88	13.76	**D-Ser6**			
	OH	4.83	-		NH	7.81	-
					CHα	4.38	57.32
**L-Leu1**					CO	-	n.d.
	NH	8.56	-		CH$_2$β1	3.88	62.27
	CHα	4.27	52.83		CH$_2$β2	3.77	62.27
	CO	-	n.d.		OH	4.78	-
	CH$_2$β	1.67	40.01				
	CHγ	1.76	24.66	**L-Leu7**			
	CH$_3$δ1	0.91	21.66		NH	8.00	-
	CH$_3$δ2	0.95	22.68		CHα	4.43	52.30
					CO	-	n.d.
**D-Glu2**					CH$_2$β1	1.78	40.67
	NH	8.77	-		CH$_2$β2	1.47	40.67
	CHα	4.26	54.64		CHγ	1.76	24.66
	CO	-	n.d.		CH$_3$δ1	0.86	21.03
	CH$_2$β1	2.12	26.58		CH$_3$δ2	0.93	23.07
	CH$_2$β2	2.02	26.55				
	CH$_2$γ	2.48	30.24	**L-Leu8**			
	CO	-	n.d.		NH	8.09	-
	OH	n.d.	-		CHα	4.29	52.91
					CO	-	n.d.
**D-aThr3**					CH$_2$β	1.70	40.25
	NH	8.30	-		CHγ	1.76	24.66
	CHα	4.49	58.90		CH$_3$δ1	0.87	21.45
	CO	-	n.d.		CH$_3$δ2	0.93	22.70
	CHβ	5.30	69.64				
	CH$_3$γ	1.32	17.64	**D-Ser9**			
					NH	7.89	-
**D-Leu4**					CHα	4.33	56.52
	NH	7.89	-		CO	-	n.d.
	CHα	4.16	53.76		CH$_2$β1	3.90	62.21
	CO	-	n.d.		CH$_2$β2	3.80	62.20
	CH$_2$β	1.70	40.25		OH	4.97	-
	CHγ	1.76	24.66				
	CH$_3$δ1	0.95	22.68	**L-Val10**			
	CH$_3$δ2	0.86	21.45		NH	7.58	-
					CHα	4.43	58.32
					CO	-	n.d.
					CHβ	2.16	30.27
					CH$_3$γ1	0.86	18.14
					CH$_3$γ2	0.89	19.01

Table S18: NMR assignment (^{1}H and ^{13}C) of orfamide K, extracted as the minor compound produced by *P. protegens* Pf-5. (500MHz, DMF-d7, 298K)

		^{1}H δ [ppm]	^{13}C δ [ppm]
**FA**	CO	-	n.d.
	CH_2α	2.44	43.75
	CHβ	4.04	68.34
	CH_2δ	1.46	26.02
	CH	5.37	149.78
	CH	5.37	149.78
	CH_2	1.52	37.30
	CH_2	1.28	31.82
	CH_2	1.29	29.63
	CH_2	1.28	22.53
	CH_2	2.05	27.12
	CH_3	0.88	13.77
	OH	n.d.	-
**L-Leu1**			
	NH	8.52	-
	CHα	4.33	52.37
	CO	-	n.d.
	CH_2β	1.66	40.19
	CHγ	1.72	24.65
	CH_3δ1	0.90	21.47
	CH_3δ2	0.94	22.80
**D-Glu2**			
	NH	8.65	-
	CHα	4.31	54.28
	CO	-	n.d.
	CH_2β1	2.13	26.79
	CH_2β2	1.99	26.89
	CH_2γ	2.45	30.51
	CO	-	n.d.
	OH	n.d.	-
**D-aThr3**			
	NH	8.28	-
	CHα	4.53	58.46
	CO	-	n.d.
	CHβ	5.22	70.13
	CH_3γ	1.31	17.39
**D-aIle4**			
	NH	7.86	-
	CHα	n.d.	n.d.
	CO	-	n.d.
	CHβ	2.05	36.17
	CH_3γ	0.99	15.23
	CH_2γ	1.17	26.04
	CH_3δ	0.89	11.13

		^{1}H δ [ppm]	^{13}C δ [ppm]
**D-Leu5**			
	NH	n.d.	-
	CHα	4.28	53.36
	CO	-	n.d.
	CH_2β1	1.75	40.12
	CH_2β2	1.53	40.04
	CHγ	1.77	24.57
	CH_3δ1	0.89	22.95
	CH_3δ2	0.82	20.72
**D-Ser6**			
	NH	7.88	-
	CHα	4.37	57.35
	CO	-	n.d.
	CH_2β1	3.87	62.12
	CH_2β2	3.79	62.12
	OH	n.d.	-
**L-Leu7**			
	NH	8.11	-
	CHα	4.38	52.55
	CO	-	n.d.
	CH_2β1	1.74	40.69
	CH_2β2	1.51	40.73
	CHγ	1.77	24.57
	CH_3δ1	0.86	21.03
	CH_3δ2	0.92	22.98
**L-Leu8**			
	NH	7.98	-
	CHα	4.31	52.89
	CO	-	n.d.
	CH_2β1	1.74	40.40
	CH_2β2	1.66	40.41
	CHγ	1.77	24.57
	CH_3δ1	0.89	21.27
	CH_3δ2	0.94	22.80
**D-Ser9**			
	NH	7.67	-
	CHα	4.33	56.64
	CO	-	n.d.
	CH_2β1	3.92	62.20
	CH_2β2	3.80	62.20
	OH	4.99	-
**L-Val10**			
	NH	7.57	-
	CHα	2.18	30.34
	CO	-	n.d.
	CHβ	0.84	17.84
	CH_3γ1	0.90	18.99
	CH_3γ2	2.18	30.34

Table S19: NMR assignment (^{1}H and ^{13}C) of orfamide L, extracted as the minor compound produced by *P. protegens* Pf-5. (500MHz, DMF-d7, 298K)

	^{1}H δ [ppm]	^{13}C δ [ppm]		^{1}H δ [ppm]	^{13}C δ [ppm]
**FA**			**D-Leu5**		
CO	-	n.d.	NH	7.83	-
$CH_2\alpha$	2.44	43.75	CHα	4.28	53.26
CHβ	4.03	68.42	CO	-	n.d.
$CH_2\delta1$	1.47	25.69	$CH_2\beta1$	1.75	40.05
$CH_2\delta2$	1.35	25.69	$CH_2\beta2$	1.51	40.06
CH_2	1.48	37.65	CHγ	1.77	24.53
CH_2	1.28	22.58	$CH_3\delta1$	0.88	23.04
CH_2	1.27	31.91	$CH_3\delta2$	0.80	20.71
CH_2	1.29	29.32			
CH_2	1.28	29.68	**D-Ser6**		
CH_3	0.88	13.74	NH	7.87	-
OH	n.d.	-	CHα	4.41	57.13
			CO	-	n.d.
**L-Leu1**			$CH_2\beta1$	3.84	62.17
NH	8.50	-	$CH_2\beta2$	3.78	62.17
CHα	4.31	52.41	OH	4.81	-
CO	-	n.d.			
$CH_2\beta$	1.65	40.21	**L-Leu7**		
CHγ	1.77	24.53	NH	8.14	-
$CH_3\delta1$	0.94	22.76	CHα	4.39	52.72
$CH_3\delta2$	0.90	21.53	CO	-	n.d.
			$CH_2\beta1$	1.76	40.77
**D-Glu2**			$CH_2\beta2$	1.50	40.76
NH	8.64	-	CHγ	1.77	24.53
CHα	4.3171	54.2525	$CH_3\delta1$	0.86	21.12
CO	-	n.d.	$CH_3\delta2$	0.92	22.96
$CH_2\beta1$	2.1144	26.7709			
$CH_2\beta2$	1.9932	26.7953	**L-Leu8**		
$CH_2\gamma$	2.4490	30.3646	NH	7.95	-
CO	-	n.d.	CHα	4.32	52.86
OH	n.d.	-	CO	-	n.d.
			$CH_2\beta$	1.70	40.41
**D-aThr3**			CHγ	1.77	24.53
NH	8.22	-	$CH_3\delta1$	0.88	21.18
CHα	4.52	58.53	$CH_3\delta2$	0.94	22.76
CO	-	n.d.			
CHβ	5.20	70.20	**D-Ser9**		
$CH_3\gamma$	1.31	17.50	NH	7.69	-
			CHα	4.36	56.44
**D-Val4**			CO	-	n.d.
NH	7.90	-	$CH_2\beta1$	3.90	62.23
CHα	3.97	61.06	$CH_2\beta2$	3.79	62.23
CO	-	n.d.	OH	5.01	-
CHβ	2.20	30.44			
$CH_3\gamma1$	0.99	19.12	**L-Val10**		
$CH_3\gamma2$	0.94	19.06	NH	7.59	-
			CHα	4.57	57.83
			CO	-	n.d.
			CHβ	2.21	29.79
			$CH_3\gamma1$	0.84	17.71
			$CH_3\gamma2$	0.91	19.00

Table S20: NMR assignment (^{1}H and ^{13}C) of orfamide I, extracted as the minor compound produced by *P. protegens* Pf-5. (500MHz, DMF-d7, 298K)

	^{1}H δ [ppm]	^{13}C δ [ppm]		^{1}H δ [ppm]	^{13}C δ [ppm]
**FA**			**D-Leu5**		
CO	-	172.89	NH	7.87	-
$CH_2α$	2.44	43.72	CHα	4.26	53.36
CHβ	4.03	68.41	CO	-	173.15
$CH_2δ1$	1.48	25.68	$CH_2β1$	1.76	39.93
$CH_2δ2$	1.35	25.72	$CH_2β2$	1.53	39.97
CH_2	1.48	37.56	CHγ	1.78	24.57
CH_2	1.28	22.55	$CH_3δ1$	0.82	20.67
CH_2	1.28	31.87	$CH_3δ2$	0.89	22.96
CH_2	1.29	29.64			
CH_3	0.88	13.72	**D-Ser6**		
OH	4.85	-	NH	7.84	-
			CHα	4.39	57.23
**L-Leu1**			CO	-	171.33
NH	8.53	-	$CH_2β1$	3.85	62.05
CHα	4.31	52.34	$CH_2β2$	3.80	62.05
CO	-	173.35	OH	4.85	-
$CH_2β$	1.66	40.08			
CHγ	1.69	24.61	**L-Leu7**		
$CH_3δ1$	0.90	21.42	NH	8.18	-
$CH_3δ2$	0.94	22.78	CHα	4.38	52.58
			CO	-	174.54
**D-Glu2**			$CH_2β1$	1.75	40.66
NH	8.60	-	$CH_2β2$	1.52	40.69
CHα	4.31	54.04	CHγ	1.78	24.57
CO	-	173.35	$CH_3δ1$	0.86	21.02
$CH_2β1$	2.12	26.56	$CH_3δ2$	0.92	22.96
$CH_2β2$	2.01	26.56			
$CH_2γ$	2.49	30.00	**L-Leu8**		
CO	-	173.07	NH	7.98	-
$OCH_3ε$	3.64	51.17	CHα	4.30	52.93
			CO	-	173.35
**D-aThr3**			$CH_2β1$	1.77	40.38
NH	8.27	-	$CH_2β2$	1.65	40.37
CHα	4.53	58.50	CHγ	1.78	24.57
CO	-	171.14	$CH_3δ1$	0.88	21.08
CHβ	5.21	70.11	$CH_3δ2$	0.94	22.78
$CH_3γ$	1.31	17.44			
			**D-Ser9**		
**D-Ile4**			NH	7.64	-
NH	7.84	-	CHα	4.33	56.74
CHα	4.15	59.26	CO	-	170.51
CO	-	172.51	$CH_2β1$	3.93	62.10
CHβ	2.05	36.16	$CH_2β2$	3.81	62.10
$CH_3γ$	0.99	15.21	OH	4.99	-
$CH_2γ$	1.17	26.01			
$CH_3δ$	0.89	11.11	**L-Val10**		
			NH	7.56	-
			CHα	4.49	57.91
			CO	-	170.99
			CHβ	2.19	30.29
			$CH_3γ1$	0.90	19.00
			$CH_3γ2$	0.84	17.80

Table S21: NMR assignment (^{1}H and ^{13}C) of orfamide L, extracted as the minor compound produced by *P. protegens* Pf-5. (500MHz, DMF-d7, 298K)

	^1H δ [ppm]	^{13}C δ [ppm]
**FA**		
CO	-	n.d.
CH_2α	2.44	43.75
CHβ	4.03	68.40
CH_2δ1	1.48	25.75
CH_2δ2	1.34	24.77
CH_2	1.47	37.59
CH_2	1.28	22.48
CH_2	1.28	31.87
CH_2	1.29	29.58
CH_3	0.86	13.71
OH	4.82	-
**L-Leu1**		
NH	8.48	-
CHα	4.32	52.69
CO	-	n.d.
CH_2β	1.65	40.22
CHγ	1.67	24.45
CH_3δ1	0.90	21.41
CH_3δ2	0.95	22.80
**D-Glu2**		
NH	8.59	-
CHα	4.33	54.09
CO	-	n.d.
CH_2β1	2.10	26.53
CH_2β2	1.97	26.81
CH_2γ	2.45	30.17
CO	-	n.d.
OH	n.d.	-
**D-aThr3**		
NH	8.18	-
CHα	4.52	58.44
CO	-	n.d.
CHβ	5.21	70.82
CH_3γ	1.31	17.37
**D-aIle4**		
NH	7.77	-
CHα	4.21	58.96
CO	-	n.d.
CHβ	2.05	36.37
CH_3γ	0.99	15.03
CH_2γ	n.d.	n.d.
CH_3δ	0.89	11.19

	^1H δ [ppm]	^{13}C δ [ppm]
**D-Leu5**		
NH	7.96	-
CHα	4.24	53.57
CO	-	n.d.
CH_2β	1.54	39.90
CHγ	1.77	24.56
CH_3δ1	0.90	22.92
CH_3δ2	0.82	20.78
**D-Ser6**		
NH	7.84	-
CHα	4.36	57.16
CO	-	n.d.
CH_2β1	3.86	61.90
CH_2β2	3.81	61.88
OH	4.88	-
**L-Leu7**		
NH	8.18	-
CHα	4.37	52.76
CO	-	n.d.
CH_2β	1.57	40.82
CHγ	1.75	24.53
CH_3δ1	0.87	20.96
CH_3δ2	0.92	22.87
**L-Leu8**		
NH	7.87	-
CHα	4.32	52.69
CO	-	n.d.
CH_2β	1.69	40.43
CHγ	1.81	24.61
CH_3δ1	0.87	20.96
CH_3δ2	0.95	22.80
**D-Ser9**		
NH	7.48	-
CHα	4.32	56.77
CO	-	n.d.
CH_2β1	3.94	62.18
CH_2β2	3.82	62.21
OH	5.02	-
**L-Ile10**		
NH	7.56	-
CHα	4.59	56.76
CO	-	n.d.
CHβ	1.98	36.61
CH_3γ	0.89	15.48
CH_2γ	n.d.	n.d.
CH_3δ	0.84	10.90

Table S22: NMR assignment (^{1}H and ^{13}C) of orfamide M, extracted as the minor compound produced by *P. protegens* Pf-5. (500MHz, DMF-d7, 298K)

		^{1}H δ [ppm]	^{13}C δ [ppm]			^{1}H δ [ppm]	^{13}C δ [ppm]
**FA**				**D-Leu5**			
	CO	-	n.d.		NH	n.d.	-
	CH_2α	2.43	43.76		CHα	4.28	53.38
	CHβ	4.03	68.36		CO	-	n.d.
	CH_2δ	1.47	25.78		CH_2β1	1.76	40.29
	CH_2	1.48	37.65		CH_2β2	1.52	40.30
	CH_2	1.28	22.52		CHγ	1.76	24.62
	CH_2	1.28	22.52		CH_3δ1	0.82	20.75
	CH_2	1.28	22.52		CH_3δ2	0.89	22.95
	CH_2	1.28	22.52				
	CH_2	1.28	22.52	**D-Ser6**			
	CH_2	1.28	22.52		NH	7.89	-
	CH_2	1.28	31.90		CHα	4.37	57.31
	CH_2	1.29	29.61		CO	-	n.d.
	CH_3	0.88	14.26		CH_2β1	3.86	62.07
	OH	n.d.	-		CH_2β2	3.79	62.06
					OH	n.d.	-
**L-Leu1**							
	NH	8.49	-	**L-Leu7**			
	CHα	4.34	52.62		NH	8.10	-
	CO	-	n.d.		CHα	4.38	52.55
	CH_2β1	1.73	40.29		CO	-	n.d.
	CH_2β2	1.65	40.29		CH_2β1	1.74	40.66
	CHγ	1.76	24.62		CH_2β2	1.51	40.66
	CH_3δ1	0.90	21.43		CHγ	1.76	24.62
	CH_3δ2	0.94	22.83		CH_3δ1	0.85	21.03
					CH_3δ2	0.92	22.93
**D-Glu2**							
	NH	8.65	-	**L-Leu8**			
	CHα	4.34	54.18		NH	7.96	-
	CO	-	n.d.		CHα	4.34	52.62
	CH_2β	2.10	26.54		CO	-	n.d.
	CH_2γ	n.d.	n.d.		CH_2β1	1.74	40.54
	CO	-	n.d.		CH_2β2	1.66	40.51
	OH	n.d.	-		CHγ	1.76	24.62
					CH_3δ1	0.94	22.83
**D-aThr3**					CH_3δ2	0.87	21.10
	NH	8.22	-				
	CHα	4.54	58.31	**D-Ser9**			
	CO	-	n.d.		NH	7.66	-
	CHβ	5.21	70.81		CHα	4.34	56.74
	CH_3γ	1.31	17.35		CO	-	n.d.
					CH_2β1	3.79	62.19
**D-aIle4**					CH_2β2	3.89	62.19
	NH	7.84	-		OH	4.99	-
	CHα	4.14	59.47				
	CO	-	n.d.	**L-Val10**			
	CHβ	2.05	36.27		NH	7.58	-
	CH_3γ	0.98	15.18		CHα	4.49	58.00
	CH_2γ	n.d.	n.d.		CO	-	n.d.
	CH_3δ	0.89	11.10		CHβ	2.19	30.40
					CH_3γ1	0.85	17.82
					CH_3γ2	0.91	18.99

NMR fingerprint matching with orfamide B from *Pseudomonas* sp. F6

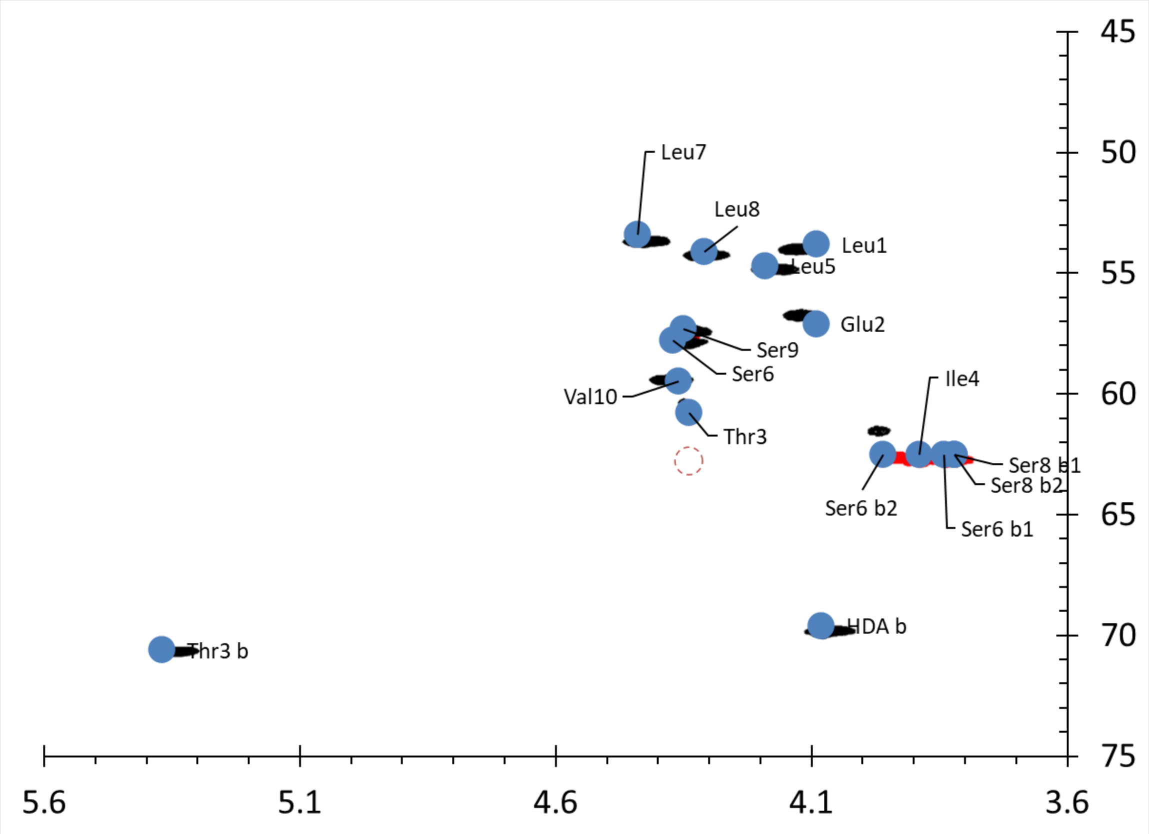

Figure S43: Comparison of the ^{1}H-{^{13}C} HSQC (CH)α fingerprint of the natural compound extracted from *P. aestus* CMR5c with the tabulated ^{1}H and ^{13}C chemical shifts of orfamide B from *Pseudomonas* sp. F6, all recorded in MeOH-d3 at 500 MHz and 298K. (4)

Analysis of the natural orfamide B from *Pseudomonas sessilinigenes* CMR12a

Extraction and purification of natural CLiP

After growth of *P. sessilinigenes* CMR12a, the cyclic lipopeptides produced by this bacterial strain was extracted from the supernatant and cell content. A single fraction was collected and examined by mass analysis. The main compound eluted at a retention time of 12.2 min. This peptide fraction was purified via semi-preparative RP- HPLC analysis, using a two solvent system: H_2O + 0.1% TFA (A) and CH_3CN (B), via application of a linear gradient over 15 minutes going from 25:75 to 0:100 (A:B).

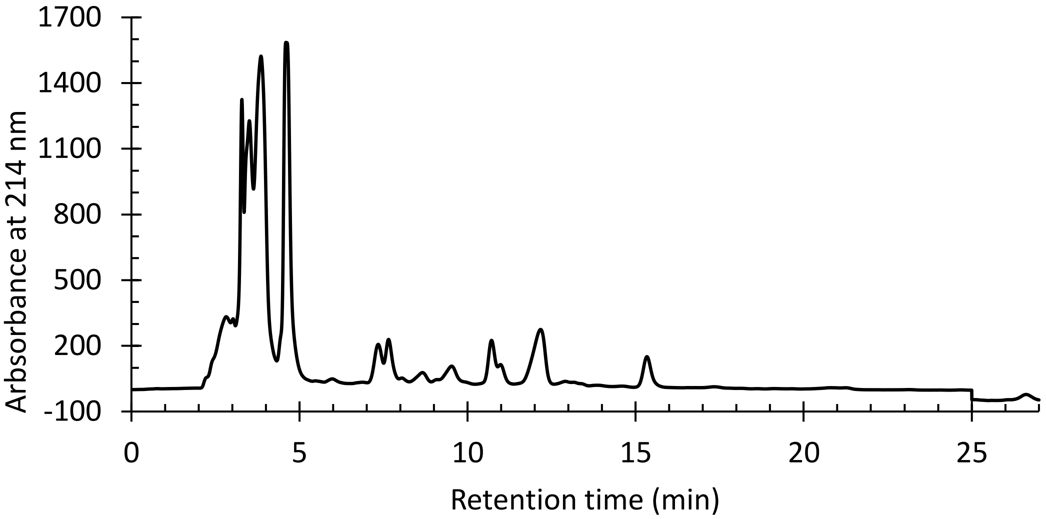

Figure S44: Semi-preparative reversed-phase HPLC chromatogram of *P. sessilinigenes* CMR12a, showing the presence of orfamide B at 12.2 minutes.

NMR characterization of orfamides extracted from *P. sessilinigenes* CMR12a

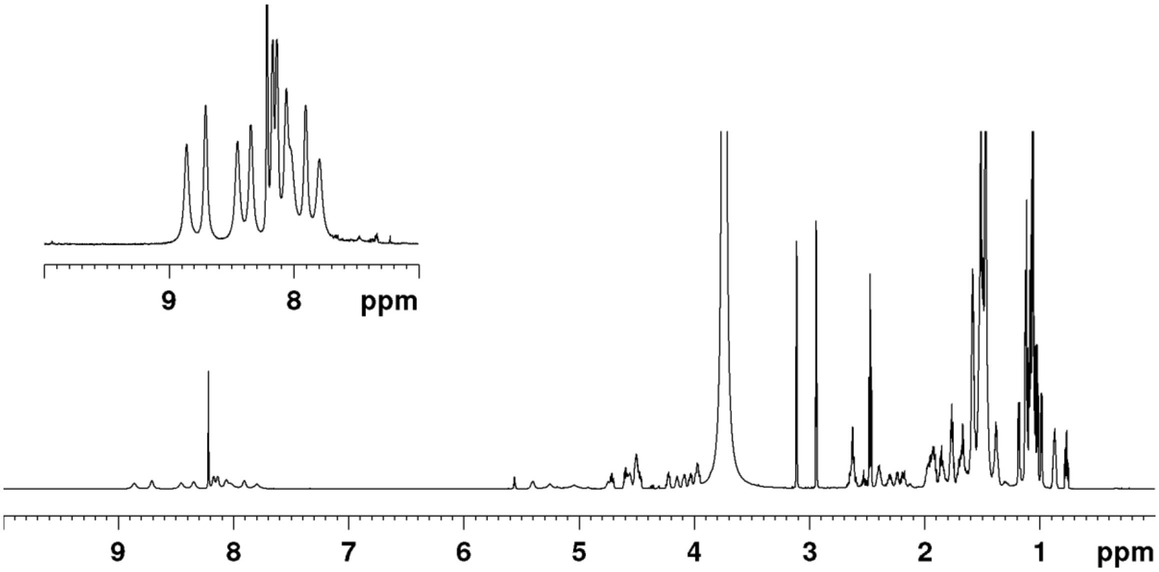

Figure S45: 1D ^{1}H spectrum of the orfamide B as extracted from *P. sessilinigenes* CMR12a. (DMF-d7, 298K, 500 MHz)

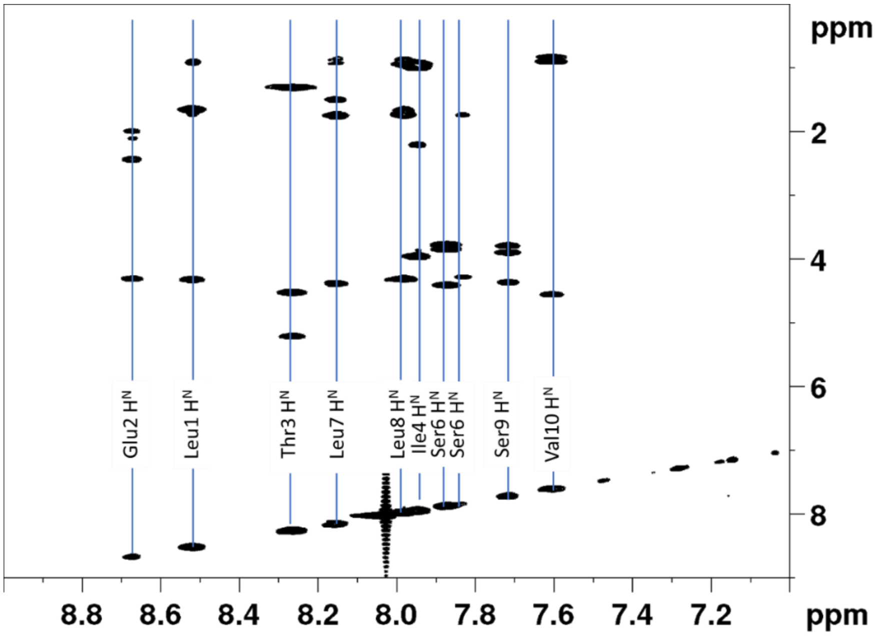

Figure S46: ^{1}H-^{1}H TOCSY traces of the individual amino acids in orfamide B as isolated from *P. sessilinigenes* CMR12a.(500MHz, DMF-*d7*, 298K)

Table S23: NMR assignment (^{1}H and ^{13}C) of orfamide B, extracted as the main compound produced by *P. sessilinigenes* CMR12a. (500MHz, DMF-d7, 298K)

		^{1}H δ [ppm]	^{13}C δ [ppm]			^{1}H δ [ppm]	^{13}C δ [ppm]
**FA**				**D-Leu5**			
	CO	-	173.09		NH	7.83	4.28
	CH_2α	2.4335	43.72		CHα	4.2749	53.20
	CHβ	4.03	68.36		CO	-	n.d.
	CH_2γ	1.47	37.61		CH_2β1	1.73	40.04
	CH_2δ1	1.47	25.64		CH_2β2	1.51	39.95
	CH_2δ2	1.3312	25.63		CHγ	1.79	24.46
	CH_2ε	1.32	29.31		CH_3δ1	0.87	23.01
	CH_2ζ	1.32	29.31		CH_3δ2	0.80	20.68
	CH_2η	1.32	29.31	**D-Ser6**			
	CH_2θ	1.32	29.31		NH	7.87	-
	CH_2ι	1.32	29.31		CHα	4.40	57.09
	CH_2κ	1.32	29.31		CO	-	n.d.
	CH_2λ	1.27	31.85		CH_2β1	3.83	62.09
	CH2µ	1.29	22.49		CH_2β2	3.78	62.09
	CH_3ν	0.87	13.71		OHγ	7.87	-
	OH	n.d.	-	**L-Leu7**			
**L-Leu1**					NH	8.15	-
	NH	8.52	-		CHα	4.38	52.66
	CHα	4.32	52.33		CO	-	n.d.
	CO	-	n.d.		CH_2β1	1.73	40.65
	CH_2β1	1.66	40.21		CH_2β2	1.50	40.72
	CH_2β2	1.63	40.21		CHγ	1.78	24.55
	CHγ	1.73	24.60		CH_3δ1	0.92	22.85
	CH_3δ1	0.94	22.70		CH_3δ2	0.86	21.05
	CH_3δ2	0.89	21.46	**L-Leu8**			
**D-Glu2**					NH	7.98	-
	NH	8.67	-		CHα	4.31	52.87
	CHα	4.30	54.30		CO	-	n.d.
	CO	-	n.d.		CH_2β1	1.73	40.37
	CH_2β1	2.11	26.75		CH_2β2	1.67	40.37
	CH_2β2	1.99	26.75		CHγ	1.75	24.60
	CH_2γ	2.44	30.53		CH_3δ1	0.93	22.69
	COδ	-	174.46		CH_3δ2	0.87	21.19
	OH	n.d.	-	**D-Ser9**			
**D-aThr3**					NH	7.71	-
	NH	8.26	-		CHα	4.36	56.41
	CHα	4.52	58.52		CO	-	n.d.
	CO	-	171.20		CH_2β1	3.90	62.19
	CHβ	5.20	70.18		CH_2β2	3.79	62.19
	CH_3γ	1.31	17.37		OHγ	7.72	-
**D-Val4**				**L-Val10**			
	NH	7.95	-		NH	7.61	-
	CHα	3.95	60.99		CHα	4.56	57.82
	CO	-	172.50		CO	-	n.d.
	CHβ	2.20	29.71		CHβ	2.20	30.39
	CH_3γ1	0.99	19.09		CH_2γ1	0.90	18.95
	CH_3γ2	0.93	19.03		CH_2γ2	0.83	17.68

Analysis of the natural orfamide A from *Pseudomonas* sp. PH1b

Extraction and purification of natural CLiP

After growth of *P.* sp. PH1b, the cyclic lipopeptides produced by this bacterial strain was extracted from the supernatant and cell content. In total 8 fractions were collected and examined by mass analysis. The main compound eluted at a retention time of 20.6 min in Fig S1. This peptide fraction was purified via semi-preparative RP- HPLC analysis, using a two solvent system: H_2O + 01% TFA (A) and CH_3CN (B), via application of a linear gradient over 15 minutes going from 25:75 to 0:100 (A:B).

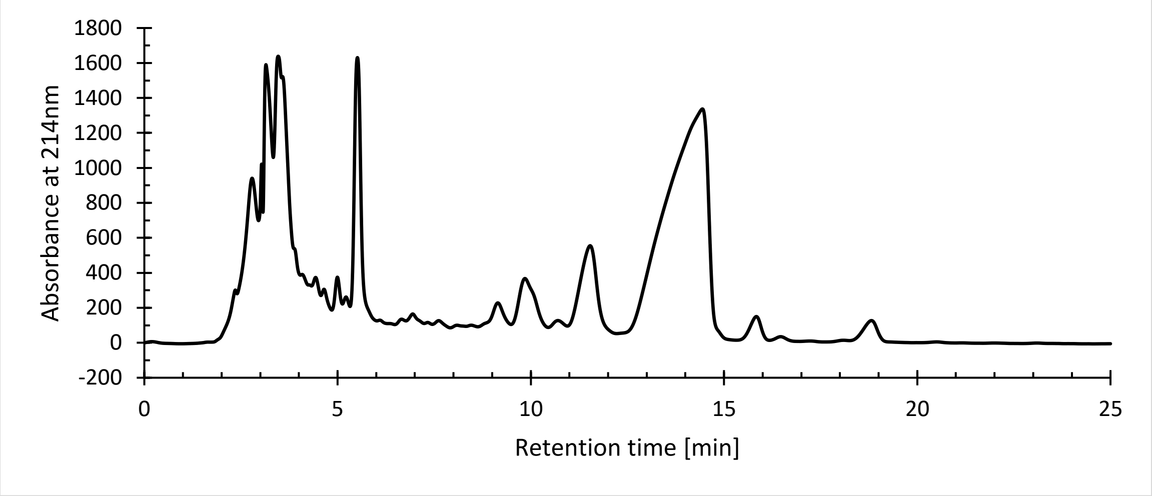

Figure S47: Semi-preparative reversed-phase HPLC chromatogram of *Pseudomonas* sp. PH1b, showing the presence of orfamide A at 14.5 minutes.

NMR characterization of orfamides extracted from *Pseudomonas* sp. PH1b

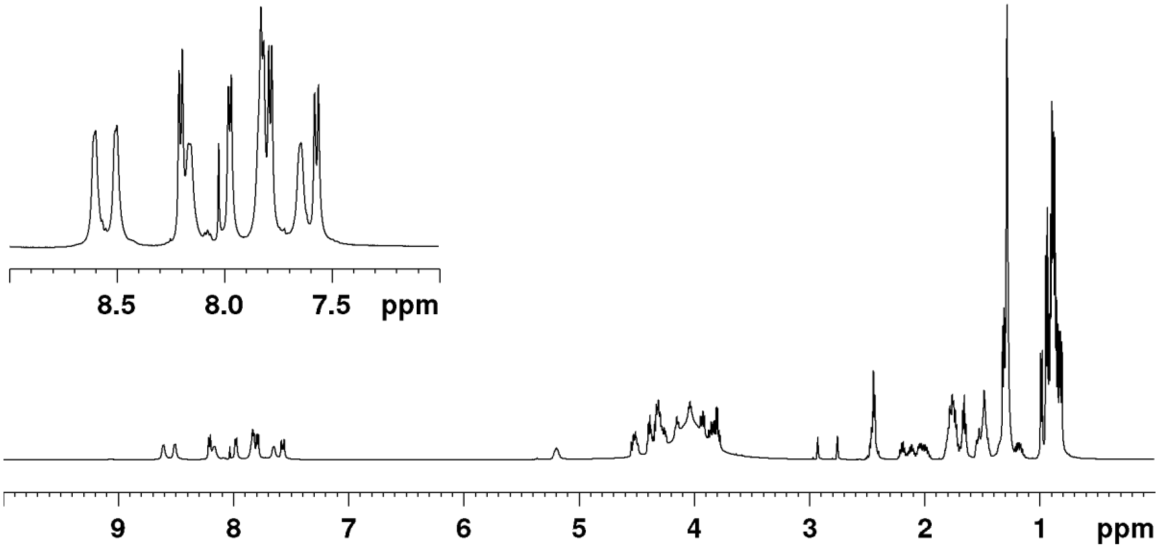

Figure S48: 1D ^{1}H spectrum of the orfamide A as extracted from *Pseudomonas* sp. PH1b. (DMF-d7, 298K, 500 MHz)

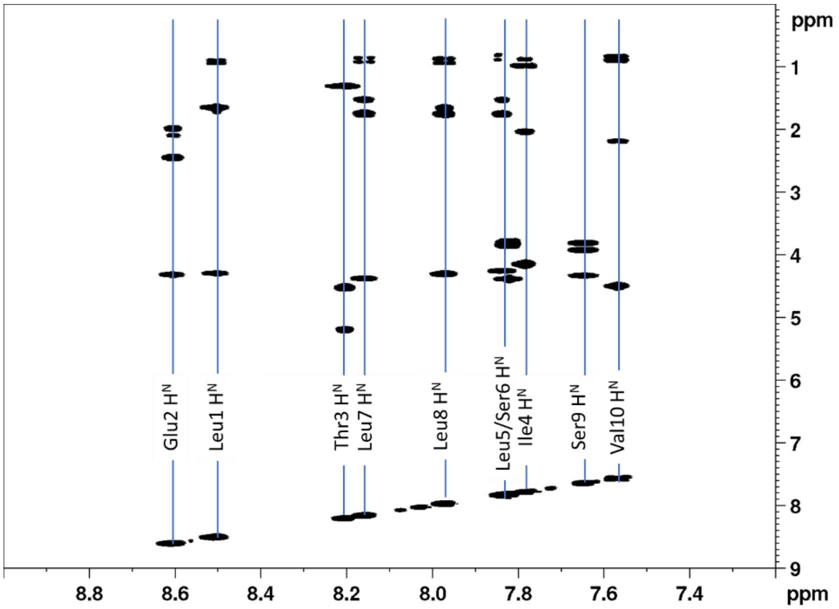

Figure S49: ^{1}H-^{1}H TOCSY traces of the individual amino acids in orfamide A as isolated from *Pseudomonas* sp. PH1b.(500MHz, DMF-*d7*, 298K)

Table S24: NMR assignment (^{1}H and ^{13}C) of orfamide A, extracted as the main compound produced by *Pseudomonas* sp. PH1b. (500MHz, DMF-d7, 298K)

	^{1}H δ [ppm]	^{13}C δ [ppm]		^{1}H δ [ppm]	^{13}C δ [ppm]
**FA**			**D-Leu5**		
CO	-	173.00	NH	7.84	-
CH$_2$α	2.43	53.53	CHα	4.25	53.16
CHβ	4.03	68.15	CO	-	173.53
CH$_2$γ	1.47	37.41	CH$_2$β1	1.75	39.78
CH$_2$δ1	1.47	25.43	CH$_2$β2	1.52	39.79
CH$_2$δ2	1.34	25.43	CHγ	1.80	24.31
CH$_2$ε	1.27	29.30	CH$_3$δ1	0.89	22.81
CH$_2$ζ	1.27	29.30	CH$_3$δ2	0.81	20.54
CH$_2$η	1.27	29.30			
CH$_2$θ	1.27	29.30	**D-Ser6**		
CH$_2$ι	1.27	29.30	NH	7.82	-
CH$_2$κ	1.27	29.30	CHα	4.38	56.97
CH$_2$λ	1.26	31.72	CO	-	171.46
CH2μ	1.28	22.33	CH$_2$β1	3.85	61.85
CH$_3$ν	0.87	13.58	CH$_2$β2	3.79	61.85
OH	n.d.	-	OH	n.d.	-
**L-Leu1**			**L-Leu7**		
NH	8.51	-	NH	8.16	-
CHα	4.29	52.28	CHα	4.38	52.51
CO	-	n.d.	CO	-	173.26
CH$_2$β	1.64	39.90	CH$_2$β1	1.75	40.56
CHγ	1.69	24.44	CH$_2$β2	1.51	40.56
CH$_3$δ1	0.94	22.58	CHγ	1.77	24.37
CH$_3$δ2	0.90	21.30	CH$_3$δ1	0.92	22.77
			CH$_3$δ2	0.86	20.86
**D-Glu2**					
NH	8.61	-	**L-Leu8**		
CHα	4.31	53.98	NH	7.97	-
CO	-	173.44	CHα	4.30	52.73
CH$_2$β1	2.11	26.49	CO	-	n.d.
CH$_2$β2	1.98	26.49	CH$_2$β1	1.76	40.23
CH$_2$γ	2.44	30.01	CH$_2$β2	1.66	40.23
CO	-	174.30	CHγ	1.74	24.48
OH	n.d.	-	CH$_3$δ1	0.94	22.60
			CH$_3$δ2	0.88	20.97
**D-aThr3**					
NH	8.20	-	**D-Ser9**		
CHα	4.52	58.35	NH		
CO	-	171.06	CHα	4.33	56.52
CHβ	5.19	69.89	CO	-	170.87
CH$_3$γ	1.31	17.23	CH$_2$β1	3.92	61.94
			CH$_2$β2	3.80	61.94
**D-aIle4**			OH	n.d.	-
NH	7.78	-			
CHα	4.15	59.08	**L-Val10**		
CO	-	172.80	NH	7.57	-
CHβ	2.03	36.03	CHα	4.49	57.71
CH$_2$γ1	1.47	25.81	CO	-	n.d.
CH$_2$γ2	1.17	25.81	CHβ	2.18	30.09
CH3γ	0.98	15.03	CH$_3$γ1	0.90	18.84
CH$_3$δ	0.88	10.95	CH$_3$γ2	0.84	17.60

Table S25: NMR assignment (^{1}H and ^{13}C) of orfamide B, extracted as the minor compound produced by *Pseudomonas* sp. PH1b. (500MHz, DMF-d7, 298K)

	^{1}H δ [ppm]	^{13}C δ [ppm]		^{1}H δ [ppm]	^{13}C δ [ppm]
**FA**			**D-Leu5**		
CO	-	n.d.	NH	7.77	-
$CH_2α$	2.44	43.70	CHα	4.27	53.11
CHβ	4.04	68.33	CO	-	n.d.
$CH_2γ$	1.48	37.55	$CH_2β1$	1.74	39.94
$CH_2δ1$	1.48	25.63	$CH_2β2$	1.51	39.94
$CH_2δ2$	1.34	25.63	CHγ	1.80	24.41
$CH_2ε$	1.29	29.63	$CH_3δ1$	0.88	22.96
$CH_2ζ$	1.29	29.63	$CH_3δ2$	0.80	20.61
$CH_2η$	1.29	29.63	**D-Ser6**		
$CH_2θ$	1.29	29.63	NH	7.84	-
$CH_2ι$	1.29	29.63	CHα	4.41	57.00
$CH_2κ$	1.29	29.63	CO	-	n.d.
$CH_2λ$	1.27	31.81	$CH_2β1$	3.85	62.11
CH2μ	1.29	22.44	$CH_2β2$	3.78	62.11
$CH_3ν$	0.88	13.66	OHγ	4.78	-
OH			**L-Leu7**		
**L-Leu1**			NH	8.15	-
NH	8.52	-	CHα	4.39	52.62
CHα	4.31	52.32	CO	-	173.29
CO	-	n.d.	$CH_2β1$	1.75	40.72
$CH_2β1$	1.67	40.12	$CH_2β2$	1.50	40.72
$CH_2β2$	1.64	40.12	CHγ	1.78	24.52
CHγ	1.73	24.56	$CH_3δ1$	0.93	22.91
$CH_3δ1$	0.94	22.65	$CH_3δ2$	0.86	21.01
$CH_3δ2$			**L-Leu8**		
**D-Glu2**			NH	7.96	-
NH	8.64	-	CHα	4.31	52.81
CHα	4.31	54.23	CO	-	n.d.
CO	-	n.d.	$CH_2β1$	1.73	40.34
$CH_2β1$	2.12	26.60	$CH_2β2$	1.67	40.34
$CH_2β2$	2.00	26.60	CHγ	1.75	24.59
$CH_2γ$	2.46	30.17	$CH_3δ1$	0.94	22.63
COδ	-	174.28	$CH_3δ2$	0.88	21.08
OH			**D-Ser9**		
**D-aThr3**			NH	7.70	-
NH	8.23	-	CHα	4.35	56.40
CHα	4.51	58.52	CO	-	n.d.
CO	-	171.20	$CH_2β1$	3.90	62.15
CHβ	5.21	70.01	$CH_2β2$	3.80	62.15
$CH_3γ$	1.30	17.46	OHγ	7.70	-
**D-Val4**			**L-Val10**		
NH	7.90	-	NH	7.58	-
CHα	3.96	61.00	CHα	4.57	57.74
CO	-	172.44	CO	-	n.d.
CHβ	2.20	29.55	CHβ	2.21	30.36
$CH_3γ1$	1.00	19.04	$CH_2γ1$	0.90	18.88
$CH_3γ2$	0.94	18.99	$CH_2γ2$	0.83	17.62

Table S26: NMR assignment (^{1}H and ^{13}C) of orfamide A, extracted as the main compound produced by *Pseudomonas* sp. PH1b. (500MHz, methanol-d3, 298K)

		^{1}H δ [ppm]	^{13}C δ [ppm]			^{1}H δ [ppm]	^{13}C δ [ppm]
**FA**				**D-Leu5**			
	CO	-	n.d.		NH	7.83	-
	CH$_2$α	2.41	44.31		CHα	4.18	54.84
	CHβ	4.07	69.82		CO	-	n.d.
	CH$_2$γ	1.49	38.36		CH$_2$β1	1.76	40.67
	CH$_2$δ1	1.48	26.45		CH$_2$β2	1.55	40.66
	CH$_2$δ2	1.32	26.45		CHγ	1.76	25.50
	CH$_2$ε	1.28	30.41		CH$_3$δ1	0.90	23.46
	CH$_2$ζ	1.28	30.41		CH$_3$δ2	0.85	21.00
	CH$_2$η	1.28	30.41	**D-Ser6**			
	CH$_2$θ	1.28	30.41		NH	7.91	-
	CH$_2$ι	1.28	30.41		CHα	4.35	57.89
	CH$_2$κ	1.28	30.41		CO	-	n.d.
	CH$_2$λ	1.27	32.88		CH$_2$β1	3.89	62.80
	CH2μ	1.29	23.48		CH$_2$β2	3.82	62.80
	CH$_3$ν	0.89	14.23		OHγ	n.d.	-
	OH	n.d.	-	**L-Leu7**			
**L-Leu1**					NH	8.14	-
	NH	8.58	-		CHα	4.42	53.66
	CHα	4.12	54.02		CO	-	n.d.
	CO	-	n.d.		CH$_2$β1	1.77	41.41
	CH$_2$β1	1.66	40.43		CH$_2$β2	1.52	41.43
	CH$_2$β2	1.63	40.43		CHγ	1.76	25.50
	CHγ	1.66	25.63		CH$_3$δ1	0.96	23.50
	CH$_3$δ1	0.98	23.06		CH$_3$δ2	0.91	21.32
	CH$_3$δ2	0.93	22.18	**L-Leu8**			
**D-Glu2**					NH	7.97	-
	NH	8.97	-		CHα	4.31	54.25
	CHα	4.12	56.74		CO	-	n.d.
	CO	-	n.d.		CH$_2$β1	1.74	41.13
	CH$_2$β1	2.10	27.77		CH$_2$β2	1.66	41.13
	CH$_2$β2	1.99	27.77		CHγ	1.72	25.69
	CH$_2$γ	2.36	33.60		CH$_3$δ1	0.97	23.16
	COδ	-	n.d.		CH$_3$δ2	0.91	21.76
	OH	n.d.	-	**D-Ser9**			
**D-aThr3**					NH	7.73	-
	NH	8.30	-		CHα	4.33	57.45
	CHα	4.35	60.38		CO	-	n.d.
	CO	-	n.d.		CH$_2$β1	3.94	62.46
	CHβ	5.34	70.65		CH$_2$β2	3.87	62.46
	CH$_3$γ	1.36	18.28		OHγ	n.d.	-
**D-alle4**				**L-Val10**			
	NH	7.62	-		NH	7.80	-
	CHα	3.97	61.54		CHα	4.38	59.42
	CO	-	n.d.		CO	-	n.d.
	CHβ	2.00	36.93		CHβ	2.14	31.32
	CH$_2$γ1	1.46	26.80		CH$_2$γ1	0.89	19.56
	CH$_2$γ2	1.17	26.80		CH$_2$γ2	0.84	18.66
	CH3γ	0.97	16.17				
	CH$_3$δ	0.90	11.51				

Stereochemical analysis of orfamide A, excreted by *P. protegens* Pf-5

0,83 mg of orfamide A was hydrolyzed during 22,5 hours using a 6M HCl solution at 90°C. Consecutively, the mixture was dried using a gentle N2 stream while keeping the temperature at 60°C to enhance evaporation. Next, 100µL of a 1M $NaHCO_3$ solution was added to the dried, crude mixture to quench the reaction. Subsequently, 150µL of a 1% (m/m%) solution of Marfey's reagent (1-fluoro-2-4-dinitrophenyl-5-L-alanine amide) in HPLC grade acetone (VWR) was added. The solution was transferred to an Eppendorf and was let to react in a Thermoshaker (1100 r.p.m, 40°C) for approximately an hour. Thereafter, 50µL of a 2M HCl solution was used to quench the nucleophilic aromatic substitution reaction and the solution was dried using a SpeedVac (Thermofisher). The resulting dried substance was redissolved in 200µL DMSO of which 30 µL was taken out to prepare an HPLC sample. The 30µL was diluted using a mixture (40/60-acetonitrile/5mM ammonium acetate). The solvents used for the measure of samples are 0.1% TFA in H_2O (A) and MeCN (B) at a flow rate of 3 mL min^{-1}. After sample injection, the column was flushed with 100% A for 3 min, followed by an isothermal (50°C) gradient from 0 to 100 % B over 120 min and subsequent flushing with 100 % B for 3 min. The detector was set to 340 nm to selectively monitor the derivatives.

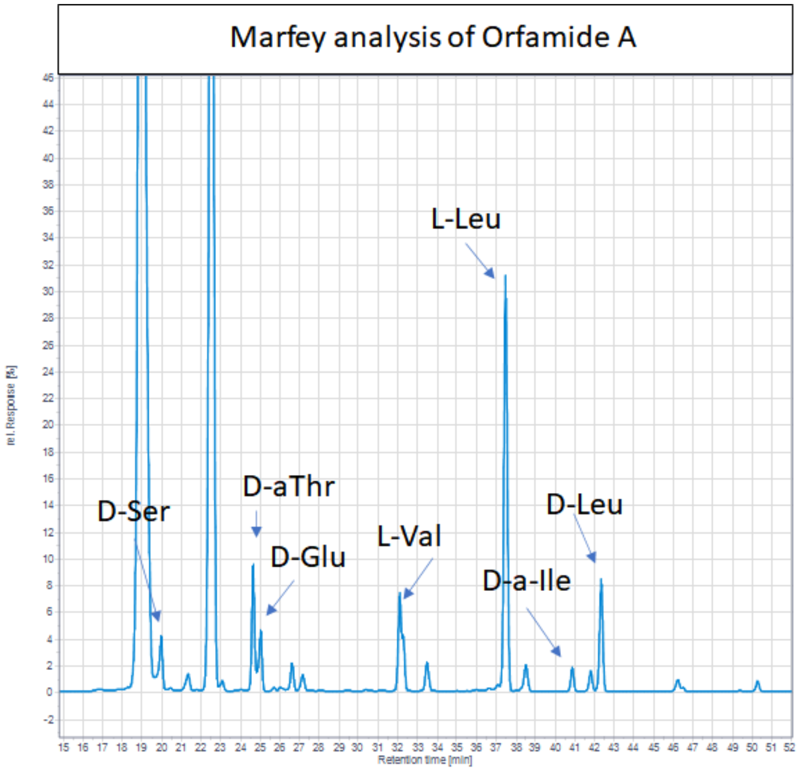

Figure S50: Chromatogram obtained by derivatization of the individual amino acid present in orfamide A. Conditions are highlighted above.

Table **S**27: Summary of the found A.A. enantiomeric ratios from orfamide A.

CALCULATED AT 340 NM	SER		GLX		VAL		LEU		THR		ILE	
CONFIGURATION	L	D	L	D	L	D	L	D	L	D-allo	L	D-allo
RETENTION TIME (MIN)	19.989	20.063	21.795	25.004	32.066	37.226	37.626	42.434	22.154	22.824	36.819	40.839
HEIGHT	/	481	1.84	104.724	1632	/	712	192.2	/	216.8	3.96	41.908
RATIO	/	/	0	1	1	0	3	1	0	1	0	1

Analysis of xantholysin A from *Pseudomonas mosselii* BW11M1

Extraction and purification of natural CLiP

Extraction and purification of xantholysin A was performed as previously reported (8).

Stereochemical analysis of xantholysin A, excreted by *P. mosselii* BW11M1

1.3 mg of xantholysin A was dissolved in 10ml 6N HCl and the solution was let to react for 24h at 95°C. Afterwards the crude mixture was dried using a gentle Ar-flow while simultaneously applying heat (50°C) to enchance evaporation. 200µL of a 1M $NaHCO_3$ solution was added and 360 µL of a 1% FDAA in acetone solution was added. The derivatization step was let to proceed during one hour (40°C). Quenching was done using 80µL of a 2M HCl solution and was let to dry. The resulting dried substance was redissolved in 200µL DMSO of which 30 µL was taken out to prepare an HPLC sample. The 30µL was diluted using a mixture (40/60- acetonitrile/5mM Ammoniumacetate). The solvents used for the measure of samples are 0.1% TFA in H_2O (A) and MeCN (B) at a flow rate of 3 mL min^{-1}. After sample injection, the column was flushed with 100% A for 3 min, followed by an isothermal (50°C) gradient from 0 to 100 % B over 120 min and subsequent flushing with 100 % B for 3 min. The detector was set to 340 nm to selectively monitor the derivatives.

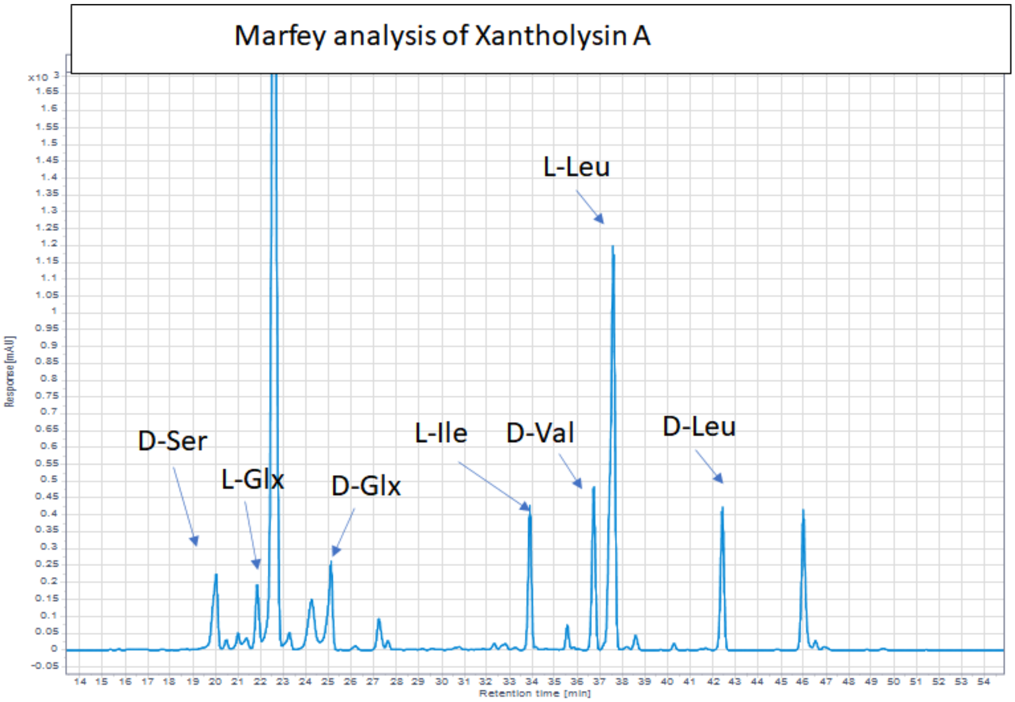

Figure S51: Chromatogram obtained by derivatization of the individual amino acid present in Xantholysin A. Conditions are highlighted above.

Table S 28: Summary of the found A.A. enantiomeric ratios from xantholysin A.

	Ratios Marfey of XantolysinA									
**Calculated at 340 nm**	Ser		Glx		Val		Leu		Ile	
**Configuration**	L	D	L	D	L	D	L	D	L	D-allo
**Retention Time (min)**	19,94	20.063	23.233	25.004	32.33	37.567	37.567	42.434	33.819	40.839
**Height**	/	223	52	263	493	17	425	706	484	/
**Ratio**	0	2	1	3	1	0	2	3	1	0

Multidisciplinary data of xantholysin A from *P. mosselii* BW11M1

We first retrieved the previously available data from both the bioinformatic and the chemical analysis workflows from Li *et al* (ref) and expanded this with configurational analysis of the major xantholysin A compound of *P. mosselii* BW11M1. Marfey's analysis yielded 3x L-Leu, 2x D-Leu, 1x L-Glx, 4x D-Glx, 2x D-Val, 1x D-Ser and 1x L-Ile, where Glx indicates either Gln or Glu since these cannot be distinguished using Marfey's protocol. This result is identical to what is described by Molina-Santiago et al (Molina-Santiogo, MB, 2015) and what can be expected from the revised structure of MA026. Given their configurational homogeneity, the configurations of D-Val4, D-Ser6, D-Val8 and L-Ile14 are unambiguously established. C-domain analysis, as described by Li *et al.* (Li), proposes 5 D-Glx, 2 L-Leu and 3 D-Leu (Table 3). Consequently, there is a mismatch between the prediction and Marfey's analysis both for the leucines and for the glutamines/glutamic acid. Nevertheless, the occurrence of $^{L}C_{L}$ domains in module 12 and 13 of the NRPS assembly line allow to designate Leu11 and Leu12 as L-configured, reducing the positional ambiguity of the remaining L-Leu and both D-Leu to three possibilities. As for the five Glx residues, five different distributions of the only L-Glx residue are possible. Since the positional ambiguity of the leucines is independent from that of the Glx residues, 15 different sequences should be considered at this point. Given the considerable expenditure required to synthesize 15 different sequences, prioritization is in order based on all available information. Considering the proposed structural similarity between xantholysin A and MA026, it appeared evident to synthesize this compound using the same total synthesis approach applied for bananamide and orfamide, as described in detail in the supplementary materials section. As is clearly demonstrated by the superposition of the α(C-H) spectral fingerprint of the purified synthetic (14:8)L1L6 compound, equivalent to revised MA026, with that of xantholysin from *P. mosselii* BMW11M1, a perfect match is obtained. This unequivocally confirms that MA026 from *P*. sp. RtlB026 and xantholysin A from *P. mosselii* BW11M1 are in fact identical. As this establishes Leu1 and Gln6 as L-amino acids, modules 2 of XtlA and module 7 of XltB in the NRPS of *P. mosselii* BW11M1 are hereby shown to have epi-inactive C/E domains incapable of epimerizing the amino acid at the preceding position.

Table S29: Sequence, configurational analysis, and assignment of xantholysin A from *P. mosselii* BW11M1

P. putida **BW11M1**	**AA1**	**AA2**	**AA3**	**AA4**	**AA5**	**AA6**	**AA7**	**AA8**	**AA9**	**AA10**	**AA11**	**AA12**	**AA13**	**AA14**
**Biological workflow**														
**A domain**	Leu	Glu	Gln	Val	Leu	Gln	Ser	Val	Leu	Gln	Leu	Leu	Gln	Ile
**C domain**	Cstart	C/E	C/E	C/E	C/E	C/E	C/E	C/E	C/E	C/E	C/E	$^{L}C_{L}$	$^{L}C_{L}$	C/E
**Prediction**	D	D	D	D	D	D	D	D	D	D	L	L	D	L
**Chemical workflow**														
**NMR analysis**	Leu	Glu	Gln	Val	Leu	Gln	Ser	Val	Leu	Gln	Leu	Leu	Gln	Ile
**Marfey's analysis**	L/D	L/D	L/D	D	L/D	L/D	D	D	L/D	L/D	L/D	L/D	L/D	L
**Published revised sequence MA026**														
**Sequence**	Leu	Glu	Gln	Val	Leu	Gln	Ser	Val	Leu	Gln	Leu	Leu	Gln	Ile
**Stereochemistry**	L	D	D	D	D	L	D	D	D	D	L	L	D	L

Synthesis of xantholysin A

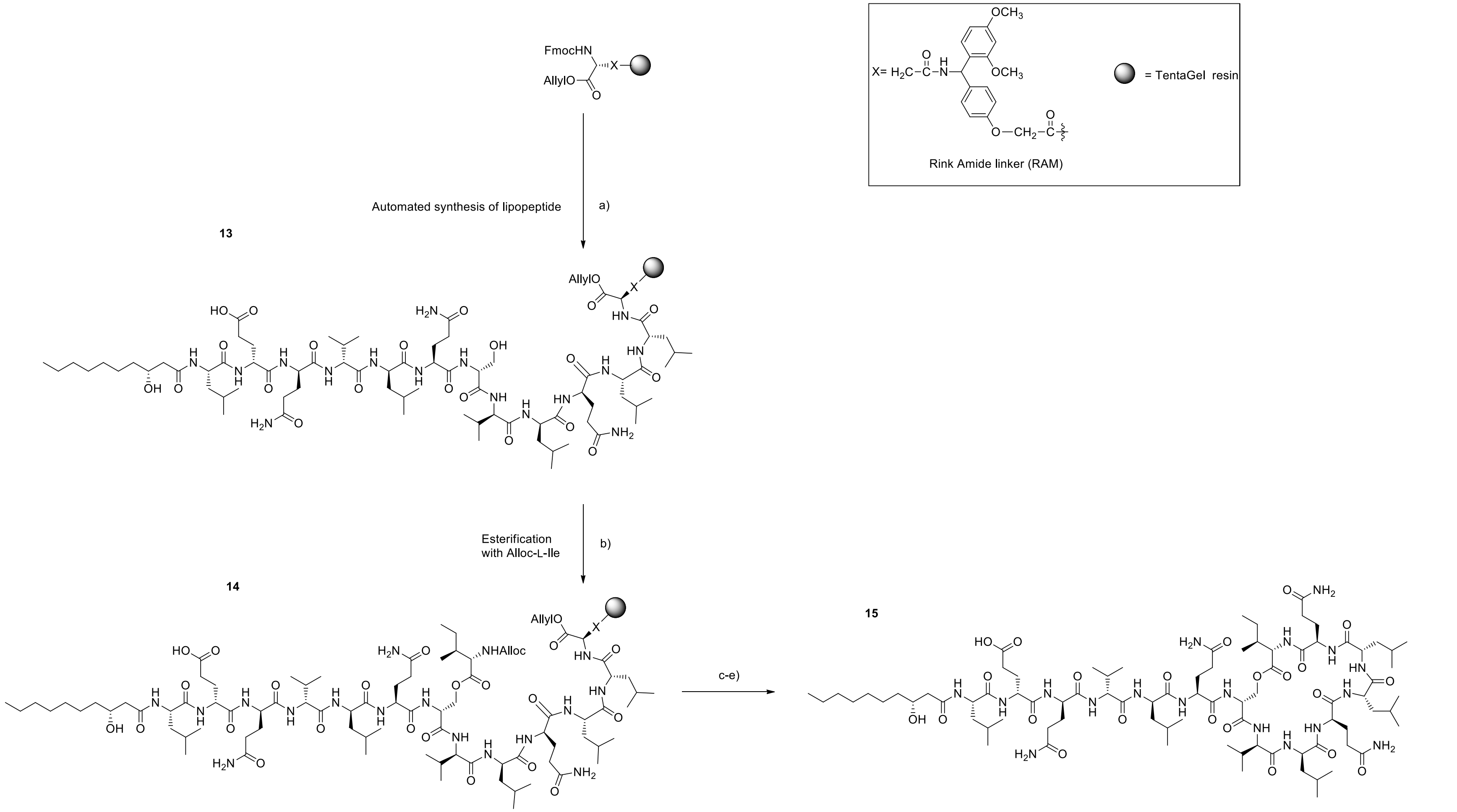

Figure S52: Schematic scheme of the synthesis of xantholysin A. Conditions: a) (i) 20% piperidine, DMF; (ii) Fmoc-AA-OH, HBTU, DIPEA, DMF; b) Alloc-L-Ile, DIC, DMAP, DMF; d) [$Pd(PPh_3)_4$], $PhSiH_3$, CH_2Cl_2; e) HATU, HOBt, DIPEA, DMF; f) 0.1 M HCl in HFIP + 1% TIS

Automated synthesis towards (13)

Automated solid phase peptide synthesis was done on a Syro apparatus. In a C→N fashion, Fmoc-D-Glu(OH)-OAllyl, Fmoc-L-Leu (x2), Fmoc-D-Gln(Trt), Fmoc-D-Leu, Fmoc-D-Val, Fmoc-D-Ser(OH), Fmoc-L-Gln(Trt), Fmoc-D-Leu, Fmoc-D-Val, Fmoc-D-Gln(Trt), Fmoc-D-Glu(OtBu), Fmoc-L-Leu were coupled onto the Rink Amide resin (50 mg, 0.69mmole/g). The automated synthesis included the TBS-protected 3-(R)-hydroxydecanoic acid to cap the N-terminus. After the automated synthesis was completed, the resin was washed thoroughly with use of THF (3x), DMF (6x) and DCM (3x). Afterwards, the completion of the reaction was checked by a small cleavage test and the obtained peptide was subjected to LC-MS analysis.

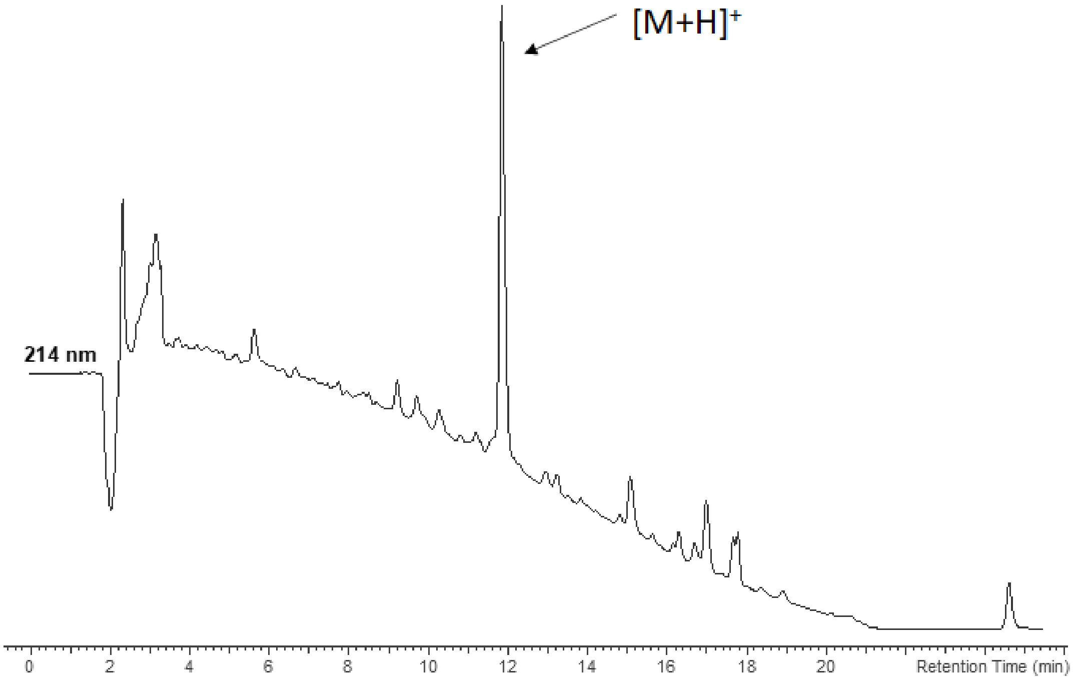

Figure S53: LC-MS chromatogram of (**13**), Kinetex C18, linear gradient of 0-100% acetonitrile in 20'. $[M+H]^+$ was determined to be 1721.1, matching the proposed chemical formula of $C_{81}H_{141}N_{17}O_{23}$.

Esterfication with Alloc-L-Ile towards (14)

The Steglich esterification was carried out by treating the peptidyl resin with 5eq. of Alloc-L-Ile-OH, 5 eq. pyridine and 0.20 eq. DMAP in dry DMF. The mixture was let to shake for 2x 24h at room temperature. The beads were extensively washed with 8x DMF, 8x DCM and 4x Et_2O. The reaction completion was verified using a test cleavage.

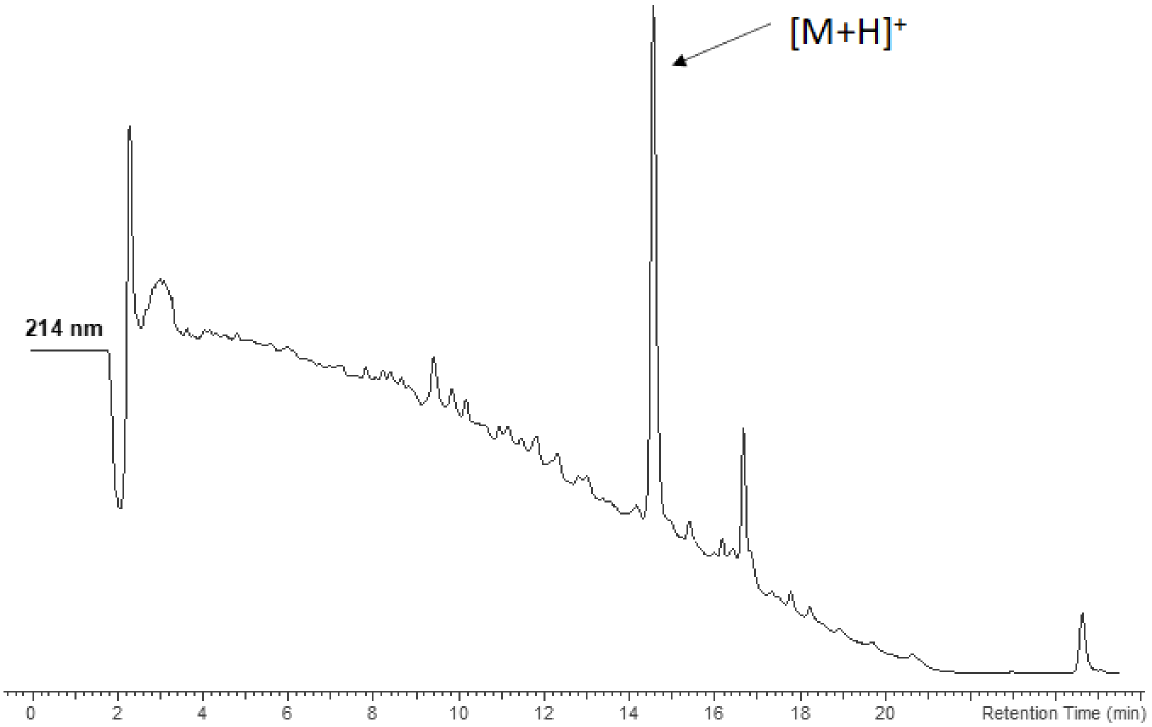

Figure S54: LC-MS chromatogram of (**14**), Luna C18, linear gradient of 50-100% acetonitrile in 15'. $[M+H]^+$ was determined to be 1918.8 Da, matching the proposed chemical formula of $C_{91}H_{156}N_{18}O_{26}$.

Cyclization and subsequent cleavage and deprotection towards (15)

In order to perform the on-resin cyclization, HATU (135.92mg, 0.35mmol, 5equiv.) and HOBt.2H_2O (54.74mg, 0.36mg, 5 equiv.) were dissolved in 1,3ml dry DMF. Afterwards, DIPEA (62.25 µL, 0.36 mmol, 5equiv.) was added and the solution was sonicated until fully dissolved. Under inert atmosphere, the solution was transferred to the beads and subsequently the solution, while shielded from incident light, shaken for 4h. The beads were washed 6x DMF, 6x MeOH, 6x DCM and 2x Et_2O. To ensure full completion, a small test cleavage was performed.

The cyclized peptides were cleaved off by using a cleavage cocktail consisting of 0.1M HCl in HFIP +1% TIS. The cleavage reaction was let to shake for 5 hours, ensuring full deprotection of the peptide. The obtained crude mixture was analyzed using LC-MS (S33).

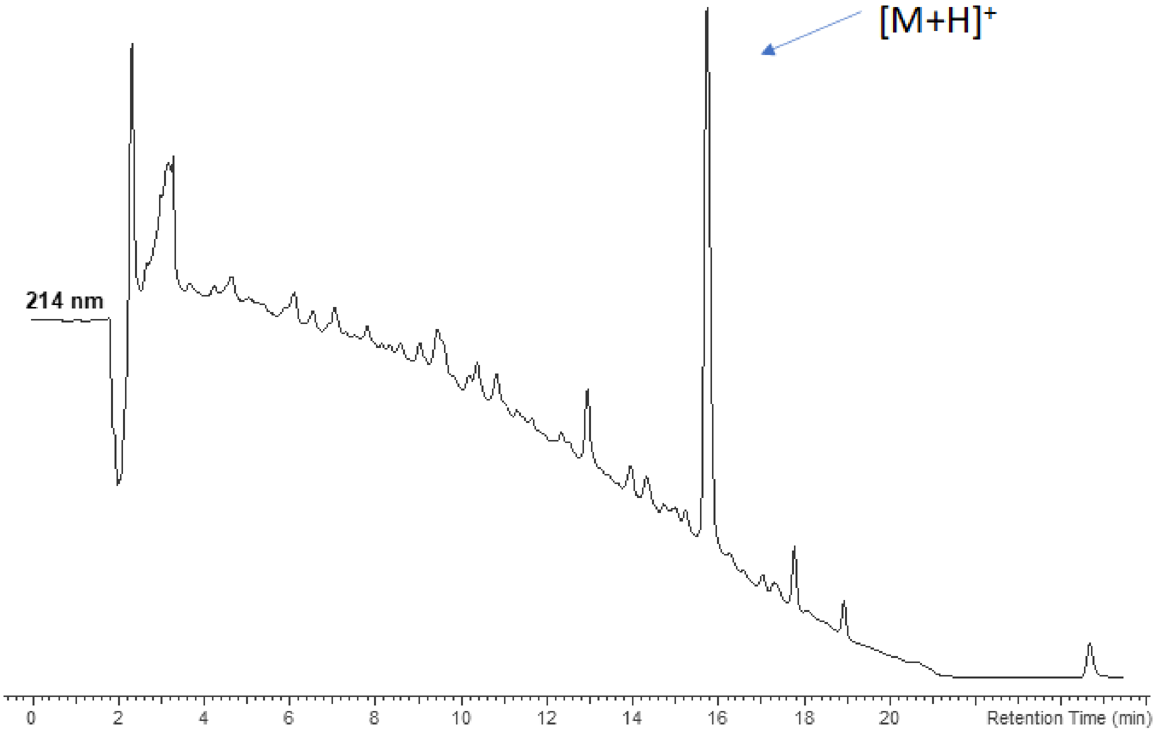

Figure S55: LC-MS chromatogram of the crude mixture containing (**15**), Luna C18, linear gradient of 50-100% acetonitrile in 15'. $[M+H]^+$ was determined to be 1775.8 Da, matching the proposed chemical formula of $C_{84}H_{146}N_{18}O_{23}$.

Purification of (15):

The crude mixture was dissolved in a minimal amount of methanol prior to subjecting to HPLC purification.This peptide fraction was purified via semi-preparative RP- HPLC analysis (Figure S56), using a two solvent system: H_2O + 0.1% TFA (A) and CH_3CN (B), via application of a linear gradient over 30 minutes going from 25:75 to 0:100 (A:B). The product eluted at 16.51 minutes.

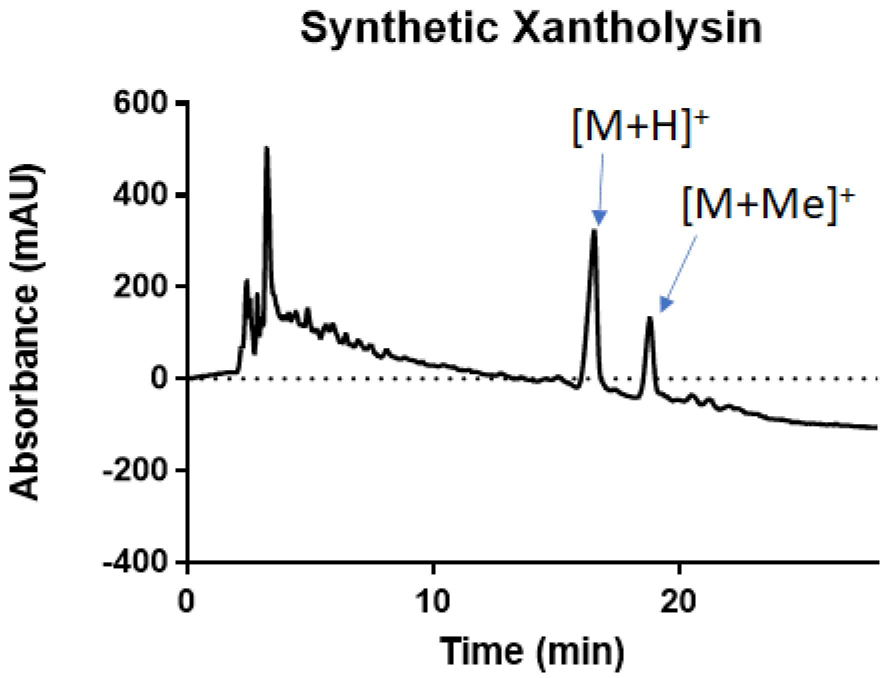

Figure S56: Semi-preparative reversed-phase HPLC chromatogram of synthetic xantholysin A.

NMR characterization of synthetic xantholysin (15)

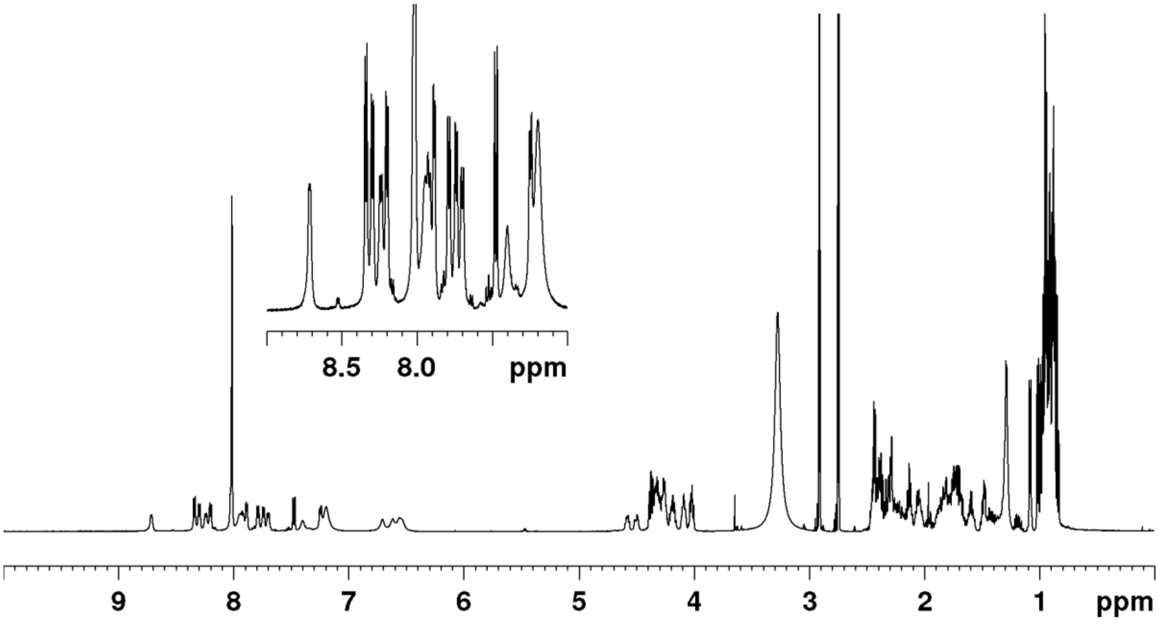

Figure S57: 1D ^{1}H spectrum of synthetic xantholysin A. (DMF-d7, 328K, 700 MHz)

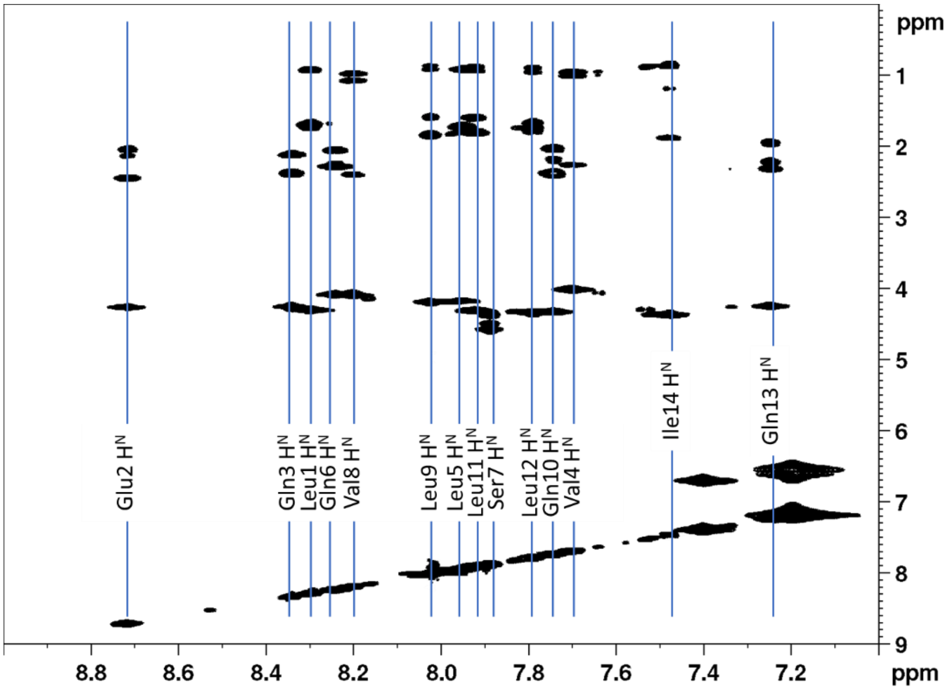

Figure S58: ^{1}H-^{1}H TOCSY traces of the individual amino acids in synthetic xantholysin A. (700MHz, DMF-*d7*, 328K)

Table S30: NMR assignment (^1H and ^{13}C) of synthetic xantholysin A. (700MHz, DMF-d7, 298K)

		**^1H δ [ppm]**	**^{13}C δ [ppm]**			**^1H δ [ppm]**	**^{13}C δ [ppm]**
**HDA**				**L-Gln6**			
	CO				NH	8.25	-
	$CH_2\alpha$	2.44	44.18		$CH\alpha$	4.09	53.92
	$CH\beta$	4.02	68.73		CO		
	$CH_2\gamma$	1.48	37.91		$CH_2\beta1$	2.05	27.08
	CH2 δ1	1.48	25.86		$CH_2\beta2$	2.05	27.08
	CH2 δ2	1.36	25.86		$CH_2\gamma1$	2.38	32.25
	CH2 ε	1.29	29.75		$CH_2\gamma2$	2.22	32.25
	CH2 ζ	1.29	29.75		COδ		
	CH_2 η	1.28	32.12		$NH_2 1$	7.16	-
	CH2 θ	1.29	22.71		$NH_2 2$	6.56	-
	CH_3	0.88	14.00	**D-Ser7**			
	OH				NH	7.89	-
**L-Leu1**					$CH\alpha$	4.58	54.70
	NH	8.30	-		CO		
	$CH\alpha$	4.30	52.87		$CH_2\beta1$	4.50	64.01
	CO				$CH_2\beta2$	4.37	64.05
	$CH_2\beta1$	1.74	40.96	**D-Val8**			
	$CH_2\beta2$	1.67	40.93		NH	8.20	-
	$CH\gamma$	1.75	24.87		$CH\alpha$	4.09	61.89
	$CH_3\delta1$	0.96	23.17		CO		
	$CH_3\delta2$	0.92	21.82		$CH\beta$	2.40	30.27
**D-Glu2**					$CH_3\gamma1$	1.08	19.15
	NH	8.73	-		$CH_3\gamma2$	0.99	19.54
	$CH\alpha$	4.26	55.82	**D-Leu9**			
	CO				NH	8.02	-
	$CH_2\beta1$	2.15	27.03		$CH\alpha$	4.20	54.55
	$CH_2\beta2$	2.05	26.87		CO		
	$CH_2\gamma$	2.45	31.39		$CH_2\beta1$	1.84	40.30
	COδ				$CH_2\beta2$	1.59	40.28
	OH				$CH\gamma$	1.84	25.03
**D-Gln3**					$CH_3\delta1$	0.92	23.13
	NH	8.34	-		$CH_3\delta2$	0.87	21.18
	$CH\alpha$	4.27	55.59	**D-Gln10**			
	CO				NH	7.74	-
	$CH_2\beta$	2.13	27.27		$CH\alpha$	4.33	54.44
	$CH_2\gamma$	2.38	32.33		CO		
	COδ				$CH_2\beta1$	2.19	27.26
	$NH_2 1$	7.40	-		$CH_2\beta2$	2.04	27.29
	$NH_2 2$	6.70	-		$CH_2\gamma$	2.39	32.55
**D-Val4**					COδ		
	NH	7.70	-		$NH_2 1$	7.21	-
	$CH\alpha$	4.02	61.87		$NH_2 2$	6.53	-
	CO			**L-Leu11**			
	$CH\beta$	2.27	27.98		NH	7.92	-
	$CH_3\gamma1$	1.01	19.56		$CH\alpha$	4.32	53.63
	$CH_3\gamma2$	0.96	19.21		CO		
**D-Leu5**					$CH_2\beta1$	1.81	41.21
	NH	7.95	-		$CH_2\beta2$	1.60	41.20
	$CH\alpha$	4.18	54.70		$CH\gamma$	1.84	24.86
	CO				$CH_3\delta1$	0.94	22.93
	$CH_2\beta$	1.71	40.36		$CH_3\delta2$	0.90	21.94
	$CH\gamma$	1.74	25.09				
	$CH_3\delta1$	0.94	22.69				
	$CH_3\delta2$	0.89	21.29				

Table S29 continued: NMR assignment (^{1}H and ^{13}C) of synthetic xantholysin A. (700MHz, DMF-d7, 298K)

		^{1}H δ [ppm]	^{13}C δ [ppm]			^{1}H δ [ppm]	^{13}C δ [ppm]
**L-Leu12**				**L-Ile14**			
	NH	7.79	-		NH	7.48	-
	CHα	4.34	53.24		CHα	4.37	57.51
	CO				CO		
	CH_2β1	1.72	40.61		CHβ	1.89	37.08
	CH_2β2	1.72	40.61		CH_2γ1	1.41	25.41
	CHγ	1.77	25.05		CH_2γ2	1.19	25.41
	CH_3δ1				CH_3γ	0.88	15.84
	CH_3δ2				CH_3δ	0.85	11.15
**D-Gln13**							
	NH	7.25	-				
	CHα	4.26	54.29				
	CO						
	CH_2β1	2.32	27.31				
	CH_2β2	1.96	27.31				
	CH_2γ1	2.31	32.27				
	CH_2γ2	2.22	32.27				
	COδ						
	$NH_2$1	7.17	-				
	$NH_2$2	6.62	-				

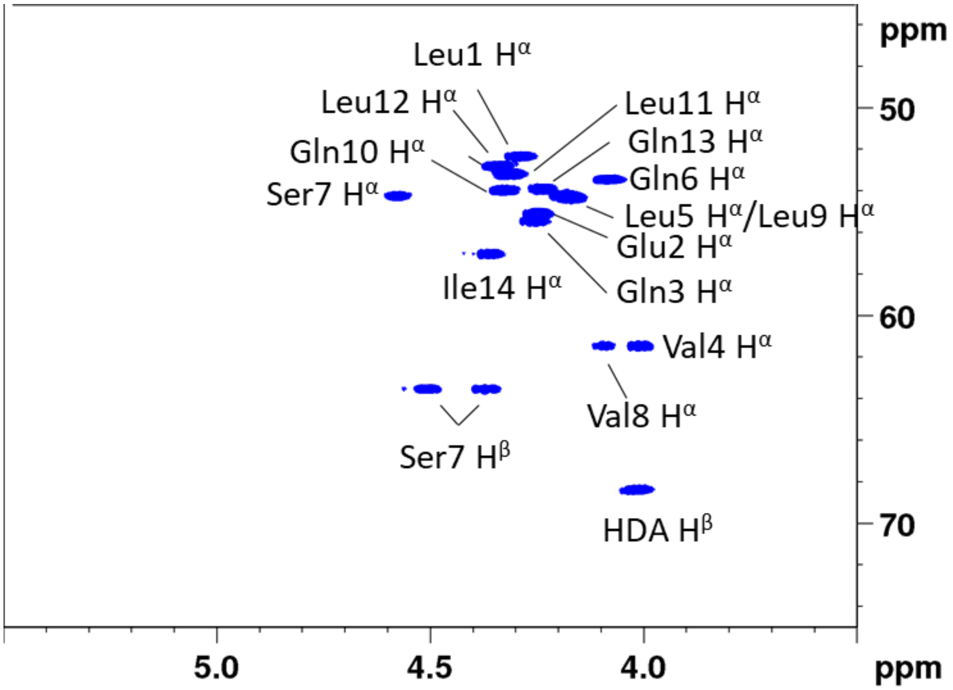

Figure S59: ^{1}H-{^{13}C} HSQC comparison of the (C-H)α fingerprint region. Comparison of the natural xantholysin A (14:8) produced by *P. mosselii* BW11M1 (black) with that produced by *Pseudomonas* sp. COR51 (blue). DMF-d7, 328K, 700MHz.

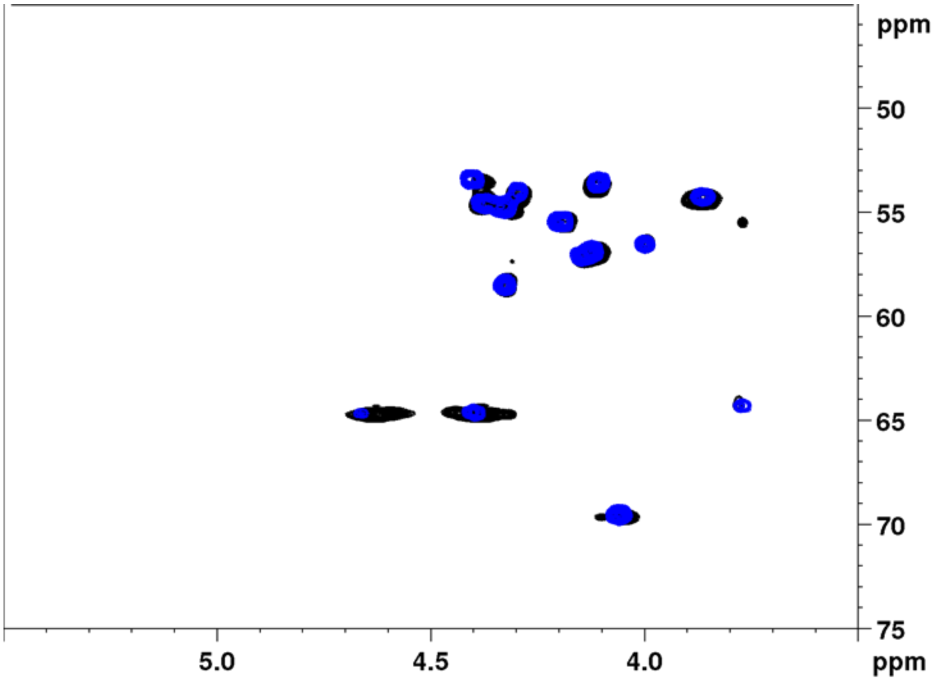

Figure S60: ^{1}H-{^{13}C} HSQC comparison of the (C-H)α fingerprint region. Comparison of the natural xantholysin A (14:8) produced by *P. mosselii* BW11M1 (black) with that produced by *Pseudomonas* sp. 250J (blue). MeOH-d3, 298K, 700MHz.

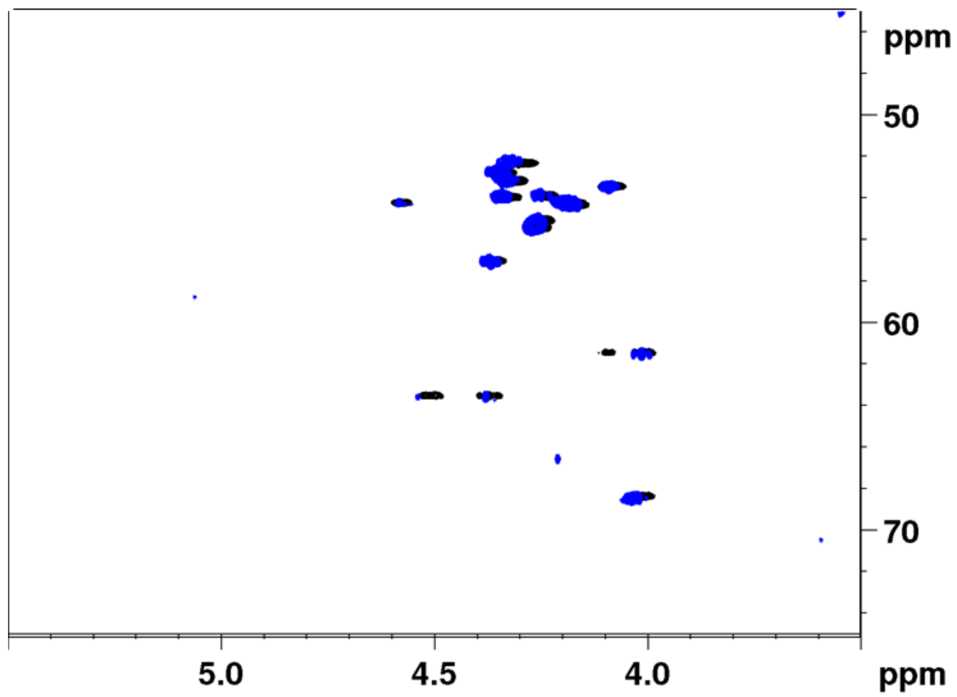

Figure S61: ^{1}H-{^{13}C} HSQC comparison of the (C-H)α fingerprint region. Comparison of the natural xantholysin A (14:8) produced by *P. mosselii* BW11M1 (black) with that produced by *Pseudomonas xantholysinigenes* (blue). DMF-d7, 328K, 700MHz.

Quantifying the (dis)similarity in NMR fingerprints

Using a suitable metric, one could quantify the spectral similarity between two fingerprints. Typically, the difference in ^{1}H ($\Delta^{1}H$) or ^{13}C shifts ($\Delta^{13}C$) can be used. In some cases, the ^{13}C chemical shift difference for a particular α(C–H) is modest while that of its ^{1}H is quite distinct. Therefore, we find that combined use of ^{1}H and ^{13}C chemical shifts provides the best differentiation. To resort to a single value per residue, we use the Euclidian chemical shift distance $d = SQRT[(\Delta^{1}H)^2+(\Delta^{13}C)^2]$ between two peaks to be matched in the ^{1}H-^{13}C HSQC fingerprint. (Applied to the case of bananamide SWRI103 (Figure S62A) and orfamide CMR5c (Figure S62B), it supports the attribution of stereochemistry in a more quantitative way.

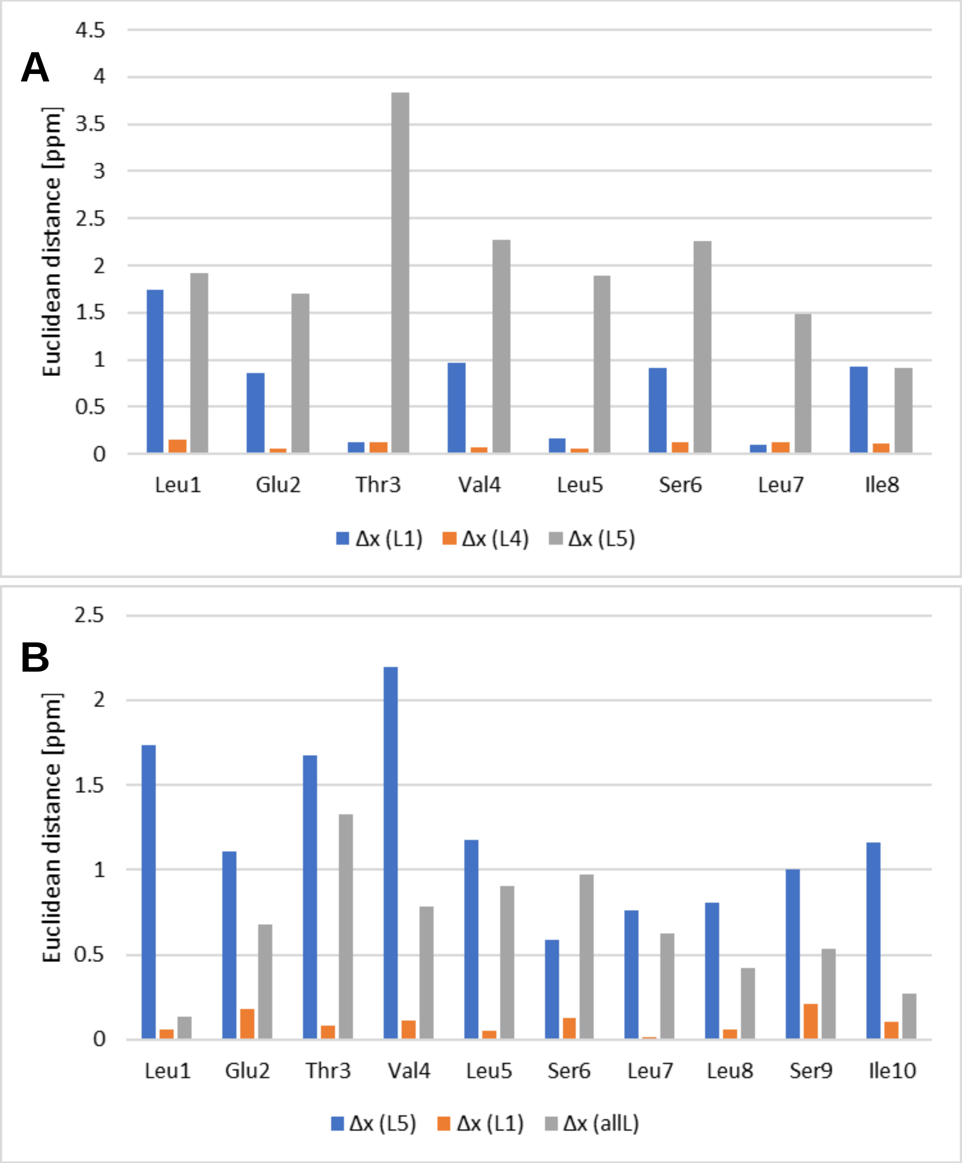

Figure S62: A) Euclidean distance between α(C-H) pairs of bananamide SWR-103, comparing the natural compound with the synthetic (8:6) L1, (8:6) L4 and (8:6) L5 compounds. B) Euclidean distance between α(C-H) pairs of orfamide CMR5c, comparing the natural compound with the synthetic (10:8) L1, (10:8) L5 and (10:8) L1L5 compounds.

Additionally, by using the Sum of the Squared Deviations (SSD) of ^{1}H and ^{13}C chemical shifts of the backbone CH^{α} over all residues, a single numerical value can be obtained per pair of matched spectra and used to rank the various compounds according to increasing SSD value to reveal decrasing similarity. While the choice of a threshold to keep or discard matched sets, for instance for visual analysis, is rather arbitrary, the range of values obtained convincingly demonstrates that for a pair of spectra from identical molecules, the SSD is markedly lower than for mismatched CLiP pairs. For instance, the SSD values for the various synthetic bananamides amount to 6.43, 0,09 and 38.18 respectively for the L1, L4 and L5 analogues, the L4 one being identical to the natural compound. In case of the orfamides, the SSD values are 0.13, 17.19 and 5.54 for (10:8) L1, (10:8) L5 and (10:8) L1L5, respectively.

References

1. Cautain B, *et al.* (2015) Identification of the lipodepsipeptide MDN-0066, a novel inhibitor of VHL/HIF pathway produced by a new *Pseudomonas* species. *PLoS One* 10(5):e0125221.
2. D'Aes J, *et al.* (2014) To settle or to move? The interplay between two classes of cyclic lipopeptides in the biocontrol strain *Pseudomonas* CMR12a. *Environ. Microbiol.* 16(7):2282-2300.
3. Gross H, *et al.* (2007) The genomisotopic approach: a systematic method to isolate products of orphan biosynthetic gene clusters. *Chem. Biol.* 14(1):53-63.
4. Jang JY, *et al.* (2013) Identification of orfamide A as an insecticidal metabolite produced by *Pseudomonas protegens* F6. *J. Agric. Food Chem.* 61(28):6786-6791.
5. Ma Z, *et al.* (2016) Biosynthesis, chemical structure, and structure-activity relationship of orfamide lipopeptides produced by *Pseudomonas protegens* and related species. *Front. Microbiol.* 7.
6. Chan X-Y, Hong K-W, Yin W-F, & Chan K-G (2016) Microbiome and biocatalytic bacteria in Monkey Cup (*Nepenthes Pitcher*) digestive fluid. *Sci. Rep.* 6(1):20016.
7. Girard L, *et al.* (2021) Transporter Gene-mediated Typing for Detection and Genome Mining of Lipopeptide-producing *Pseudomonas*. *Applied Environmental Microbiology*:Aem0186921.
8. Li W, *et al.* (2013) The antimicrobial compound xantholysin defines a new group of *Pseudomonas* cyclic lipopeptides. *PLoS One* 8(5):e62946.
9. Lim DJ, *et al.* (2017) Identification of lipopeptide xantholysins from Pseudomonas sp. DJ15 and their insecticidal activity against Myzus persicae. *Entomological Research* 47(6):337-343.
10. Molina-Santiago C, *et al.* (2015) Efflux pump-deficient mutants as a platform to search for microbes that produce antibiotics. *Microb. Biotechnol.* 8(4):716-725.
11. Oni FE, *et al.* (2019) Fluorescent Pseudomonas and cyclic lipopeptide diversity in the rhizosphere of cocoyam (Xanthosoma sagittifolium). *Environmental microbiology*.
12. De Vleeschouwer M, *et al.* (2014) Rapid Total Synthesis of Cyclic Lipodepsipeptides as a Premise to Investigate their Self-Assembly and Biological Activity. *Chemistry–A European Journal* 20(25):7766-7775.
13. De Vleeschouwer M, *et al.* (2014) Rapid total synthesis of cyclic lipodepsipeptides as a premise to investigate their self-assembly and biological activity. *Chem. Eur. J.* 20(25):7766-7775.
14. De Vleeschouwer M, Martins JC, & Madder A (2016) First total synthesis of WLIP: on the importance of correct protecting group choice. *J. Pept. Sci.* 22(3):149-155.
15. De Vleeschouwer M, *et al.* (2020) Identification of the Molecular Determinants Involved in Antimicrobial Activity of Pseudodesmin A, a Cyclic Lipopeptide From the Viscosin Group. *Front. Microbiol.* 11(646).
